# Supplementary material for: Theories used to explain care-leavers’ journey out of care: A scoping review
Source: PLoS One. 2025 Jun 13;20(6):e0325776. doi: 10.1371/journal.pone.0325776 (PMC12165428; doi:10.1371/journal.pone.0325776)
Supplement: S1 Table — (DOCX) [file pone.0325776.s002.docx]

**Table 1:** **Data extraction table: Theories used to explain care-leavers’ journey out of care (2015-2023)**

| **Reference** | **Theory** | **How theory used** | **TG** | **Theory guided** | **Practical application** |
| --- | --- | --- | --- | --- | --- |
| Edwards, T., Chowdhury, R., Laylor, A., Parada, H., & King, B. (2023). Pushed, dropped, or fleeing from care: The narratives and adultification of black youth who have aged out of Ontario’s child welfare system. *Child & Youth Services*, Advance online publication. <https://doi.org/10.1080/0145935X.2023.2173565> | Adultification | Adultification (Davis & Marsh) refers to the experience of Black children who are severed from childhood and thought of in more adultlike terms (older and less innocent) than children from other racial or ethnic groups. This theory was used to interpret the narrative findings of Black youth who had transitioned out of care. While the emerging themes were not explicitly framed within this theoretical concept, the findings were interpreted within the frame. | 1 | Theory is used throughout the study from design to interpretation | Black care-leavers need additional support to navigate a white-dominated society where they are vulnerable to racism and discrimination. They need a stronger network of reliable support, to avoid ‘falling through the cracks’. |
| Göbel, S., Hadjar, A., Karl, U., & Jäger, J. A. (2021). Agency and the school-to-work transition of care leavers: A retrospective study of Luxembourgish young people. *Children and Youth Services Review, 122*. <https://doi.org/10.1016/j.childyouth.2020.105636> | Agency | Relational agency (Emirbayer & Mische) differs from classical agency (self-efficacy). It operates at the interface between structure and agency. “Agency is situated in social practices.” Habit, imagination, and judgement are components of agency. | 1 | Study set out to investigate these theoretical constructs | It is crucial to support care-leavers with continued or completing education, through social, emotional, practical and financial support, because it is central to fostering agency and positive future development. |
| Macleod, G., Dallas-Childs, R., Brough, C., & Toye, M. (2021). ‘She just got me’: Supporting care experienced young people negotiating relationships and identities at school. *Journal of Research in Special Educational Needs, 21*(S1), 25-35. <https://doi.org/10.1111/1471-3802.12543> | Agency | The study examines the complex relationship between care experience, mental health and exclusion from school and how young people negotiate these different experiences and identities. Agency (Berridge) was a theme identified in the analysis. | 2 | Mentioned agency twice, once in the analysis and discussion section of the article. | It was recommended that care-leavers are active agents for positive youth development and need to create the right conditions for their positive self-identification. |
| Van Breda, A. D. (2016). The roles of agency and structure in facilitating the successful transition out of care and into independent living. *Social Work Practitioner-Researcher, 28*(1), 36-52. <https://doi.org/10.25159/2415-5829/1349> | Agency – and structure | This article explores the findings from recent South African care-leaving research on the contributions of agency and structure to the successful transition out of care and into independent living. Agency and structure are found to be closely interrelated (Côté & Bynner), in alignment with relational sociological theory. | 2 | It is used to show the inter-relatedness of the two concepts of agency and structure. | Author states that structural resources need to be available in the environment and, in addition, individuals need the agency to identify, access and utilise these resources. Both agency and structure need to be developed while in care. |
| Barratt, C., Appleton, P., & Pearson, M. (2020). Exploring internal conversations to understand the experience of young adults transitioning out of care. *Journal of Youth Studies, 23*(7), 869-885. <https://doi.org/10.1080/13676261.2019.1645310> | Agency – internal conversations | This study builds on a previous study using this theory with care-leavers by Hung and Appleton [also in this systematic review] to explore how reflexivity (internal conversations, Archer) shapes the transition out of care. These conversations are thought to mediate agency (Archer) and structure. The authors explore care-leavers’ internal conversations to see how they experience and make sense of their care-leaving transition and how they act/react to the world around them. Most participants did demonstrate reflexivity, but several also reported that internal conversations were unhelpful to them, even harmful. The internal conversations did help shed light on how care-leavers navigate between agency and structure. | 1 | Theory selected upfront and study aimed to determine its resilience to care-leavers | Pathway planning needs to be supplemented with the development of care-leavers planning capacities, particularly developing confidence in their ability to have an internal conversation about their future. |
| Hung, I., & Appleton, P. (2016). To plan or not to plan: The internal conversations of young people leaving care. *Qualitative Social Work, 15*(1), 35-54. <https://doi.org/10.1177/1473325015577408> | Agency – internal conversations | Explore the degree to which care-leavers plan, based on internal conversations (part of Archer’s theory of agency and reflexivity). Agency is enabled by reflexivity, which takes the form of internal conversations. Planning is a key function of internal conversations. While Archer’s active and passive agency were evident, the researchers found a new survival-oriented agency that seemed prominent in dealing with contextual adversity and involved high levels of self-reliance. Most participants did not like planning. | 1 | Study set out to explore the applicability of a theory | Given the lack of enthusiasm for planning, the approach to pathway planning may need to be reconsidered. |
| Gundersen, T. (2021). Embedded in relations: Interactions as a source of agency and life opportunities for care-experienced young adults. *Children & Society, 35*(5), 680-693. <https://doi.org/10.1111/chso.12434> | Agency – relational | This study explores how relational agency (Burkitt) develops – “processes that underlie and influence agency”. Relational agency emphasises relationships and temporality. | 1 | Study sought to answer a theoretical question | Agency develops in the context of care relationships – relationship-based care is thus essential. Interdependence aligns with relational agency. |
| Goemans, M., van Breda, A. D., & Kessi, S. (2021). Experiences of young people preparing to transition out of cluster foster care in South Africa. *Child and Adolescent Social Work Journal, 38*(2), 227-237. <https://doi.org/10.1007/s10560-020-00704-1> | Ambiguous loss | Ambiguous loss (Boss) refers to an unclear quality of relatedness – in and out at the same time. Ambiguous loss plays out within the foster family as well – do I truly belong or am I just a client. | 2 | Study did not set out to investigate this theory, but findings were made sense of through theory use | Ambiguity is a common experience among care-leavers and is not helpful for their belonging and development. |
| Gwenzi, G. D. (2023a). Care leavers’ notions of the ‘ideal’ family in Zimbabwe. *Journal of Family Studies, 29*(3), 1428-1446. <https://doi.org/10.1080/13229400.2022.2058411> | Ambiguous loss | Ambiguous loss (Boss) helps explain the conflicted experiences of many children in care. The tension between the physical absence of the family of origin but their psychological presence. Study was interested in the notion of an ‘ideal’ family. | 2 | Study sought to explicate ambiguous loss, but not pulled into the discussion | No theory-informed practice recommendations provided. |
| Appleton, P. (2020). Anchors for deliberation and shared deliberation: Understanding planning in young adults transitioning from out-of-home care. *Qualitative Social Work, 19*(5-6), 1130-1146. <https://doi.org/10.1177/1473325019869810> | Anchors for deliberation | Anchors for deliberation are “the idea that certain plan-states both concern what matters in the sense of having weight in our deliberative thought, and when functioning properly tie together our thought and action in relevant ways, both synchronically and diachronically” (Bratman). Focusing on and understanding what is most important to a young person may better support them in their early transitions out of care. | 1 | This is a theoretical paper | Raising the voice of young people in planning processes. |
| Edwards, T., Chowdhury, R., Laylor, A., Parada, H., & King, B. (2023). Pushed, dropped, or fleeing from care: The narratives and adultification of black youth who have aged out of Ontario’s child welfare system. *Child & Youth Services*, Advance online publication. <https://doi.org/10.1080/0145935X.2023.2173565> | Anti-Black Racism | Anti-Black Racism theory (Cole) helps to frame the experiences of Black care-leavers within the social context of anti-Black racism, which can pervasively shape their lived experience, including their experience of leaving care. This theory was used to interpret the narrative findings of Black youth who had transitioned out of care. While the emerging themes were not explicitly framed within this theoretical concept, the findings were interpreted within the frame. | 1 | Theory is used throughout the study from design to interpretation | Black care-leavers need additional support to navigate a white-dominated society where they are vulnerable to racism and discrimination. They need a stronger network of reliable support, to avoid ‘falling through the cracks’. |
| Doucet, M., Pratt, H., Dzhenganin, M., & Read, J. (2022). Nothing about us without us: Using Participatory Action Research (PAR) and arts-based methods as empowerment and social justice tools in doing research with youth ‘aging out’ of care. Child Abuse & Neglect, 130, 105358. <https://doi.org/10.1016/j.chiabu.2021.105358> | Arrested adulthood | Adopting participatory action research and arts-based methods meant declining to use care-leaving theories that does not recognise the unique experiences of individuals or that boxes individuals. Arrested adulthood (Côté) describes people in quite negative terms (unstable, undecided, etc.) which defines young people as a social problem to be studied and controlled. | 3 | Term mentioned only once in the introduction. | The study rejected this theory, so no practical application beyond rejecting it. |
| Adley, N., & Jupp Kina, V. (2017). Getting behind the closed door of care leavers: understanding the role of emotional support for young people leaving care. *Child & Family Social Work, 22*(1), 97-105. <https://doi.org/10.1111/cfs.12203> | Attachment | Attachment (Howe) mentioned as a theory and that “it is vital to consider and explore early attachment figures and pre-care relationships within a broader understanding of an individual’s resilience and ability to engage with or desire further emotional support.” Used to interpret a quote from one participant about how earlier relationships influence present relationships. | 3 | Mentioned briefly up front, but used only once and superficially in the findings. | No theory-informed practice recommendations provided. |
| Bogdanova, E. (2017). Russian SOS Children’s Villages and deinstitutionalisation reform: Balancing between institutional and family care. *Zhurnal Issledovanii Sotsialnoi Politiki [The Journal of Social Policy Studies], 15*(3), 395-406. <https://doi.org/10.17323/727-0634-2017-15-3-395-406> | Attachment | Attachment theory (Bowlby) is used to show that SOS Children’s Villages avoid the pitfalls of institutional care, by maintaining family-like care settings. Attachment relationships require small, intimate care settings with stable caregivers. | 2 | Study is informed by theory but its place is not central | Recommend that SOS Children’s Villages are aligned with this theory and should not be closed down or restructured under the deinstitutionalisation agenda |
| Ferguson, L. (2018). “Could an increased focus on identity development in the provision of children’s services help shape positive outcomes for care leavers?” A literature review. *Child Care in Practice, 24*(1), 76-91. <https://doi.org/10.1080/13575279.2016.1199536> | Attachment | In this literature review, attachment theory emerges as showing how early disruptions in attachment (often the case with care-leaver) are internalised and impact on subsequent relationships during care-leaving. This has an impact on identity development, which is rooted in relationships and internal working models. Later positive attachments may undo some of this damage. | 2 | Article discusses theory but is not driven by it | No theory-informed practice recommendations provided. |
| Goyette, M., Blanchet, A., Esposito, T., & Delaye, A. (2021). The role of placement instability on employment and educational outcomes among adolescents leaving care. *Children and Youth Services Review, 131*, 106264. <https://doi.org/10.1016/j.childyouth.2021.106264> | Attachment | The study draws on attachment theory (Ainsworth & Bowlby). An early, consistent caregiver (even just one) enables young people to develop a sense of security, trust in the world, and positive neurological and social development. Unstable placements are thought to undermine the development of attachment. | 2 | The study’s topic is justified using theory, but findings are not linked to theory. | Placement instability increases the likelihood of dropping out of school early and not acquiring work experience. Placement stability is thus desirable. |
| Heyman, J. C., White-Ryan, L., Kelly, P., Farmer, G. L., Leaman, T. L., & Davis, H. J. (2020). Voices about foster care: The value of trust. *Children and Youth Services Review, 113*, 104991. <https://doi.org/10.1016/j.childyouth.2020.104991> | Attachment | Interested in the factors that might protect CL from homelessness. Attachment (Bowlby) provides insight into the vulnerability of care-leavers based on early fractures of important relationships. Social connections emerge as important for care-leavers. Attachment and trust are foregrounded in the discussion – need for secure relationships with trusted adults. | 1 | Study informed by theory | Train foster parents in positive youth development. Match youth to appropriate foster parents. Build trust. Develop communication skills. |
| Hyde, A., Fullerton, D., Lohan, M., Dunne, L., & Macdonald, G. (2017). Young people’s views on the impact of care experiences on their ability to form positive intimate relationships. *Adoption & Fostering, 41*(3), 242-253. <https://doi.org/10.1177/0308575917714375> | Attachment | Bowlby and Ainsworth’s models of attachment provide a foundation to understand care-leavers’ experiences of family and care relationships. The study finds that disrupted family of origin attachments negatively impact later intimate relationships and sexual health for some care-leavers. | 1 | Study informed by theory | Promote stable, secure and trustworthy relationships with caregivers. |
| Kelly, P., Heyman, J., Zhai, F., & Salazar, A. (2023). Social and emotional supports during college years: Associations with post-college outcomes among alumni of foster care. *Child and Adolescent Social Work Journal, 40*(5), 607-621. <https://doi.org/10.1007/s10560-021-00806-4> | Attachment | Attachment theory (Miranda) helps understand the social and emotional needs satisfied through meaningful relationships. This is important, as many youth in care experienced child abuse and neglect from primary caregivers. Attachment theory is used to explain enablers for care-leavers after completion of tertiary education (post-college). Higher levels of social support and socialisation in college were associated with higher self-perceptions of happiness after college and lower use of public assistance. | 1 | Study was conducted to answer theoretically informed questions | Campus support programmes should be offered to care-leavers to facilitate transition into college, build relational networks, and graduate. Programmes should help care-leavers in college build meaningful and sustainable social networks. Social supports formed during college should be continued after graduation. Financial support for those in education should be extended beyond age 21. |
| Malvaso, C., Delfabbro, P., Hackett, L., & Mills, H. (2016). Service approaches to young people with complex needs leaving out-of-home care. *Child Care in Practice, 22*(2), 128-147. <https://doi.org/10.1080/13575279.2015.1118016> | Attachment | Study focuses on service providers’ views of the challenges faced by care-leavers. Theme 2 concerned mental health, behaviour and adjustment, which may be related to fractured relationships with significant others. Attachment theory (Andersson) is used to explain that these early disruptions of attachment continue to impact people later in life. | 3 | Theory mentioned only briefly to explain one finding. | Recommend greater attention to relationship building between staff and care-leavers, and between care-leavers and significant others (notably family). |
| Mayall, H., O’Neill, T., Worsley, A., Devereux, R., Ward, S., & Lynch, D. (2015). The experiences of care leavers (post-care adults) in social work education. *Social Work Education, 34*(2), 151-164. <https://doi.org/10.1080/02615479.2014.962019> | Attachment | Attachment theory (not cited) was mentioned as being difficult for care-leavers studying social work. It included various assumptions about early childhood relationships (that the lack of an early, good, stable relationship leaves you scarred for life) that triggered discomfort and increasing isolation. | 3 | Theory not used to guide the study | Social work lecturers should be more nuanced in the way they teach attachment theory, considering there might be students in class who have attachment issues. |
| Mertz, M., & Andersen, S. H. (2017). The hidden cost of foster-care: new evidence on the inter-generational transmission of foster-care experiences. *British Journal of Social Work, 47*(5), 1377-1393. <https://doi.org/10.1093/bjsw/bcw132> | Attachment | When children have incoherent (rather than coherent) recollections of negative parenting experiences, the child is more likely to imitate them and enact them when they become a parent, which can facilitate intergenerational transmission of foster care experiences. This emphasises an intergenerational route via parenting experiences. Even when controlling for parental resources, parental foster care experience still predicts child’s foster care experience, perhaps resulting from attachment deficits (Kaufman & Zigler). | 1 | Study driven by theory | No theory-informed practice recommendations provided. |
| Pasli, F., & Aslantürk, H. (2023). Examining the family belonging of adults with institutional care experience in childhood. *Asia Pacific Journal of Social Work and Development*, Advance online publication. <https://doi.org/10.1080/02185385.2023.2233470> | Attachment | The aimed to examine the sense of family belonging of individuals with childhood institutional care experience through personal information, and institutional care and post institutional care variables. Attachment theory (Bowlby) is used as a conceptual basis for understanding belonging. | 1 | Theory is used as a conceptual base. | “The results showed that professionals and policymakers working with adults with childhood institutional care experience should consider the importance of family belonging and related factors in institutional care and adaptation to life after, and understand the importance of transition to family- and community-based care services, which will benefit child welfare.” |
| Refaeli, T., Zeira, A., & Benbenishty, R. (2022). Ten years later: Satisfaction with intimate relationships and parenthood of young people aging out of residential care. *Child Abuse & Neglect, 129*, 105635. <https://doi.org/10.1016/j.chiabu.2022.105635> | Attachment | The study examined two models among Israeli care-leavers a decade after leaving care to estimate factors associated with satisfaction with intimate relationships and with parenthood. Attachment theory (Bowlby) was used to explain how care-leavers’ backgrounds may impact their satisfaction with intimate relationships and parenthood, potentially leading to difficulties in these life domains, necessitating the identification of contributing factors. | 1 | This theory served as the framework of the study. | Given that being in intimate relationships and becoming a parent can revive memories of care-leavers’ own adverse childhoods, there is a particular need for interventions to support this group. Further research is required into care-leavers’ intimate relationships to understand what kind of support they need. |
| Richardson, V., Boylan, J., & Brammer, A. (2017). Contact, welfare and children in care: revisiting the significance of birth family relationships after finding significant harm. *Journal of Social Welfare & Family Law, 39*(1), 67-82. <https://doi.org/10.1080/09649069.2016.1275124> | Attachment | Attachment theory (Bowlby) is used to understand continuing birth family involvement in the child’s life once the child is living in care. Both involvement and lack of contact can be harmful for the child in care. | 3 | Mentions of the attachment theory is made once in the discussion and once in the conclusion sections of the study. | The authors recommend that the childcare policies be adjusted, indicating in detail the contact of the biological parents and there should be a clearer explanation of the long-term role and impact of the child’s relationship with the birth mother and siblings, in the care plan and the independent reviews, which is likely to result in improved long-term emotional well-being. |
| Sulimani-Aidan, Y. (2018a). Assets and pathways in achieving future goals of residential care alumni. *Children and Youth Services Review, 89*, 71-76. <https://doi.org/10.1016/j.childyouth.2018.04.023> | Attachment | The care-leavers’ tendency for self-reliance, could be explained further by Attachment Theory (Bowlby), as care-leavers may have poor early attachments that lead towards excessive self-reliance. | 3 | Mentioned once in analysis of findings. | Authors recommend a need for a “mentoring policy” within residential care placements (p. 76). Positive and meaningful figure, such as a mentor, may compensate for earlier poor attachment relationships |
| Sulimani-Aidan, Y. (2018c). Present, protective, and promotive: Mentors’ roles in the lives of young adults in residential care. *American Journal of Orthopsychiatry, 88*(1), 69-77. <https://doi.org/10.1037/ort0000235> | Attachment | The study explored mentoring relationships in the lives of 140 care-leavers, and the way those relationships influenced their life course. The attachment theory is used at a framework in explaining the link between mentors’ role in the youth’s lives and its impact on their life course. | 1 | Used in the study to also discuss findings. | Authors recommend a need for a “mentoring policy” within residential care placements (p. 76) Thus, as a positive and meaningful figure, such as a mentor, may compensate for earlier poor attachment relationships |
| Sulimani-Aidan, Y., & Paldi, Y. (2020). Youth perspectives on parents’ involvement in residential care in Israel. *Journal of Social Work, 20*(1), 64-82. <https://doi.org/10.1177/1468017318793619> | Attachment | The study, drawing on attachment theory (Bowlby), looks at biological parents’ involvement in the residential placement of Israeli youth who left care. Their parents’ involvement in care and the degree to which the placement staff involved parents. Youth functioning while in care and after leaving was also examined according to their parents’ involvement. | 1 | Study is based on the attachment theory | Parental involvement in care should be promoted. Training to support care workers in involving parents should be provided. Parents should be involved in disengagement planning. |
| Valset, K. (2018). Investigating the link between school performance, aftercare and educational outcome among youth ageing out of foster care: a Norwegian nationwide longitudinal cohort study. *Nordic Social Work Research, 8*(sup1), 79-93. <https://doi.org/10.1080/2156857X.2018.1457557> | Attachment | This longitudinal study looks at the population born 1986–1990 that was placed in long term foster care. Attachment theory (Paulsen & Berg) is used to explain why continuous foster care has a positive effect on education and other forms of aftercare have not and resources that are provided to this age group. | 3 | Mentioned only twice in the article, in the discussion section. | No theory-informed practice recommendations provided. |
| Van Breda, A. D. (2015). Journey towards independent living: A grounded theory investigation of leaving the care of Girls & Boys Town South Africa. *Journal of Youth Studies, 18*(3), 322-337. <https://doi.org/10.1080/13676261.2014.963534> | Attachment | One of the social processes identified in the study is young people striving for authentic belonging, links are made to explain this process by drawing on the attachment theory. | 2 | Mentioned in the findings sections when explaining one of the social processes that emerged from the study. | Author recommends programmes need to teach young people skills on how to identify authentic belonging vs pseudo–belonging or false sense of security. |
| Ward, S., Devereux, R., Mayall, H., O’Neill, T., & Worsley, A. (2017). Care leavers on social work courses: A study of identity management. *Child & Family Social Work, 22*(1), 339-348. <https://doi.org/10.1111/cfs.12244> | Attachment | This study presents the experiences of social work students who were previously care-leavers and explored how they managed their identities, through the time that they were in care, in the period having left care and having been accepted on a social work course. The attachment theory (not cited), one of the teachings of social work, is learned in relation to care-leavers’ poor outcomes. Findings is that lectures about care-leavers dwelt upon poor outcomes for those in care. | 3 | Links made twice only in the analysis section regarding care-leavers’ thoughts on the teaching of social work and care-leavers. | Findings suggested that “The teaching of attachment theory was particularly criticized for being over-deterministic and appearing to suggest that insecure attachments inevitably led to poor outcomes.” (p. 347). Authors made a recommendation to be more sensitive when planning and delivering of social work education. |
| Alderson, H., Brown, R., Copello, A., Kaner, E., Tober, G., Lingam, R., & McGovern, R. (2019). The key therapeutic factors needed to deliver behavioural change interventions to decrease risky substance use (drug and alcohol) for looked after children and care leavers: a qualitative exploration with young people, carers and front line workers. *BMC Medical Research Methodology, 19*, 1-12. <https://doi.org/10.1186/s12874-019-0674-3> | Behaviour Determinants Intervention | Behaviour Determinants Intervention is a theory of change informing an intervention to reduce substance misuse. The theory argues that to reduce substance misuse, the wider social and environmental determinants of lack of family support and supervision and the life experiences that led the child into care would have to be addressed. This model is used to develop positive social networks, improve communication skills within the networks, build the networks’ understanding of the young person, promote supportive for change and reduction of conflict and increase understanding of relapse. The model attends to specific aspects of being a looked after child. | 1 | A theoretically informed theory of change led to the design or adaptation of an intervention | The theory is a practice model, so the whole paper is intrinsically a practice application. |
| Bengtsson, T. T., & Mølholt, A.-K. (2018). Creation of belonging and non-belonging in the temporal narratives of young people transitioning out of care in Denmark. *Nordic Social Work Research, 8*(sup1), 54-64. <https://doi.org/10.1080/2156857X.2018.1494034> | Belonging | Belonging (and non-belonging) (Lähdesmäki et al.) are sociological constructs that refer to the quality of connection with one’s environment. The need to belong is virtually universal. | 1 | The notion of belonging directed data collection and analysis | Belonging and a sense of home are closely connected and need to be fostered. Belonging is relational. |
| Smith, G. P., Darab, S., & Hartman, Y. (2022). Exploring a sense of belonging for some Forgotten Australians as they age. *Community, Work & Family, 25*(2), 137-151. <https://doi.org/10.1080/13668803.2020.1749030> | Belonging | Belonging (Goodwin-Smith et al.) refers to an individuals’ participation in a system or environment so that persons feel themselves to be an integral part of that system or environment. The study explores the experiences of the forgotten Australians as they reported them regarding a sense of belonging in relationships within family, community, and social networks. Findings relate to social disconnection, the construction of safe spaces, and a desire to belong. | 1 | Entire study is based on belonging principles | ‘Priority Access Cards’ should be established by the Federal Government for this population, which should provide access to free legal assistance and comprehensive health cover inclusive of pharmaceutical, optical and dental services. It would prevent the need for re-telling of their stories to authorities and care providers to access services, given the potential for re-traumatisation. |
| Neal, D. (2017). Academic resilience and caring adults: The experiences of former foster youth. *Children and Youth Services Review, 79*, 242-248. <https://doi.org/10.1016/j.childyouth.2017.06.005> | Care | Theory of care (Noddings) emphasizes both caring for and being cared for. “One of the most influential manifestations of the theory of care, was the role of adult supporters, was discussed as a main finding in this study. Positive relationships with an adult can stimulate students’ belief in themselves and the desire to change their academic outcomes, resulting in a stronger academically resilient young people” (p. 245). | 3 | Theory of care mentioned in literature review only. | No theory-informed practice recommendations provided. |
| Bennwik, I.-H. B., & Oterholm, I. (2021). Policy values related to support for care leavers with disabilities. *European Journal of Social Work, 24*(5), 884-895. <https://doi.org/10.1080/13691457.2020.1751589> | Citizenship | Citizenship (Nussbaum) refers to one’s sense of membership of society. People with disabilities often do not feel citizenship. | 1 | Citizenship informed data collection and analysis | Citizenship implies both belonging and obligation, and can lead to an emphasis on individual responsibility (and independence). |
| Turner, L., & Percy-Smith, B. (2020). Care leavers and children’s services: Exploring the utility of communities of practice in theorising transition. *British Journal of Social Work, 50*(6), 1758-1774. <https://doi.org/10.1093/bjsw/bcz108> | Communities of Practice (CoP) | This study provides new ways of understanding the experiences of care-leavers and professionals by examining the social systems they inhabit. It adopts a psychosocial approach to understanding care-leavers’ paths through and out of care by the application of Communities of Practice theory (Lave & Wenger). It understands care-leaving as a negotiation between the individual and their social contexts. | 1 | Study is based on this theory | Enduring and sustained community membership is required beyond leaving care – this could be done by linking care-leavers to community groups in the areas where they live or work. |
| Sulimani-Aidan, Y., Achdut, N., Zeira, A., & Benbenishty, R. (2022). Psychological distress among care leavers during the transition to adulthood: Risk and protective factors throughout their life course. *Child & Family Social Work, 27*(2), 324-339. <https://doi.org/10.1111/cfs.12887> | Conservation of resources | This study explored risk and protective factors contributing to and predicting care-leavers’ psychological distress as emerging adults. Conservation of resources (Hobfoll) as a theoretical framework is used for understanding the factors contributing to care-leavers’ psychological distress. Conservation of resources is based on the premise that human activity is focused on gaining, maintaining and protecting resources. The domains of conservation of resources emerged as relevant for participants’ psychological distress. | 1 | Entire study is based on conservation of resources | Policy and practice need to approach mental health issues in a holistic manner, addressing individual, social and societal levels, and their interaction. |
| Ehlke, C., & Schröer, W. (2023). Fluid transitions: “Weak” constellations of participation in the process of leaving care. *Children and Youth Services Review, 151*, 107036. <https://doi.org/10.1016/j.childyouth.2023.107036> | Constellations of participation | Participation in life can be thought of as constellations (Schnurr) – multiple components contributing to a degree of participation: weak vs strong constellations of participation. The authors argue that there are strong constellations while children are in care, but weak constellations as they transition from out care. The study seeks to see how participation can be more strongly voiced in times of weak constellations. | 1 | The whole paper – a theoretical piece – centres on the theory | No theory-informed practice recommendations provided. |
| Golding, F., Lewis, A., McKemmish, S., Rolan, G., & Thorpe, K. (2021). Rights in records: A *Charter of Lifelong Rights in Childhood Recordkeeping in Out-of-Home Care* for Australian and Indigenous Australian children and care leavers. *The International Journal of Human Rights, 25*(9), 1625-1657. <https://doi.org/10.1080/13642987.2020.1859484> | Continuum | Study draws on the Records Continuum Model (Frank et al.). “Continuum-based conceptual understandings of records and recordkeeping” | 1 | The study is informed by the theory | Children-in-care’s records should be kept perpetually and be available to care-leavers. “Recordkeeping [is] part of the process by which children construct their worldview and social bonds” |
| Adley, N., & Jupp Kina, V. (2017). Getting behind the closed door of care leavers: understanding the role of emotional support for young people leaving care. *Child & Family Social Work, 22*(1), 97-105. <https://doi.org/10.1111/cfs.12203> | Convoy model | “The convoy model is a developmental systems model grounded in theories of attachment and social roles. It defines a relational or social network as a dynamically evolving protective structure incorporating all relationships perceived to be close and important to the individual, beginning with attachment figures in infancy and expanding to include other important people as the individual develops. Just as the attachment figure provides a secure base from which the infant can explore the world, the convoy provides the same secure base from which to explore the world as the person ages” (Antonucci et al., 2011). “Within this paper, we will explore the base from which care-leavers explore the world through identifying their perspectives on their own emotional support networks and the role that this network has played within their experience of professional practice.” | 3 | Mentioned up front as the guiding theory, but not mentioned at all after that. | While not explicitly linked to the theory, authors recommend working to identify potentially supportive people in the child’s social ecology well before leaving care, to develop a more robust support network. |
| Bennwik, I.-H. B., Oterholm, I., & Kelly, B. (2023). ‘Disability is not a word we use’: Social workers’ professional judgements about support for disabled young people leaving care. *Child & Family Social Work, 28*(2), 443-453. <https://doi.org/10.1111/cfs.12975> | Critical disability | Critical disability studies or theory (Goodley) emphasise intersectionality, which in this context includes care-leavers and disability, as well as the distinctions between disabled and able-bodied, impaired and disabled, etc. | 1 | Study was informed and shaped by the theory | Social workers need to be educated in critical disability perspectives to enable a more empowering social service. This will strengthen the championing of disabled youths’ rights and increase service options. |
| Bennwik, I.-H. B., Oterholm, I., & Kelly, B. (2023). ‘My disability was my own responsibility’: An institutional ethnography of the transitional experiences of disabled young people leaving care. *Children and Youth Services Review, 146*, 106813. <https://doi.org/10.1016/j.childyouth.2023.106813> | Critical disability | Critical disability (Goodley) studies or theory emphasises intersectionality, which in this context includes care-leavers and disability, as well as the distinctions between disabled and able-bodied, impaired and disabled, etc. | 1 | Study was informed and shaped by the theory | Normative discourses about disability need to be questioned. The resistance and agency of disabled care-leavers should be supported. |
| Wilson, D. B. (2020). Youth in transition and foster care alumni as empowered consumers. *Journal of Family Social Work, 23*(5), 411-430. <https://doi.org/10.1080/10522158.2019.1681335> | Critical social theory of youth empowerment | This study is guided by the Critical Social Theory of Youth Empowerment (Jennings et al.), which highlights young people working together for social and political change. “At the individual level, critical youth empowerment is meant to build capacity, and at the collective level, strengthen community partnership through organizations and communities, and enhance their mutual quality of life.” (p. 7) | 2 | Study is based on this theory. | Authors recommend that social work researchers should seek the input of the youth in transition and foster care alumni as one of their evaluation tools in measuring program effectiveness and that the perceptions of youth in transition and foster care alumni should be represented as an accepted practice in social work research and be integrated into child welfare policy and program development. |
| Tobolowsky, B. F., Scannapieco, M., Aguiniga, D. M., & Madden, E. E. (2019). Former foster youth experiences with higher education: Opportunities and challenges. *Children and Youth Services Review, 104*, 104362. <https://doi.org/https://doi.org/10.1016/j.childyouth.2019.05.039> | Cultural capital | The study explores the views of foster alumni, caseworkers, community partners, and foster parents regarding the challenges and supports that help or hamper the alumni’s postsecondary success. Cultural capital theory (Bourdieu) suggests that cultural capital is gained through a socialization process over time and is discussed in the findings section. | 2 | Mentioned and findings linked to some of the theory. | Authors recommend support while alumni are still in care, and a college support programme that links foster alumni with mentors on campus. |
| Mendes, P., Saunders, B., & Baidawi, S. (2016). Indigenous young people transitioning from out-of-home care (OOHC) in Victoria, Australia: The perspectives of workers in indigenous-specific and non-indigenous non-government services. *International Indigenous Policy Journal, 7*(3). <https://doi.org/10.18584/iipj.2016.7.3.2> | Cultural connectedness | Study draws on the notion of ‘cultural connectedness’ (Carrière & Richardson) which enables culturally appropriate ways of working. Study found that cultural connectedness and identity to be more important for indigenous care-leavers than attachment theory. The latter prioritises an individual relationship with any competent caregiver, while the former prioritises a relationship with one’s cultural heritage, community and group. | 2 | Study is not strongly driven by theory, but is informed by cultural constructs | Closer cooperation between state and indigenous children’s services, or rather care-leaving services lead by the indigenous services to ensure culturally appropriate and integrating service delivery to indigenous care-leavers. |
| Adeboye, T. K., Guerreiro, M. D., & Höjer, I. (2019). Unveiling the experiences of young people in foster care: Perspectives from Portugal and Nigeria. *International Social Work, 62*(1), 433-446. <https://doi.org/10.1177/0020872817731147> | Cumulative advantages and disadvantages | The theory of cumulative advantages and disadvantages (Singer & Berzin) “examines the heterogeneity principle with preparation, support and the effect of early transition to early or emerging adulthood among young people.” Past experiences of care-leavers shaped by biological family affiliation, cultural practices and causes of separation. | 1 | Theory informed data collection tool and an a priori model that was used for data analysis | No theory-informed practice recommendations provided. |
| van Breda, A. D., & Pinkerton, J. (2020). Raising African voices in the global dialogue on care-leaving and emerging adulthood. *Emerging Adulthood, 8*(1), 6–15. <https://doi.org/10.1177/2167696819870356> | Decoloniality | This article, coauthored by scholars from North and South, advocates for North-South dialogue, highlighting challenges like marginalizing African experience and scholarship, and divergent social concepts. Decoloniality (Ndlovu-Gatsheni) is used to point out the discrepancies between Global North and Global South in relation to care-leaving practice and scholarship. | 1 | The article is grounded in decolonial theory | No theory-informed practice recommendations provided. |
| Doucet, M., Pratt, H., Dzhenganin, M., & Read, J. (2022). Nothing about us without us: Using Participatory Action Research (PAR) and arts-based methods as empowerment and social justice tools in doing research with youth ‘aging out’ of care. *Child Abuse & Neglect, 130*, 105358. <https://doi.org/10.1016/j.chiabu.2021.105358> | Delayed adulthood | Adopting participatory action research and arts-based methods meant declining to use care-leaving theories that does not recognise the unique experiences of individuals or that boxes individuals. Delayed adulthood (Beaujot) describes people in quite negative terms (unstable, undecided, etc.) which defines young people as a social problem to be studied and controlled. | 3 | Term mentioned only once in the introduction. | The study rejected this theory, so no practical application beyond rejecting it. |
| Bogdanova, E. (2017). Russian SOS Children’s Villages and deinstitutionalisation reform: Balancing between institutional and family care. *Zhurnal Issledovanii Sotsialnoi Politiki [The Journal of Social Policy Studies], 15*(3), 395-406. <https://doi.org/10.17323/727-0634-2017-15-3-395-406> | Development | Developmental theory (Erikson) is used to show that SOS Children’s Villages avoid the pitfalls of institutional care, by maintaining family-like care settings. Removal from family and placement in care can negatively impact the child’s development. | 2 | Study is informed by theory, but its place is not central | Recommend that SOS Children’s Villages are aligned with this theory and should not be closed down or restructured under the deinstitutionalisation agenda |
| Dickens, L. (2018). One‐year outcomes of youth exiting a residential care facility in South Africa. *Child & Family Social Work, 23*(4), 558-565. <https://doi.org/10.1111/cfs.12411> | Developmental social welfare | A social development approach (Patel), also known a developmental social welfare, is necessary to provide a secure platform for care-leavers in adverse contexts: “job creation, income development projects and social entrepreneurship” (p. 564). Welfare pluralism is also important – no one group or organisation can do it on their own. | 3 | Theory comes in right near the end as part of recommendation | Recommend greater attention to employment, income, poverty reduction. Diverse partners (state, NGO, private sector, etc) need to work collaboratively. |
| Van Breda, A. D. (2016). The roles of agency and structure in facilitating the successful transition out of care and into independent living. *Social Work Practitioner-Researcher, 28*(1), 36-52. <https://doi.org/10.25159/2415-5829/1349> | Developmental social welfare | Agency and structure are shown to be closely interrelated. This relationship or interface between agency and structure has great value for South Africa, as it is aligned with developmental social welfare theory (Patel) that is also linked in the study. | 3 | Developmental social welfare theory is discussed in the analysis section in bringing together of the agency-structure discussion. | The author recommends that just as developmental social welfare stresses the need for both micro and macro interventions being integrated at all levels to address the needs of individual so too, we should not be debating agency versus structure, but rather working on both agency and structure and particularly the interactions between them. |
| Van Breda, A. D., & Dickens, L. F. (2017). The contribution of resilience to one-year independent living outcomes of care-leavers in South Africa. *Children and Youth Services Review, 83*, 264-273. <https://doi.org/10.1016/j.childyouth.2017.11.009> | Developmental social welfare | This article draws on data from the only longitudinal study on care-leaving in South Africa. The study shows that both micro and macro resilience processes feature equally prominently. The South Africa’s developmental social welfare theory (Patel) is drawn on to argue that a developmental welfare approach addresses both the macro structural processes that facilitate social well-being and micro interventions to facilitate social well-being. However, South African young people who make the transition are further disadvantaged due to South Africa’s current macro status. | 3 | Mentioned once in discussion section. | Authors recommends the importance of helping young people preparing to leave care to establish connections back home so that by the time they upon transition the young person has connections and an established support network. The broader welfare issues and macro policy and structural interventions to address wide scale social inequality needs to be addressed. |
| Houston, S. (2023). Dialectical critical realism, transformative change and social work. *Critical and Radical Social Work, 11*(1), 27-41. <https://doi.org/10.1332/204986021X16455451510551> | Dialectical critical realism | Dialectical critical realism (Bhaskar) accepts the external reality of the world, separate from the researcher, and accounts for emancipatory change in the social work, thus accommodating anti-oppressive practice. This theory is applied to a fictious case example of a care-leaver. | 1 | This is a theoretical paper, using care-leaving as a case example to illustrate theory | Although no practice recommendations are made, the application of dialectical critical realism is set out in a detailed (fictitious) case example, to illustrate its practical application. |
| Nsonwu, M. B., Dennison, S., & Long, J. (2015). Foster care chronicles: Use of the arts for teens aging out of the foster care system. *Journal of Creativity in Mental Health, 10*(1), 18-33. <https://doi.org/10.1080/15401383.2014.935546> | Drama therapy | The authors construct drama therapy (an intervention) as a theory (Carta et al.), based on the how stories and the arts operate to shift people’s experiences and perceptions of the world. Participants reported improved self-image, self-healing and self-efficacy. | 1 | Was a practice model. | SW professionals need to develop the ability to engage with their clients by utilizing innovative and nontraditional forms of expression via the creative arts. |
| Carey, C. (2023). Coping with stigma: Experiences and responses of former youth in care. *Qualitative Sociology Review, 19*(3), 26-51. <https://doi.org/10.18778/1733-8077.19.3.02> | Dramaturgical approach | Goffman’s theory of the dramaturgical approach is interested in how the self is presented to others and impressions are managed. Stigma involves the discrediting of the self. Goffman argues that those who are stigmatised have agency and can push back. This theory was used to frame care-experienced people’s experiences of stigma. | 1 | Goffman’s theory informs the entire study | No theory-informed practice recommendations provided. |
| Refaeli, T., Zeira, A., & Benbenishty, R. (2022). Ten years later: Satisfaction with intimate relationships and parenthood of young people aging out of residential care. *Child Abuse & Neglect, 129*, 105635. <https://doi.org/10.1016/j.chiabu.2022.105635> | Dyadic responses to trauma theory | The study explored various factors associated with satisfaction with both intimate relationships and parenthood a decade after leaving care. The dyadic responses to trauma theory (Marshall & Kuijer) suggests that traumatic life events can impact intimate relationships, and this study investigates the relationship between traumatic events and satisfaction with relationships. The study reveals that traumatic life events, both before and after age 18, significantly impact satisfaction with intimate relationships, aligning with dyadic responses to trauma theory. | 2 | Mentioned in discussion to illuminate findings | Psychological interventions are recommended to enhance care-leavers’ well-being as intimate partners, addressing traumatic life events, mental distress, and substance abuse, while also boosting their personal resources like self-esteem. |
| Couvrette, A., & Lanctôt, N. (2017). Between parental efficacy and parental distress: How early maladaptive schemas contribute to the motherhood experience of young mothers who have aged out of care. *Adoption & Fostering, 41*(2), 170-187. <https://doi.org/10.1177/0308575916662162> | Early maladaptive schemas | Early maladaptive schemas are “a broad, pervasive theme or pattern, comprised of memories, emotions, cognitions and bodily sensations, regarding oneself and one’s relationship with others, developed during childhood or adolescence, elaborated throughout one’s lifetime and dysfunctional to a significant degree” (p. 173) or “filters through which people perceive and understand their reality”. This study aimed to see how the 15 Early Maladaptive Schemas (Young) were related to two aspects of parenting – efficacy and distress – among young care-leaver mothers. | 1 | Study is driven by theory | Psychosocial interventions are needed to deal with negative Early Maladaptive Schemas, to reduce parental distress and improve efficacy. |
| Dutta, S. (2017). Experiences of young Indian girls transiting out of residential care homes. *Asian Social Work and Policy Review, 11*(1), 16-29. <https://doi.org/10.1111/aswp.12107> | Ecological | Used the micro (access to services), meso (social support and care home characteristics) and macro (societal factors and role of state) systems of ecological theory (Bronfenbrenner) to understand CL. The individual experience of care-leaving is influenced by an interplay between these levels and the individual care-leaver. | 2 | Theory used to frame findings | A supportive social environment is necessary for successful care-leaving, with a key focus on aftercare. |
| Dutta, S. (2018). Preparation for social reintegration among young girls in residential care in India. *International Journal of Child, Youth & Family Studies, 9*(2), 151-170. <https://doi.org/10.18357/ijcyfs92201818217> | Ecological | Used micro (individua), meso (care home and support systems) and macro (state) levels of ecological theory (Bronfenbrenner) to frame discussion of findings. | 3 | Theory used briefly towards the end to summarise | No theory-informed practice recommendations provided. |
| Fernandez, E., Delfabbro, P., Ramia, I., & Kovacs, S. (2019). Children returning from care: The challenging circumstances of parents in poverty. *Children and Youth Services Review, 97*, 100-111. <https://doi.org/10.1016/j.childyouth.2017.06.008> | Ecological | Ecological theory (Bronfenbrenner) is used to explain child maltreatment and then to explain the factors influencing family reunification. Ecological factors of disadvantage and poverty are argued to influence coming into care and reunification. These factors included housing, finances, substance abuse, domestic violence, disability, and indigeneity/race. | 1 | Study seeks factors in the social environment | Practice needs to attend to the social ecologies of families, not just to the child or to the family, to reduce children coming into care and increase family reunification. |
| Harder, A. T., Mann-Feder, V., Oterholm, I., & Refaeli, T. (2020). Supporting transitions to adulthood for youth leaving care: Consensus based principles. *Children and Youth Services Review, 116*, 105260. <https://doi.org/10.1016/j.childyouth.2020.105260> | Ecological | Interventions to support care-leavers can be set out on a micro-meso-macro continuum, using social ecological theory (Bronfenbrenner). Care-leavers interact with multiple systems at multiple levels. | 2 | Theory used post-hoc to explain findings | Alignment of practice is needed at policy, organisational, operational and direct intervention levels. |
| Harwick, R. M., Unruh, D., & Lindstrom, L. (2020). Transition to adulthood for youth with disabilities who experienced foster care: An ecological approach. *Child Abuse & Neglect, 99*, 104310. <https://doi.org/10.1016/j.chiabu.2019.104310> | Ecological | Generated an ecological framework (Bronfenbrenner) of barriers that need to be addressed to enable the progression of disabled care-leavers, viz. “high school graduation requirements, high quality Independent Living Programs, positive relationships and highly skilled professionals; frequent placement changes; and inappropriate disability or mental health diagnosis” (p. 1). The ecological theory emerged during data analysis as a useful way to frame the findings. Focus on exo, meso and micro systems. | 2 | Ecological came in after the data had been collected, and informed their interpretation | Greater attention to be given to the social environment of care-leavers with disabilities. |
| January, S.-A. A., Trout, A. L., Jacqueline, H.-D. A., Duppong Hurley, K. L., & Thompson, R. W. (2018). Perspectives on factors impacting youth’s reentry into residential care: An exploratory study. *Journal of Child and Family Studies, 27*(8), 2584-2595. <https://doi.org/10.1007/s10826-018-1093-5> | Ecological | Ecological systems theory (Bronfenbrenner) was used to understand the factors that result in the re-entry of care-leavers back into residential care. Therefore, interviews with both care-leavers and their caregivers, regarding various domains of the social ecology (e.g., school, family, relationships). Proximal systems (unhealth relationships) seem most prominent in leading care-leavers back into care. | 1 | Study design driven by theory | Because the study was exploratory, no recommendations for practice were made. |
| Modi, K., Prasad, A., & Mishra, J. (2018). Assisting youth leaving care: Understanding Udayan Care’s aftercare programme through the prism of ecological systems theory. *Scottish Journal of Residential Child Care, 17*(1), 1-24. <https://doi.org/10.17868/strath.00084531> | Ecological | Study developed an ecological framework, drawing on Bronfenbrenner’s levels, for care-leavers. Outcomes in various domains (e.g., education and health) are mapped onto the framework. | 1 | Study driven by the theory | No theory-informed practice recommendations provided. |
| Pinkerton, J. (2021). Exploring history in the social ecology of care leaving: Northern Ireland as illustration. *Child & Family Social Work, 26*(2), 270-279. <https://doi.org/10.1111/cfs.12804> | Ecological | This study explores care-leaving from Bronfenbrenner’s social ecological modelling of human development which provides a promising conceptual framework-micro to macro layered systemic perspective but also draws attention to the ‘chronosystem’, covering both biographical and historical changes. The use of the historical dimension to Bronfenbrenner’s modelling was illustrated by considering the development of care-leaving within Northern Ireland over a 50-year period (1968–2018). Successful or unsuccessful outcomes reflect the processes and structures, the social ecology, within which a care-experienced young person engages in the social construction of interdependence as they move into adulthood. | 1 | Study is based on this theory | Author recommends an acknowledgement of the history of care-leaving as part of social ecology provides a deeper understanding of the systemic dynamics of care-leaving. A historical understanding is needed to better inform practitioners and the young people they work alongside, service designers and programme managers, policy makers and politicians, and all those interested in improving the life chances of care-experienced young people. |
| Refaeli, T., Zeira, A., & Benbenishty, R. (2022). Ten years later: Satisfaction with intimate relationships and parenthood of young people aging out of residential care. *Child Abuse & Neglect, 129*, 105635. <https://doi.org/10.1016/j.chiabu.2022.105635> | Ecological | This study focused on Israeli care-leavers a decade after leaving care and explored various factors associated with satisfaction with both intimate relationships and parenthood. Using ecological theory (Bronfenbrenner, 2005), the factors that contribute to life satisfaction are identified. The theory emphasises how individuals grow and flourish in settings that are constantly changing as a result of interactions between various nested ecological contexts. | 1 | This theory served as the basis for the study. | All the systems encompassing the care-leavers’ backgrounds, the characteristics of the environment in which they grew up (including placement characteristics and traumatic life history) served as the microsystem. Personal characteristics (self-esteem, mental distress, and alcohol use) represented the ontogenic system. Care-leavers’ external environment, also part of the microsystem, was examined by informal support need to be fully understood to intervene. |
| Van Breda, A. D. (2016). The roles of agency and structure in facilitating the successful transition out of care and into independent living. *Social Work Practitioner-Researcher, 28*(1), 36-52. <https://doi.org/10.25159/2415-5829/1349> | Ecological | Agency and structure are closely interrelated, in the person-in-environment or ecological theory (Weiss-Gal). This relationship or interface between agency and structure has great value for South Africa, as it is aligned with ecological resilience theory that is also linked in the study. Young people interface with the environment. They are interactional or relational, reflecting the ways in which an individual identifies and mobilises resources in the social environment. | 2 | The ecological resilience theory in discussed in the analysis section in bringing together of the agency-structure discussion. | Care-leaving services should focus on both individual development of young people (agency) and the development of systems around them (structure) and the interactions between them (interface). Such holistic approaches are vital to facilitating successful transitions to independent living. |
| Bond, S. (2020). Care leavers’ and their care workers’ views of preparation and aftercare services in the Eastern Cape, South Africa. *Emerging Adulthood, 8*(1), 26–34. <https://doi.org/10.1177/2167696818801106> | Emerging adulthood | The tasks and challenges of Emerging Adulthood (Arnett) are discussed in the opening pages of the article. | 3 | Theory largely absent from methodology and findings until the just before the end | No theory-informed practice recommendations provided. |
| Bond, S., & van Breda, A. D. (2018). Interaction between possible selves and the resilience of care-leavers in South Africa. *Children and Youth Services Review, 94*, 88-95. <https://doi.org/10.1016/j.childyouth.2018.09.014> | Emerging adulthood | Emerging Adulthood (Arnett) is an in-between stage, linked to the transition between being in care and after leaving care – thus Emerging Adulthood is the transition out of care. | 2 | Study has emerging adulthood as a foundation, but it is not much used to discuss findings | No theory-informed practice recommendations provided. |
| Dickens, L. F., & Marx, P. (2020). NEET as an outcome for care leavers in South Africa: The case of Girls and Boys Town. *Emerging Adulthood, 8*(1), 64-72. <https://doi.org/10.1177/2167696818805891> | Emerging adulthood | Emerging adulthood (Arnett) reflects a nonlinear journey towards greater independence, with NEET being one of the retrograde parts of the journey. Care-leavers have to navigate this with little support and recourse. | 3 | Theory mentioned earlier and later, but does not shape the collection or analysis of data | Minimum standards or criteria should be set as required prior to disengagement from care, to increase chances of successful transition from care into anon-NEET status. |
| Doucet, M., Pratt, H., Dzhenganin, M., & Read, J. (2022). Nothing about us without us: Using Participatory Action Research (PAR) and arts-based methods as empowerment and social justice tools in doing research with youth ‘aging out’ of care. *Child Abuse & Neglect, 130*, 105358. <https://doi.org/10.1016/j.chiabu.2021.105358> | Emerging adulthood | Adopting participatory action research and arts-based methods meant declining to use care-leaving theories that does not recognise the unique experiences of individuals or that boxes individuals. Emerging adulthood (Arnett) describes people in quite negative terms (unstable, undecided, etc.) which defines young people as a social problem to be studied and controlled. | 3 |  | The study rejected this theory, so no practical application beyond rejecting it. |
| Dziro, C. (2020). Challenges and opportunities experienced by young adults transitioning out of informal kinship-based foster care in Bikita District, Zimbabwe. *Emerging Adulthood, 8*(1), 82–91. <https://doi.org/10.1177/2167696819870019> | Emerging adulthood | Emerging adulthood (Arnett) was used to refer to an age group, not as a theoretical construct or developmental stage. | 3 | Theory is mentioned but not used in the study | No theory-informed practice recommendations provided. |
| Gypen, L., Stas, L., West, D., Van Holen, F., & Vanderfaeillie, J. (2023). Longitudinal outcomes of employment, income and housing for Flemish care leavers. *Developmental Child Welfare, 5*(1), 36-58. <https://doi.org/10.1177/25161032231157094> | Emerging adulthood | Most care-leaving outcomes improve over time, supporting emerging adulthood theory (Dixon & Stein). Emerging adulthood explains a period of instability in early adulthood, that settles over time, as normative. | 2 | Theory mentioned only once in the discussion | No theory-informed practice recommendations provided. |
| Klodnick, V. V., & Samuels, G. M. (2020). Building home on a fault line: Aging out of child welfare with a serious mental health diagnosis. *Child & Family Social Work, 25*(3), 704-713. <https://doi.org/10.1111/cfs.12747> | Emerging adulthood | This study explains how and why homelessness occurs among youth with serious mental health struggles after aging out of residential and transitional living programmes. Emerging adulthood (Arnett) was mentioned when discussing the challenges of youth transitions, but because of the accelerated transition to adulthood of care-leavers, authors see emerging adulthood theory as of limited relevance to care-leaving. | 3 | Mentions the theory twice in literature review the study. | No theory-informed practice recommendations provided. |
| Pryce, J. M., Jones, S. L., Wildman, A., Thomas, A., Okrzesik, K., & Kaufka-Walts, K. (2016). Aging out of care in Ethiopia: Challenges and implications facing orphans and vulnerable youth. *Emerging Adulthood, 4*(2), 119-130. <https://doi.org/10.1177/2167696815599095> | Emerging adulthood | This article seeks to develop an understanding of the complex dynamics of navigating emerging adulthood (Arnett) following transition from institutional care in Ethiopia. The theory of emerging adulthood is used to explore this developmental period across cultures “through focus on the universal task of transitioning to the achievement of adult competencies in the developmental domains of career, family, and personal development” (p. 119). | 3 | Mentioned in literature review and once in findings. | Authors recommend the need for more transitional services (i.e., employment experience and housing assistance), as those young people who had employment experience and funding for housing reported more success in their transition. “Experiential training in life skills, financial literacy, and counseling or support with coping, and building a bridge between life in and out of the CYCC” (p. 128). |
| Sulimani-Aidan, Y. (2017a). Future expectations as a source of resilience among young people leaving care. *British Journal of Social Work, 47*(4), 1111–1127. <https://doi.org/10.1093/bjsw/bcw077> | Emerging adulthood | During emerging adulthood (Arnett), young people’s hopes for the future are especially important and could influence their goal setting and motivation to achieve those goals and that this period is characterized by uncertainty amongst care-leavers. Planning for the future is very important in emerging adulthood this transitional period. The goal of this study was to address the role of future expectations among young people leaving care in the context of resilience theory and emerging adulthood theory. | 1 | The entire study is based on the emerging adulthood theory | Author recommends “an integrated intervention approach that will increase care-leavers’ future expectations should include the following: (i) their interests; (ii) perceived abilities; (iii) individual characteristics; and (iv) opportunities available to them and together with this the building of social networks” (p. 1121). |
| Sulimani-Aidan, Y. (2017c). To dream the impossible dream: Care leavers’ challenges and barriers in pursuing their future expectations and goals. *Children and Youth Services Review, 81*, 332-339. <https://doi.org/10.1016/j.childyouth.2017.08.025> | Emerging adulthood | This study relies on the emerging adulthood theory (Arnett), that characterizes exploration and future possibilities as one of the dominant features of young adults transitioning out of the care system. | 1 | Study is based on the emerging adulthood theory. Findings are based on the theory. | The author recommends that with the understanding of the specific challenges and barriers in those periods (e.g., immediately after leaving care, in the middle of their twenties), policy makers and professionals need to be informed about the resources and types of support care-leavers need during the different stages in their journey to adulthood. |
| Sulimani-Aidan, Y. (2018a). Assets and pathways in achieving future goals of residential care alumni. *Children and Youth Services Review, 89*, 71-76. <https://doi.org/10.1016/j.childyouth.2018.04.023> | Emerging adulthood | This study investigated the challenges and obstacles faced by 25 Israeli young adults who left care, while trying to realize their expectations and pursue their future plans. The study drew on the emerging adulthood theory (Arnett) which looked at the connection between the challenges care-leavers struggle with in their transition to independent living, their future outlook, and their chosen paths. | 1 | Emerging adulthood was the basis of the study and was drawn on in the analysis of the findings. | All care-leavers need support and follow-up aftercare services, which they do not receive. This calls for an integrative and holistic approach in interventions. |
| Sulimani-Aidan, Y. (2019). Qualitative exploration of supporting figures in the lives of emerging adults who left care compared with their noncare-leaving peers. *Child & Family Social Work, 24*(2), 247-255. <https://doi.org/10.1111/cfs.12609> | Emerging adulthood | The study leans on the theory of emerging adulthood (Arnett) by exploring the informal and formal social networks of care-leavers compared with their non-care‐leaving peers during emerging adulthood. | 1 | Literature and analysis were fully based on the theory of emerging adulthood | Author recommends that professionals need to work to connect young people to new supportive figures such as professionals or mentors in their community. This can be done through “mentoring programmes or supervision apartments, and introduce them to new supportive connections that are available to them and are knowledgeable regarding the instrumental and developmental tasks of this period” (p. 254). |
| Sulimani-Aidan, Y. (2020a). Challenges in the transition to adulthood of young-adult Arabs who graduated from residential facilities in Israel. *Children and Youth Services Review, 113*, 104967. <https://doi.org/10.1016/j.childyouth.2020.104967> | Emerging adulthood | The study interviewed 50 young adults and their social workers regarding their views on the role of formal and informal social networks when leaving care, in the context of emerging adulthood theory (Arnett). | 1 | The emerging adulthood theory was used as in the literature review and the theoretical thematic analysis stage of the study. | The author recommends “connecting young people with new supportive frameworks where they could strengthen their social ties, build positive and supportive relationships that would expose them to new opportunities, and find others who could advise them when needed” (p. 6). |
| Sulimani-Aidan, Y. (2020b). Social networks during the transition to adulthood from the perspective of Israeli care leavers and their social workers. *Children and Youth Services Review, 115*, 105075. <https://doi.org/10.1016/j.childyouth.2020.105075> | Emerging adulthood | The study interviewed 50 young adults and their social workers regarding their views on the role of formal and informal social networks when leaving care, in the context of emerging adulthood theory (Arnett). | 1 | The emerging adulthood theory was used as in the literature review and the theoretical thematic analysis stage of the study. | Even limited formal and informal support to emerging adults leaving care, can be helpful. This enables them to cope better with emotional and practical challenges typical of this age group. Emerging adults must develop greater independence as part of this life stage, but this is done within relational contexts. |
| Sulimani-Aidan, Y., & Melkman, E. (2018). Risk and resilience in the transition to adulthood from the point of view of care leavers and caseworkers. *Children and Youth Services Review, 88*, 135-140. <https://doi.org/10.1016/j.childyouth.2018.03.012> | Emerging adulthood | This study addresses the challenges and resources in transition to adulthood from the viewpoint of 50 care-leavers and caseworkers in Israel. The theory of emerging adulthood and its aspects are discussed as per the transitional period e.g., resilience can emerge during and following emerging adulthood. | 3 | Theory is used throughout the study drawing on its findings to link to the theory. | The authors recommend introducing programmes integrating more components that connect care-leavers with other young adults while strengthening their existing friendships to prevent isolation. Preparation programs need to help youth become more aware of their rights as care-leavers and encourage them to realize them once they leave care. |
| Sundly, A., Keating, M., Effiong, A., & Saif, A. O. (2022). Neuroscientific evidence and care leaving: a multidisciplinary critical commentary. *International Journal of Child, Youth & Family Studies, 14*(1), 30-46. <https://doi.org/10.18357/ijcyfs141202321283> | Emerging adulthood | Emerging adulthood theory (Arnett) draws attention to the ongoing brain development of adolescents and young adults (which does not cease in childhood). | 1 | Entire study is based on the theory | While investment in early childhood development is important, based on neurodevelopment at that age, investment in emerging adults is similarly important, based on neurodevelopment at that age. |
| Takele, A. M., & Kotecho, M. G. (2020). Female care-leavers’ experiences of aftercare in Ethiopia. *Emerging Adulthood, 8*(1), 73-81. <https://doi.org/10.1177/2167696819868355> | Emerging adulthood | This study sheds light on the issue of aftercare support for female emerging adults who are transitioning from care to adulthood in Ethiopia*.* The emerging adulthood stage is looked at specifically in relation to Ethiopian practice. | 3 | The theory is mentioned once in the introduction and discussion. But limited reference to the theory. | Authors recommend integrating the needs and profiles of Ethiopian care-leavers (as part of a larger population of emerging adults) into schools’ curricula is greatly required and a need to change the institutions’ caregiving practice of female care-leavers in a way that addresses their holistic developmental needs. “Social workers have a role in advocating for aftercare support, which should be incorporated in the institution’s rehabilitation scheme to make care-leavers’ transition to emerging adulthood successful” (p. 80). |
| Tyrell, F. A., & Yates, T. M. (2018). Emancipated foster youth’s experiences and perceptions of the transition to adulthood. *Journal of Youth Studies, 21*(8), 1011-1028. <https://doi.org/10.1080/13676261.2018.1441983> | Emerging adulthood | Emerging adulthood theory (Arnett) was used as the analytic frameworks. They study aimed to evaluate the applicability of emerging adulthood to the experience of transition-aged foster youth, it offered an opportunity to identify important themes as they emerged with young adults exiting care system. While some aspects of care-leavers matched emerging adulthood themes, others did not, such as self-focus and optimism, exploration and feeling in-between. | 1 | Entire study is based on the theory of emerging adulthood. | Authors recommend intervention efforts to reduce homelessness and delay pregnancy among foster youth, and programs that provide social and material resources, which may promote youth’s educational attainment and employment opportunities. |
| van Breda, A. D., & Pinkerton, J. (2020). Raising African voices in the global dialogue on care-leaving and emerging adulthood. *Emerging Adulthood, 8*(1), 6–15. <https://doi.org/10.1177/2167696819870356> | Emerging adulthood | This article, coauthored by scholars from North and South, advocates for North-South dialogue, highlighting challenges like marginalizing African experience and scholarship, and divergent social concepts. The topic of emerging adulthood (Arnett) is a theory used to structure the findings and discussion. | 2 | Findings are based on this theory | No theory-informed practice recommendations provided. |
| van Breda, A. D., Munro, E. R., Gilligan, R., Anghel, R., Harder, A., Incarnato, M., Mann-Feder, V., Refaeli, T., Stohler, R., & Storø, J. (2020). Extended care: Global dialogue on policy, practice and research. *Children and Youth Services Review, 119*, 105596. <https://doi.org/10.1016/j.childyouth.2020.105596> | Emerging adulthood | The article examines extended care policies, practices, and research using a common matrix in over 10 countries. No focus on theory in the entire article until the recommendation. | 3 | Only mentions the theory and only in the conclusion section | No theory-informed practice recommendations provided. |
| Zeira, A., Refaeli, T., Achdut, N., & Benbenishty, R. (2023). Economic self-sufficiency and the employment outcomes of care leavers: A 10-year follow up. *Child & Family Social Work, 28*(3), 635-645. <https://doi.org/10.1111/cfs.12990> | Emerging adulthood | The developmental perspective of emerging adulthood (Arnett) is incorporated into the multidisciplinary life course perspective. Thus, emerging adulthood is not seen a theory but as a concept. | 3 | Mentioned four times in the article and not explained theoretically | No theory-informed practice recommendations provided. |
| Kennedy, T. D., Flach, Y., Detullio, D., Millen, D. H., Englebert, N., & Edmonds, W. A. (2019). Exploring emotional intelligence and IQ as predictors of success of foster care alumni. *Journal of Child and Family Studies, 28*(12), 3286-3295. <https://doi.org/10.1007/s10826-019-01503-8> | Emotional intelligence | Emotional intelligence (Bar-On), but not general intelligence, contributed to improved care-leaving outcomes. | 1 | Student design informed by theory | No theory-informed practice recommendations provided. |
| Østergaard, J. (2023). Misfitting feelings: Young care leavers’ emotional work during the transition to adulthood. *Child Care in Practice, 29*(3), 278-296. <https://doi.org/10.1080/13575279.2023.2167809> | Emotional-management model | This article examines the emotional work that young adult care-leavers perform during their transition to adulthood. To understand what young adults who have been placed in care think and do about their feelings in relation to their birth parents, the study draws on Hochschild’s emotional-management model of “deep acting” and “surface acting”. | 1 | Theory is used as a conceptual base for the entire study | Managing feelings leaves the young adults in moments of pinch or discrepancy that they must act on to successfully transition to adulthood. Social policy needs to address this when young people have aged out of care. |
| Batista, T., Johnson, A., & Friedmann, L. B. (2018). The effects of youth empowerment programs on the psychological empowerment of young people aging out of foster care. *Journal of the Society for Social Work & Research, 9*(4), 531-549. <https://doi.org/10.1086/700275> | Empowerment | Youth empowerment (Havlicek et al.) links or overlaps with youth development, youth power, youth voice, youth participation, youth engagement, youth agency, youth governance, and youth organizing. Youth empowerment to participate is thought to improve care-leaving outcomes. This study examined the effect of participating in youth empowerment programmes on psychological empowerment, which concerns “increased control over one’s life circumstances and/or influence in one’s relevant life domains”. Findings were positive: participation in youth empowerment programmes does increase the four domains of psychological empowerment. | 1 | A theory was empirically tested using quantitative methods | Care-leavers’ participation in youth empowerment programmes is recommended, though some cautions are expressed about possible negative effects. |
| Paul-Ward, A., & Lambdin-Pattavina, C. A. (2016). New roles for occupational therapy to promote independence among youth aging out of foster care. *The American Journal of Occupational Therapy, 70*(3), 1-5. <https://doi.org/10.5014/ajot.2016.017426> | Empowerment | Empowerment (not cited) is described as “the process of giving a voice and opportunities for participation to people regarding the decisions that affect their lives, is an important concept related to occupational justice. This process emphasizes a person’s responsibility to control his or her own life and resources. Therefore, the use of an empowerment approach that moves the development of independent living, vocational, and health maintenance skills out of didactic classroom settings into the community, providing opportunities for experiential learning for real-life problem solving, is crucial to promoting successful transitions.” (p. 2) | 2 | Theory appears only in the section corresponding roughly to implications for professional practice. | Study calls for the profession to assist young people transitioning out of care. Not much else is mentioned. |
| Bromark, K., Knutsson, O., & Spånberger Weitz, Y. (2023). Co-designing a dynamic tool to enhance participation for young people: A participatory design project with young service users and social workers. *Children and Youth Services Review, 147*, 106856. <https://doi.org/10.1016/j.childyouth.2023.106856> | Epistemic injustice | The study aimed to co-design processes and tools for increased participation for young people. Epistemic injustices (Fricker) can limit the participation and voice of young people. A series of participatory workshops were conducted to explore these themes with service users (including care-leavers). | 1 | The study is strongly informed by its theoretical frame | No theory-informed practice recommendations provided. |
| Benaton, T., Bowers-Brown, T., Dodsley, T., Manning-Jones, A., Murden, J., Nunn, A., & Community, T. P. O. (2020). Reconciling care and justice in contesting social harm through performance and arts practice with looked after children and care leavers. *Children & Society, 34*(5), 337-353. <https://doi.org/10.1111/chso.12370> | Ethic of care | The feminist ethics of care (Tronto) informed ethics and justice in how we work with children in and leaving care. The ethics of care emphasises interdependence with others – relationships of care. | 1 | The ethic of care informs an intervention | The feminist ethic of care can help young people to navigate through adversity, and can also help to challenge oppressive social structures and systems. |
| Uptin, J., & Hartung, C. (2023). Living in a crowd: Young people’s counter-narratives of care in a Thai orphanage. *Children’s Geographies, 21*(2), 257-270. <https://doi.org/10.1080/14733285.2022.2033954> | Ethic of care | This study examines the narratives of young people who grew up in an orphanage in Thailand. An ethic of care (Tronto), drawing from feminist scholarship, emphasises multiple levels of ethics, society and care. Care in these narratives is relational, shifting, and shaped by intersecting contextual factors which challenge a narrow reading of care as a transactional process. | 1 | Study is informed by conceptualisation of care, though an ethic of care mentioned only once. | No theory-informed practice recommendations provided. |
| Törrönen, M., Munn-Giddings, C., & Vornanen, R. (2023). Existential well-being among young people leaving care: Self-feeling, self-realisation, and belonging. *Ethics & Social Welfare, 17*(3), 295-311. <https://doi.org/10.1080/17496535.2022.2140176> | Existential well-being | This study explores young people’s perceptions of their existential well-being during the transition after leaving care. The theoretical framework of ‘existential well-being,’ which is a relational approach, is used. Three concepts of this theory are self-feeling, self-realisation and belonging. It offers a new theoretical focus to assist in understanding the life experiences of young people during their out-of-home care and their transition to independent life. | 1 | Entire study is based on theory | Further support is required for young people’s psychosocial and mental health in child welfare policy and practice. Ongoing support and a focus on interdependence are required. Being independent does not mean not needing support. |
| Goldfarb, K. E. (2016). ‘Self-responsibility’ and the politics of chance: Theorizing the experience of Japanese child welfare. *Japanese Studies, 36*(2), 173-189. <https://doi.org/10.1080/10371397.2016.1208531> | Experience | A theory of ‘experience’ (Hume) suggests that what we learn from past experiences is replicated in the future. In Japan, child welfare breaks this theory, by providing children with experience of dependency and social marginalisation, and then expect them to care for themselves independently when they age out of care (at ages 15-20). | 2 | Experience is used to make sense of data. | No theory-informed practice recommendations provided. |
| Karki, S., Ryynänen, O.-P., Salokekkilä, P., & Häggman-Laitila, A. (2023). Bayesian analysis of the factors explaining the disruptive behaviour of care leavers: A retrospective document analysis. *Children and Youth Services Review, 155*, 107174. <https://doi.org/10.1016/j.childyouth.2023.107174> | Explanatory modelling | This paper is a theory building paper, using Bayesian analysis of data, with an explanatory focus (Rautiainen et al.). Specifically, the study aims to explain care-leavers’ disruptive behaviour. Thus, no specific theory informs the study, but the study generates a ‘theory’ of disruptive behaviour among care-leavers. Substance abuse and a psychiatric diagnosis negatively impact (increase) disruptive behaviour, even when mental health services are received. Completion of secondary education or vocational training has the positive effect of reducing disruptive behaviour. | 1 | The whole paper is directed towards theory building | Aftercare services should focus on reducing risky behaviours, particularly addictions. Care-leavers should be supported to complete their education and to engage in the labour market. Foster appears to be the most conducive type of alternative care for improved outcomes. |
| Boddy, J., McCarthy, J. R., Gillies, V., & Hooper, C.-A. (2019). Troubling meanings of “family” for young people who have been in care: From policy to lived experience. *Journal of Family Issues, 40*(16), 2239-2263. <https://doi.org/10.1177/0192513X18808564> | Family | The study does not draw on a specific *theory* of family (McCarthy), but rather problematises naïve normative constructions of ‘family’, arguing for greater complexity of the construct. | 1 | Study sets out to problematise the construction of ‘family’ | Recommends setting aside normal, uncritical assumptions about ‘family’ and adopting more diverse and include constructions of ‘family’. |
| Gwenzi, G. D. (2020). Constructing the meaning of “family” in the context of out-of-home care: An exploratory study on residential care leavers in Harare, Zimbabwe. *Emerging Adulthood, 8*(1), 54-63. <https://doi.org/10.1177/2167696818800846> | Family | The study does not draw on a specific *theory* of family, but aimed to problematise the construct ‘family’ for care-leavers in Zimbabwe (Kedrick)). Some care-leavers used ‘family’ in the traditional biological sense, but others focused on family practices and family display. | 1 | Study driven by a theoretical question of what constitutes ‘family’ | There is need for policy and practice to support care-leavers in reflecting on what constitutes ‘family’ for them, and to support them in finding or creating family that is meaningful to them. |
| Adeboye, T. K., Guerreiro, M. D., & Höjer, I. (2019). Unveiling the experiences of young people in foster care: Perspectives from Portugal and Nigeria. *International Social Work, 62*(1), 433-446. <https://doi.org/10.1177/0020872817731147> | Family practices approach | The article is not clear what the Family Practices Approach (Morgan) is, nor how it was used in this study, though it is mentioned several times throughout the article. | 3 | Theory mentioned up front, but not explained and no clear how it shaped the study | No theory-informed practice recommendations provided. |
| Sulimani-Aidan, Y. (2018b). Left behind: How to encourage biological parents’ involvement in their children’s group homes. *Child & Family Social Work, 23*(2), 180-187. <https://doi.org/https://doi.org/10.1111/cfs.12397> | Family systems | The study addresses the challenges and benefits of involving biological parents in homes in Israel and presents various means to encourage their involvement in care. The family systems theories (Minuchin) and the concept of co‐parenting is used to analyse the delicate and multifaceted relationship caseworkers and foster parents have with biological parents. | 2 | The literature section of the article discusses the challenges in working with biological parents using a family system theory perspective. | Author recommends involving birth parents within their child’s intervention programme while in care calls for changes both in policy and in the system. The agency’s attitudes as well as what policies and procedures are in place to train workers on how they can work collaboratively with biological parents is also important to changing current practice. |
| Font, S. A., Berger, L. M., Cancian, M., & Noyes, J. L. (2018). Permanency and the educational and economic attainment of former foster children in early adulthood. *American Sociological Review, 83*(4), 716-743. <https://doi.org/10.1177/0003122418781791> | Family theory | Authors argue that “prevailing family theory” (p. 737), which is not defined or cited, predicts that permanency placements will generate better care-leaving outcomes than remaining in foster care until aging out. The study finds that this is not borne out by the data. | 3 | Theory mentioned once, without citation, author, etc. | No theory-informed practice recommendations provided. |
| Berejena Mhongera, P. (2017). Preparing for successful transitions beyond institutional care in Zimbabwe: adolescent girls’ perspectives and programme needs. *Child Care in Practice, 23*(4), 372-388. <https://doi.org/10.1080/13575279.2016.1215291> | Feminism | Feminism (Delamont) is used to explain how female care-leavers’ experience of leaving care is different from men, due to the patriarchal patterns in society. Feminism is used to interpret how female participants construct a ‘successful transition’ out of care. Participants emphasized resources that would economically empower them (education, financial support and employment), particularly as women in a male-dominated society. | 1 | Study set out to understand the gendered experience of care-leaving | Care-leaving programmes need to address gender-specific needs of female care-leavers and promote girls’ access to assets and capabilities to improve livelihoods. Positive youth development programmes and mentoring are suggested. |
| Staines, J., Fitzpatrick, C., Shaw, J., & Hunter, K. (2023). ‘We need to tackle their well being first’: Understanding and supporting care-experienced girls in the youth justice system. *Youth Justice*, Advance online publication. <https://doi.org/10.1177/14732254231191977> | Feminist pathways | Drawing on feminist pathways theory (Belknap; Fedock & Covington), this article explores the reasons why girls may become involved in the youth justice system while in care, including how alleged offending behaviour may be an expression of previous trauma, feelings of not belonging or not being listened to. Feminist pathways highlight girls’ greater experiences of abuse and neglect, that can increase youth justice involvement. | 3 | Mentioned once as a gendered lens, but no explanation or later mention | No theory-informed practice recommendations provided. |
| Hollingworth, K., & Jackson, S. (2016). Falling off the ladder: Using focal theory to understand and improve the educational experiences of young people in transition from public care. *Journal of Adolescence, 52*, 146-153. <https://doi.org/10.1016/j.adolescence.2016.08.004> | Focal | Aims to see if focal theory (Coleman) can help explain the big differences in education outcomes between care-leavers and the general population. The study finds that challenges do not appear one at a time for care-leavers – from pre-care to post-care, challenges are multiple and substantial. This continues into the post-care educational phase contributing to poorer educational outcomes among care-leavers. | 1 | Theory informs study design and analysis | Extend care and increase opportunity for care-leavers to shape their own pathways, to pace themselves. Work to reduce the number of challenges faced at any one time. |
| Munro, E. R., & Simkiss, D. (2020). Transitions from care to adulthood: messages to inform practice. *Paediatrics and Child Health, 30*(5), 175-179. <https://doi.org/10.1016/j.paed.2020.02.007> | Focal | The care-leaving transition is often accelerated and compressed, not allowing care-leavers to focus on one challenge at a time, like most others their age. The numerous transitions that have to be managed at the same time – particularly transitions between service systems – can undermine the transition towards adulthood, as explained by focal theory (Coleman). | 2 | Study informed by theory | Transitions between services providers needs to be implemented more smoothly and caringly, so that care-leavers do not slip between the systems. Transitions should be gradual and aftercare support should be available. Service delivery should centre around the worker, to provide continuity to the care-leaver. |
| Mupaku, W. M., van Breda, A. D., & Kelly, B. (2021). Transitioning to adulthood from residential childcare during COVID-19: Experiences of young people with intellectual disabilities and/or autism spectrum disorder in South Africa. *British Journal of Learning Disabilities, 49*(3), 341-351. <https://doi.org/10.1111/bld.12409> | Focal | This study focuses on young people with intellectual disabilities and/or autism who, due to child welfare concerns, have grown up in children’s residential care and are now transitioning out of care at the age of 18 years towards young adulthood. Mentioned in the conclusion, focal theory (Coleman) could be used to assist in spreading the load of transitional demands over a longer time, in this way avoid over-burdening caregivers, this focus on caregivers helping care-leavers to continue to develop their learning and skills during lockdown should be closely supported by professionals. | 3 | Focal theory mentioned in the conclusion section of the article | The provision of online service provision to mitigate the challenges found and to strengthen and mobilise the positive opportunities participants report COVID-19 to have created. Psychosocial support services after leaving care should, thus, be considered an essential service for care-leavers with intellectual disabilities. Caregivers could be supported to deliver programmes via online individualised support from professionals and virtual tutorials or potentially with other caregivers. |
| van Breda, A. D., Munro, E. R., Gilligan, R., Anghel, R., Harder, A., Incarnato, M., Mann-Feder, V., Refaeli, T., Stohler, R., & Storø, J. (2020). Extended care: Global dialogue on policy, practice and research. *Children and Youth Services Review, 119*, 105596. <https://doi.org/10.1016/j.childyouth.2020.105596> | Focal | The article examines extended care policies, practices, and research using a common matrix in over 10 countries. Focal theory (Coleman) is mentioned in the recommendations. | 3 | Only mentions the theory and only in the conclusion section | No theory-informed practice recommendations provided. |
| Steenbakkers, A., van der Steen, S., Grietens, H., & Ellingsen, I. T. (2018). Do foster parents and care workers recognize the needs of youth in family foster care with a history of sexual abuse? *Journal of Child Sexual Abuse, 27*(7), 811-831. <https://doi.org/10.1080/10538712.2018.1520378> | Future orientation | This study does not state a theoretical lens, but data collection foregrounds a future orientation, mostly focused on self-actualization needs to be met with the support of both the foster and birth family. Some care workers recognised youth’s need to exercise agency in shaping their future, though this accompanied by an expectation that the youth should pursue their future goals independently. | 3 | The future orientation is not theorised and used only in the analysis section. | No theory-informed practice recommendations provided. |
| Sulimani-Aidan, Y. (2017a). Future expectations as a source of resilience among young people leaving care. *British Journal of Social Work, 47*(4), 1111–1127. <https://doi.org/10.1093/bjsw/bcw077> | Future orientation | Recognising that care-leaving is all about preparing for one’s future, this study explores the role of future expectations among care-leavers. The study finds that optimism and social support underpin future orientation. | 1 | The whole study is directed towards exploring future expectations | Personal and environment processes and resources need to be aligned to potentiate future expectations. |
| Sulimani-Aidan, Y. (2017c). To dream the impossible dream: Care leavers’ challenges and barriers in pursuing their future expectations and goals. *Children and Youth Services Review, 81*, 332-339. <https://doi.org/10.1016/j.childyouth.2017.08.025> | Future orientation | The challenges and barriers raised by the young adults in this study indicate struggle in each of the areas listed as a component of the future orientation theory (Nurmi). The study highlights future expectations, aspirations, hopes, possibilities, opportunities, plans, decisions, etc. | 1 | The future orientation cascades through the whole paper. | The authors propose an integrative and holistic approach in interventions to support young adults in their journey to independent adult life, in which they can chose their own paths and persist successfully towards realization of their future expectations and aspirations. |
| Van Audenhove, S., & Vander Laenen, F. (2017). Future expectations of young people leaving youth care in Flanders: the role of personal and social capital in coping with expected challenges. *Child & Family Social Work, 22*(1), 256-265. <https://doi.org/10.1111/cfs.12233> | Future orientation | The authors identified that there is a lack of prospective research in the field with the view of the implications of future orientation or perspectives (Schmidt). This study is part of an “ongoing follow-up study on Flemish youngsters on future perspectives [or orientation] on the verge of leaving youth care (first stage) and their experiences during and after the transition period to living independently”. | 1 | The study is based on the future perspective approach | Young people with positive future perspectives face challenges in societal acceptance, including stigma and lack of housing opportunities. Policy should focus on social inclusion and reintegration, considering individual’s position in society and life domains. |
| Van Audenhove, S., & Vander Laenen, F. (2017). Future expectations of young people leaving youth care in Flanders: the role of personal and social capital in coping with expected challenges. *Child & Family Social Work, 22*(1), 256-265. <https://doi.org/10.1111/cfs.12233> | Future perspectives | The authors identified that there is a lack of prospective research in the field with the view of the implications of future orientation or perspectives (Schmidt). This study is part of an “ongoing follow-up study on Flemish youngsters on future perspectives [or orientation] on the verge of leaving youth care (first stage) and their experiences during and after the transition period to living independently”. | 1 | The study is based on the future perspective approach | Young people with positive future perspectives face challenges in societal acceptance, including stigma and lack of housing opportunities. Policy should focus on social inclusion and reintegration, considering individual’s position in society and life domains. |
| Van Breda, A. D. (2020). Patterns of criminal activity among residential care-leavers in South Africa. *Children and Youth Services Review, 109*, 104706. <https://doi.org/10.1016/j.childyouth.2019.104706> | General strains | This paper reports on a longitudinal study of young people transitioning out of care and journeying towards young adulthood over a period of five years. Theoretical explanations for care-leavers transition into crime include general strains (Barn & Tan). The author states that the lack of bonding among care-leavers, for example, may leave them vulnerable to criminal activity | 3 | Mentioned once only in article in literature section. | Author recommends “preventive intervention to ‘crime-proof’ young people preparing to leave care and an option to extend care beyond age 18, who do engage in crime – even fairly minor criminal activity – should be referred into a diversion programme” (p. 9) |
| Kenny, K. (2023). The educational experiences of children in care across five decades: A new perspective on the education of looked after children in the UK. *Adoption & Fostering, 47*(1), 22–39. <https://doi.org/10.1177/03085759231157415> | Habitus | This study examined the educational experiences of looked after children by gathering their stories, as told in their own words. A theoretical framework was developed to analyse their stories: the concepts of reflexivity, agency and Bourdieu’s habitus were operationalised respectively as ‘thinking’ (investigating participants’ reflexivity with respect to both formal and informal educational experiences); ‘doing’ (capturing participants’ capacity to make informed decisions in service of their needs and despite constraints imposed on them from outside); and ‘being’ (understanding how participants’ perceptions of their own histories and development have contributed to their current sense of identity). With the help of this framework, the study was able to concentrate on the participants’ perceptions of their own education, offering a helpful starting point for a more comprehensive analysis of education. | 2 | Findings are presented according to theoretical constructs of thinking, being and doing | Findings support the implementation of a social pedagogic approach to working with looked after children by incorporating a more holistic educational journey. In practice this can be done by writing their own narratives, care-leavers could develop their agency over defining what sort of ‘education’ they want to experience and achieve the goals that they have set. |
| Steenbakkers, A., Ellingsen, I. T., Steffie van der, S., & Grietens, H. (2018). Psychosocial needs of children in foster care and the impact of sexual abuse. *Journal of Child and Family Studies, 27*(4), 1324-1335. <https://doi.org/10.1007/s10826-017-0970-7> | Hierarchy of needs | Drawing on Maslow’s hierarchy of needs, the study aims to assess the psychosocial needs of children in foster care according to their own views and to understand the influence of a self-reported history of sexual abuse on these needs. By incorporating all four psychological needs of Maslow’s hierarchy, the youth perspectives was covered in the analysis. | 1 | The entire study was based on the theory and analysis conducted by using the theory as a basis. | No theory-informed practice recommendations provided. |
| Steenbakkers, A., van der Steen, S., Grietens, H., & Ellingsen, I. T. (2018). Do foster parents and care workers recognize the needs of youth in family foster care with a history of sexual abuse? *Journal of Child Sexual Abuse, 27*(7), 811-831. <https://doi.org/10.1080/10538712.2018.1520378> | Hierarchy of needs | Drawing on Maslow’s hierarchy of needs, this study asks, “Do foster parents and care workers recognize the psychosocial needs of youth in family foster care with a history of sexual abuse?” (p. 814). This is important as listening to the voices of youth regarding their needs can positively influence their well-being. | 3 | Limited mention of the theory and drawing on in in the study. This was also because it was a follow-up from a previous study. | Practitioners should be sensitive to the needs of children and respond to them by assisting in meeting these needs. Foster parents should be helped in understanding the children’s needs. Children should participate in articulating their needs. |
| Klodnick, V. V., & Samuels, G. M. (2020). Building home on a fault line: Aging out of child welfare with a serious mental health diagnosis. *Child & Family Social Work, 25*(3), 704-713. <https://doi.org/10.1111/cfs.12747> | Home | The concept (not a theory as such) of ‘home’ is explored at length before the methodology. Youth in the study believed independence included the cultivation of a space that affirms one’s belonging, authenticity, competence as adult, security, and wellness—a place to call home. | 1 | Study directed by the concept of home | The concept of “home” and its impact on care-leavers lives during this major transition was found to be important aspect for future research. Cultivating this aspect of “home” is important but likely hidden and neglected in practice elements in preventing physical homelessness post care. |
| Gullo, F., García-Alba, L., Bravo, A., & del Valle, J. F. (2023). The psychosocial adjustment of care leavers in their transition to adult independent living (El ajuste psicosocial de jóvenes extutelados en su transición a la vida adulta independiente). *International Journal of Social Psychology, 38*(1), 35–65. <https://doi.org/10.1080/02134748.2022.2132747> | Homeostasis | The theory of subjective wellbeing homeostasis (Cummins) emerges to explain why subjective wellbeing tends to remain stable over time and context. This study aimed to analyse psychosocial adjustment of care-leavers along several measures, including subjective well-being. While self-esteem and psychological adjustment improved with age, well-being did not, explained by homeostasis theory. | 3 | Theory of homeostasis of subjective wellbeing emerges only once, briefly, in the discussion. | No theory-informed practice recommendations provided. |
| Refaeli, T., Benbenishty, R., & Zeira, A. (2019). Predictors of life satisfaction among care leavers: A mixed-method longitudinal study. *Children and Youth Services Review, 99*, 146-155. <https://doi.org/10.1016/j.childyouth.2019.01.044> | Homeostasis | The theory was mentioned in the findings section and links a finding from the study to the homeostasis theory (Cummins et al.). This finding was that life satisfaction of care-leavers one-year post-care is a strong predictor of life satisfaction three years later. The study states, “One explanation for this result derives from homeostasis theory, which claims that people generally tend to be stable in their reports of subjective well-being” (p. 152). | 3 | No other mention of theory except the once in findings that was mentioned. | One-on-one mentoring to assist in examining the individual perceptions that young people have of their life situation and learn from them what kind of changes in their lives are more meaningful for them. |
| Sulimani-Aidan, Y., Melkman, E., & Hellman, C. M. (2019). Nurturing the hope of youth in care: The contribution of mentoring. *American Journal of Orthopsychiatry, 89*(2), 134-143. <https://doi.org/10.1037/ort0000320> | Hope | This study aims to explore hopeful thinking (Synder) among youth in care and the contribution of mentoring relationships to the youths’ hope on the verge of leaving care while controlling for the youths’ various individual and placement characteristics. | 1 | Literature and analysis sections alludes to the hope theory. | Residential care “placements should seek to integrate both hope and mentoring as components in their intervention planning and staff training*.* Secondly, residential care placements to recruit mentors for longevity and encourage and train them to enable relationships that model independent living (autonomy) in order to enhance youths’ hope” (p. 141). |
| Modi, K., Kasana, S., Azam, A., & Madhavan, L. (2021). Education, poverty and social exclusion: Assessment of youth leaving care. *SN Social Sciences, 1*(4), 82. <https://doi.org/10.1007/s43545-021-00102-z> | Human Capability Approach | This study examines the interrelatedness of education, poverty and social exclusion among care-leavers through secondary literature and empirical data from Udayan Care’s national study. Analysing the findings from the lens of the human capability approach (Sen), the gaps in provisions of educational support to care-leavers and subsequent limitations to address challenges of poverty and social exclusion of this population are highlighted, also making recommendations on ways to improve care-leavers’ outcomes. | 1 | Study is driven by the theory | The capability approach requires an assessment of care-leavers’ lives; not merely the kind of lives they manage to lead, but also the freedom that they must choose between different styles and ways of living. This recommendation connects to the three principles within the capability approach, an evaluation of the situation of care-leavers at the levels of individual well-being, social arrangements, and policies and proposals for social change. |
| Achdut, N., Benbenishty, R., & Zeira, A. (2022). Labor force attachment among care leavers in Israel: A longitudinal cross-sequential between-groups design. *Child Abuse & Neglect, 133*, 105860. <https://doi.org/10.1016/j.chiabu.2022.105860> | Human capital | According to human capital theory (Becker; Mincer & Polachek; Putnam), individuals characterized by richer human capital resources, such as education, training, profession, job skills and work experience, are more likely to work, to obtain the more stable jobs, and to earn a higher wage. Individuals with fewer such resources are more prone to unemployment and job instability and are less likely to make a ‘living wage’. Continuous employment enhances social capital and support networks. Growing up in a youth village (vs family) is thought to possibly explain the higher employment among care-leavers than other youth. Some people with low human capital land up in stable employment, perhaps because of necessity, though low-quality work. Others with good human social capital had fragmented and slow employment. Those with low education, non-normative discharge from the military were most disconnected from employment. | 1 | Theory informed what data was collected | Interventions should promote labour market integration while still in care and afterward. Those who are vulnerable need additional support. |
| Miller, J. J., Chih, M.-Y., & Washington, E. (2016). Conceptualizing a mobile app for foster youth transitioning to adulthood: A mixed-method approach. *Journal of Technology in Human Services, 34*(2), 145-170. <https://doi.org/10.1080/15228835.2015.1108260> | Human development | Care-leavers wanted to use the App to connect with other care-leavers, which is age appropriate for 19-year-olds. Human development theories explain young people’s need to seek out meaningful relationships (Erikson; Bowlby). | 3 | Theory mentioned in passing in the discussion | The development of apps for care-leavers should build in social networking capabilities, particularly within the care-leaver group (as distinct from general social networking on Facebook, etc.). |
| Atwool, N. (2020). Transition from care: Are we continuing to set care leavers up to fail in New Zealand? *Children and Youth Services Review, 113*, 104995. <https://doi.org/10.1016/j.childyouth.2020.104995> | Identity | Notions of identity capital (Lee & Berrick), self-reliance, and agency are important for leaving care. These are explained. | 1 | This is a theoretical or conceptual paper | Young people need space and opportunity to engage in self-work, to address emotional and identity needs. |
| Colbridge, A. K., Hassett, A., & Sisley, E. (2017). “Who am I?” How female care leavers construct and make sense of their identity. *SAGE Open, 7*(1). <https://doi.org/10.1177/2158244016684913> | Identity | Identity (Erikson; Gillian) is shaped by early adverse environments and trauma, impacting on how female care-leavers make sense of themselves today and how this plays out in life. | 2 | Theory is present, but not well-developed as informing the study | Clinicians to provide a holding space for trauma in which identity can be explored. Assist caregivers to understand care-leavers’ trauma and its impact on current behaviour. |
| Munford, R., & Sanders, J. (2015). Negotiating and constructing identity: Social work with young people who experience adversity. *British Journal of Social Work, 45*(5), 1564-1580. <https://doi.org/bjsw/bcu059> | Identity | “Three key themes emerged as being significant in contributing to positive identity development: seeking safe and secure connections; finding opportunities to test out identities; and building a sense of agency” (p. 1564). Gidden’s theory of the self as identity development is used. | 1 | Study driven by theory | Social workers can be more responsive to the unique and atypical identity-building initiatives of care-leavers, e.g., understanding hidden resilience. Social workers can help by creating safe, uninterrupted, accepting spaces for care-leavers, where they can work to develop an understanding of themselves – who am I. Providing practical support. |
| Artamonova, A., Guerreiro, M. d. D., & Höjer, I. (2020). Time and context shaping the transition from out-of-home care to adulthood in Portugal. *Children and Youth Services Review, 115*, 105105. <https://doi.org/10.1016/j.childyouth.2020.105105> | Identity capital | Identity capital (Côté) helps explain why social care-leavers transition more effectively than others – the various resources they can draw around them. Agency plays a role here. Links to being resilient and to positive future orientation. Social capital, human capital and personal agency all emerged in the study as important factors in leaving care. In earlier generations, young people in care got more education than those living with families, and so care-leavers often did better than those growing up at home. | 1 | Theory informed both data collection and analysis | Educational achievement while in care is vital to post-care outcomes. Social networks are important enablers of care-leaving outcomes. |
| Rambajue, R., & O’Connor, C. (2022). Intersectional individualization: Toward a theoretical framework for youth transitioning out of the child welfare system. *Journal of Public Child Welfare, 16*(2), 156-176. <https://doi.org/10.1080/15548732.2020.1856284> | Individualization | This study combines individualisation theory (Beck) and intersectionality theory (Crenshaw) to explain poor outcomes among some care-leavers. The individualisation component of the authors’ combining of “intersectional individualization” emphasises the challenges individuals face in navigating a changing society. This can be helpfully collated with intersectionality, as the authors do in their paper. | 1 | Study is based on this theory | The authors pose a range of questions to practitioners based on the theorizing, i.e. are programmes designed to help young people transitioning out of the child welfare system too one-size-fits-all or to set against middle-class, heterosexual, white, masculine norms and expectations, and questions the rigidity of the child welfare system. |
| Bennwik, I.-H. B., Oterholm, I., & Kelly, B. (2023). ‘My disability was my own responsibility’: An institutional ethnography of the transitional experiences of disabled young people leaving care. *Children and Youth Services Review, 146*, 106813. <https://doi.org/10.1016/j.childyouth.2023.106813> | Institutional ethnography | Institutional ethnography (Lund & Nilsen) is both an explanatory theory and methodology. Regarding the former, it considers the interrelationship between individuals and organisations. It considers ruling relations, often in texts, often implicit, that determine patterns of interaction. | 1 | Study was informed and shaped by the theory | The interactions between agency and structure in institutions of aftercare for disabled youth need to be questioned. The notion of a ‘successful transition’ for a disabled youth needs to be questioned and explicated. |
| Bennwik, I.-H. B., Oterholm, I., & Kelly, B. (2023). ‘Disability is not a word we use’: Social workers’ professional judgements about support for disabled young people leaving care. *Child & Family Social Work, 28*(2), 443-453. <https://doi.org/10.1111/cfs.12975> | Institutional logics | Institutional logics (Friedland & Alford) helps unpack the interconnections between individuals, institutions and organisations, revealing organising principles, and the ways behaviour and perceptions are constrained or promoted. This helped to uncover social workers’ perceptions and constructions of disabled youth and the services they are eligible for. Three logics emerged: medical, activation and aftercare logics. | 1 | Study was informed and shaped by the theory | Multiagency work is recommended, to enable more flexible services for different client systems. |
| Oterholm, I., & Paulsen, V. (2018). Young people and social workers’ experience of differences between child welfare services and social services. *Nordic Social Work Research, 8*(sup1), 19-29. <https://doi.org/10.1080/2156857X.2018.1450283> | Institutional logics | In this article, the theory of institutional logics (Friedland & Alford) provides a way to comprehend the social workers’ considerations and the young adults’ experiences in relation to the institutional conditions they face. Based on the theory of institutional logics, the differences between the social workers’ judgment as well as the young people’s different experiences could be understood by how different institutional logics take into account different dimensions of support. These differences are important to have in mind when deciding how young people with a child welfare background should be supported. Young people often struggle in their transition to adulthood and are in need of support. | 1 | Findings linked to theory | Social services’ focus on immediate independence implies less support than other young people get and less than child welfare services tend to provide. However, if a young person needs help to get a job or would benefit from activation measures, social services can provide relevant support. The findings in this article suggest that it is important which agency young people receive support from. Social services cannot replace child welfare services when it comes to offering support for young people leaving care. |
| Disney, T., & Walker, C. (2023). Young people leaving care and institutionalised vulnerability in the Russian Federation. *Children & Youth Services Review, 155*, 107225. <https://doi.org/10.1016/j.childyouth.2023.107225> | Institutionalised vulnerability | Institutionalised vulnerability (Stryker; Stein) refers to an interaction of vulnerabilities that impact care-leavers. The focus is on the range of interacting systems: systemic, structural, and discursive. This conceptual framing led to uncovering a range of connected vulnerabilities among care-leavers in Russia. These institutionalised vulnerabilities impact transitions to education, work and safe housing. | 1 | The theoretical concept is used in the research and discussions | More attention should be given to institutionalised vulnerability as young people prepare to age out of care. Services are needed that counteract these vulnerabilities. |
| Atwool, N. (2020). Transition from care: Are we continuing to set care leavers up to fail in New Zealand? *Children and Youth Services Review, 113*, 104995. <https://doi.org/10.1016/j.childyouth.2020.104995> | Interdependence | Neuroscience is mobilised to emphasise the social scaffolding of adolescence, i.e. the need for interdependence (Avery & Freundlich). | 1 | This is a theoretical or conceptual paper | Notions of independence for young people should be universally replaced with interdependence. This notion is embedded in Māori culture. |
| Bennwik, I.-H. B., & Oterholm, I. (2021). Policy values related to support for care leavers with disabilities. *European Journal of Social Work, 24*(5), 884-895. <https://doi.org/10.1080/13691457.2020.1751589> | Interdependence | Rather than independence (or dependence) interdependence (Stiver) implies mutual connections between young people leaving care and others around them. Policy documents overemphasise independence. | 1 | Interdependence informed data collection and analysis | The notion of independence needs to be decentred, to create space for a positive view on interdependence. |
| Storø, J. (2018). To manage on one’s own after leaving care? A discussion of the concepts independence versus interdependence. *Nordic Social Work Research, 8*(sup1), 104-115. <https://doi.org/10.1080/2156857X.2018.1463282> | Interdependence | This paper endeavours to conceptualise, even theorise, the term ‘interdependence’, which is offered as an alternative to the typical focus on ‘independence’ in care-leaving discourses. | 1 | This paper is a theoretical or conceptual paper | More focus is required on interpersonal skills (such as negotiation, cooperation and communication) than on independent living skills (cooking, cleaning). Interdependence prompts practitioners to help young people identify care-leaving needs, but not to satisfy them for the young person. Interdependence does include some attention to independence and self-efficacy. |
| Bhargava, R., Chandrashekhar, R., Kansal, S., & Modi, K. (2018). Young adults transitioning from institutional care to independent living: The role of aftercare support and services. *Institutionalised Children Explorations and Beyond, 5*(2), 168-187. <https://doi.org/10.1177/2349301120180207> | Intersectionality | The intersection (Bastia) of gender (being female) and being a care-leaver could help explain the experiences of female care-leavers in India | 3 | Mentioned in passing as a possible research lens | Suggest intersectionality (specifically gender and care-leaving) be used in future research on female care-leavers. |
| McCormick, M., Anthony, J., & Townsend, E. T. (2023). Fostering safer spaces: Retaining and empowering SGM students with a lived history of foster care. *Child & Adolescent Social Work Journal, 40*(2), 255-270. <https://doi.org/10.1007/s10560-022-00889-7> | Intersectionality | This study is a narrative literature review that aims to provide context for understanding the life experiences of SGM (Sexual and gender minorities) including those who identify as lesbian, gay, bisexual, transgender, asexual, and queer, among other identities and to provide recommendations for best practices to support SGM. Intersectionality is a term coined by Crenshaw and further developed by Collins to describe the ways in which multiple aspects of identity can intersect and result in particular forms of oppression and privilege. | 1 | Theory is used as a conceptual base and to interpret findings | Following are best practices related to programs and policies, safe spaces, a welcoming cul­ture, language, identity awareness, and health supports. |
| Rambajue, R., & O’Connor, C. (2022). Intersectional individualization: Toward a theoretical framework for youth transitioning out of the child welfare system. *Journal of Public Child Welfare, 16*(2), 156-176. <https://doi.org/10.1080/15548732.2020.1856284> | Intersectionality | This study combines individualisation theory (Beck) and intersectionality theory (Crenshaw) to explain poor outcomes among some care-leavers. The intersectionality component of the authors’ combining of “intersectional individualization” creates a foundation for understanding how people possess multiple, interconnected, and indivisible social identities that shape care-leavers’ experiences and how their experiences with systems of power could present as marginalization, oppression, or privilege. | 1 | Study is based on this theory | Theory-informed practice recommendations concern questioning whether programmes assume a narrow, normative profile of care-leaver, and providing more inclusive services to diverse client groups. |
| Hlungwani, J., & van Breda, A. D. (2020). Female care leavers’ journey to young adulthood from residential care in South Africa: Gender‐specific psychosocial processes of resilience. *Child & Family Social Work, 25*(4), 915-923. <https://doi.org/10.1111/cfs.12776> | Journey towards independent living | Van Breda’s ‘Journey towards independent living’ theory is a grounded theory model that explains the psychosocial processes care-leavers engage in to achieve better-than-expected outcomes. This study confirmed the model for women and two additional female-specific processes were identified. | 1 | ‘Journey towards independent living’ theory drove the study | The care-leaving processes found should be fostered among children in care, to facilitate better-than-expected outcomes as they transition from care. |
| Van Breda, A. D. (2015). Journey towards independent living: A grounded theory investigation of leaving the care of Girls & Boys Town South Africa. *Journal of Youth Studies, 18*(3), 322-337. <https://doi.org/10.1080/13676261.2014.963534> | Journey towards independent living | The ‘Journey towards independent living’ theory (van Breda) emerged from a grounded theory study which identifies the processes that young people go through as they make the transition. Working from a resilience viewpoint, with an ecological emphasis, four central psychosocial processes arose that together explain the care-leaving experiences of the participants. | 1 | The theory is used to identify the psychosocial process of resilience. Links are made to the processes and the resilience theory. | Recommends that these processes be developed in young people while still in care, such as using a peer governance system to promote a belief in self and learning through mistakes. |
| Van Breda, A. D., & Hlungwani, J. (2019). Journey towards independent living: Resilience processes of women leaving residential care in South Africa. *Journal of Youth Studies, 22*(5), 604-622. <https://doi.org/10.1080/13676261.2018.1523541> | Journey towards independent living | This study focuses on the ‘journey towards independent living’ theory (van Breda), the resilience processes that facilitate better transitions of young women out of care and towards independent living with aim of determining to what extent the resilience process of young women are similar to those of men. This study is conducted with only female care-leavers. | 1 | The entire study is based on the use of this theory with female participants. | “The authors conclude that resilience-building programmes can be implemented to prepare both males and females for leaving care” (p. 619). Practitioners working with young people leaving care can do similar work with both boys and girls in developing their capacity to apply these social processes during their transition from care. |
| Jensen, S. G. (2021). The rebuilding of fragmented memories, broken families and rootless selves among Danish care leavers. *Journal of Family History, 46*(1), 77–91. <https://doi.org/10.1177/0363199020967582> | Kinning | A Danish study that aimed was to explore the tangible and creative practices and processes by which memories and relation created, repaired, strengthened, and are shaped through narrative and physical paraphernalia and how, care-leavers change their self-image and their sense of belonging. Howell’s concept of “kinning” was used to describe the practices by which care-leavers seek to create and maintain, as well as repair and manage, their fragile relationships with people whom they consider to be part of their family and heritage. | 2 | Theory described in opening pages and in discussion section. | No theory-informed practice recommendations provided. |
| Gomez, R. J., Ryan, T. N., Norton, C. L., Jones, C., & Galán-cisneros, P. (2015). Perceptions of learned helplessness among emerging adults aging out of foster care. *Child & Adolescent Social Work Journal, 32*(6), 507-516. <https://doi.org/10.1007/s10560-015-0389-1> | Learned helplessness | Learned helplessness (Seligman) may impair care-leavers’ outcomes. Examined role of learned helplessness in homelessness among care-leavers and non-care-leavers. Found higher degrees of learned helplessness among care-leavers, related to experiences of abuse, placement disruption, etc. | 1 | Study question is theory driven | Create opportunities for care-leavers to learn how to manage adulthood and independence, e.g. opportunities to learn to drive, maintain their own schedule, contact their biological family, etc. Also opportunity for exposure to vocational and educational opportunities. |
| Achdut, N., Benbenishty, R., & Zeira, A. (2022). Labor force attachment among care leavers in Israel: A longitudinal cross-sequential between-groups design. *Child Abuse & Neglect, 133*, 105860. <https://doi.org/10.1016/j.chiabu.2022.105860> | Life course | Life course (Brady & Gilligan) used to track labour force attachment over 21-34 years, differentiating between before coming into care, immediately after leaving care, military service, post-military employment. Some people with low family social capital land up in stable employment, perhaps because of necessity, though low-quality work. Others with good family social capital had fragmented and slow employment. Those with low education or non-normative discharge from the military were most disconnected from employment. | 1 | Theory informed what data was collected | Interventions should promote labour market integration while still in care and afterward. Those who are vulnerable need additional support. |
| Adeboye, T. K., Guerreiro, M. D., & Höjer, I. (2019). Unveiling the experiences of young people in foster care: Perspectives from Portugal and Nigeria. *International Social Work, 62*(1), 433-446. <https://doi.org/10.1177/0020872817731147> | Life course | Developed a life course (Elder) informed model of care-leaving, addressing past, present and future, which guided the collection of data according to these phases. Past experiences impact on present and anticipated future experiences. | 1 | Theory informed data collection tool and an a priori model that was used for data analysis | Not clear how Life Course approach influences practice, other than the recognition that the past influences the present and expectations about the future. “There is need for care organisations to focus on the strength of youth during each phase through vocational and entrepreneurial skills aside from educational qualification.” |
| Artamonova, A., Guerreiro, M. d. D., & Höjer, I. (2020). Time and context shaping the transition from out-of-home care to adulthood in Portugal. *Children and Youth Services Review, 115*, 105105. <https://doi.org/10.1016/j.childyouth.2020.105105> | Life course | Life course (Elder) helps to explain the intergenerational differences in life passages – progress through life is embedded in proximal and distal situations in one’s environment. | 1 | Theory informed both data collection and analysis | It is important to consider the macro contexts into which young people transition, as these contexts shape opportunities and obstacles they experience. Men and women have different life courses, because of the gendered structuring of society. |
| Bengtsson, M., Sjöblom, Y., & Öberg, P. (2018b). Young care leavers’ expectations of their future: A question of time horizon. *Child & Family Social Work, 23*(2), 188-195. <https://doi.org/10.1111/cfs.12399> | Life course | The care-leaving journey should be seen as evolving over time, over the life course (Gield & Elder) and within and in interaction with the care-leavers’ social context. The context is the care setting and the young person’s dependence, which they are expected to relinquish. | 1 | Life course is introduced as the theoretical perspective of the study, linked to the study context, and appears in the discussion. | No theory-informed practice recommendations provided. |
| Boman, M. (2022). Relationships with family members and transition from out-of-home care: Unfinished business. *Children and Youth Services Review, 143*, 106662. <https://doi.org/10.1016/j.childyouth.2022.106662> | Life course | The transition from care is framed within life course theory (Elder): various social and historical contexts, as well as personal agency, transitions and turning points. | 2 | Theory introduced up front, but not pulled through noticeably in analysis and discussion | Conversations about family relationships is crucial, including the limits of these relationships. |
| Brady, E., & Gilligan, R. (2018). The life course perspective: An integrative research paradigm for examining the educational experiences of adult care leavers? *Children and Youth Services Review, 87*, 69-77. <https://doi.org/10.1016/j.childyouth.2018.02.019> | Life course | Life course (Elder) (which includes concepts of turning points and transitions) is useful in focusing on long-term sequences of life experience that shape current relationships and functioning. Recognises the cumulative effect of life experiences over years – from family, into care (often multiple care contexts) and out of care (often into multiple post-care contexts). Seen as compatible with other theories, such as social ecology, resilience and actor network theories. | 1 | Study is grounded in the life course approach and its theoretical constructs are used to understand care-leaving | Recommend greater use of the life course approach to inform research methods on care-leavers. |
| Brady, E., & Gilligan, R. (2020a). The role of agency in shaping the educational journeys of care-experienced adults: Insights from a life course study of education and care. *Children & Society, 34*(2), 121-135. <https://doi.org/10.1111/chso.12361> | Life course | The study draws on life course theory (Elder). Life course manifests in their authors’ interest in the long narrative of leaving care. | 1 | Study informed by theoretical questions regarding life course | Recommendations implicitly focus on the long journey from care to adulthood, rather than overly focusing on single incidents. |
| Dumollard, M., Gauthier-Davies, C., Goyette, M., & Blanchet, A. (2023). The transition to adulthood of dual-system youth in Quebec: A focus on high school graduation and housing situation. *Child and Adolescent Social Work Journal*, Advance online publication. <https://doi.org/10.1007/s10560-023-00944-x> | Life course | Life course theory (Elder) is used to explain care-leavers who have a dual history of protective care and juvenile justice care – they have poorer outcomes than care-leavers with just a child protection care history. | 3 | Theory is briefly mentioned before the methodology and not again | No theory-informed practice recommendations provided. |
| Gabriel, T., Keller, S., & Bombach, C. (2021). Vulnerability and well-being decades after leaving care. *Frontiers in Psychology, 12*. <https://doi.org/10.3389/fpsyg.2021.577450> | Life course | Study has a long-term focus on leaving care, hence awareness of different stages of the life course (Gilligan). | 2 | Theory is present but not influential | Care-leaving pathways are not determined, but are shaped by social networks, being recognised by others, and a degree to freedom inside and outside the children’s home influence life course. |
| Höjer, I., & Oterholm, I. (2023). Foster carers’ views of the transition from foster care to adulthood for young people with mental health problems from a life-course perspective. *Nordic Social Work Research*, Advance online publication. <https://doi.org/10.1080/2156857X.2023.2298680> | Life course | Life course theory (Elder) posits that what happens in one life period is connected to other periods. This paper focuses particularly on linked lives and timing of lives to understand how “foster carers’ relations with and follow-up of the cared-for young people are influenced by the young people’s mental health problems”. | 1 | Life course theory shapes the study from concept to analysis | The paper notes only the importance of recognising the impact of mental health on care-leaving. |
| Keller, S., Gabriel, T., & Bombach, C. (2021). Narratives on leaving care in Switzerland: Biographies and discourses in the 20th century. *Child & Family Social Work, 26*(2), 248-257. <https://doi.org/10.1111/cfs.12813> | Life course | The aim of the study was to explore the narrative methodologies and to ground a theory on biographies after care. The study analyses those pathways and their junctions in detail; however, it was not limited to individual life courses. Rather focused on experiences in care transitions and after-care lives which increased the understanding of the qualities of the experiences beyond the individual. Hence a grounded approach. The analysis was based on a life course perspective (Sackmann & Wingens). | 1 | The entire study draws on life course perspective | The authors based on the life course theory, suggest adjusting care-leaver support to individual needs, creating enabling and empowering logic in decision-making, and prioritizing individual opportunities over formal goals and measurable competences. Authors recommend 1. Support for care-leavers should be adapted to their individual needs and should avoid addressing the child as an ‘object of care’ by forgetting or stigmatizing them, 2. A logic of enabling and empowering should be created in decision making, relationships and structures, 3. There should be a focus on individual opportunities in and after care, instead of (only) on formal goals and measurable competences. |
| Mendes, P. (2018). Towards the social inclusion of young people transitioning from out-of-home care: An examination of the Home Stretch campaign. *Social Alternatives, 37*(1), 59-62. <https://openjournals.library.sydney.edu.au/SWPS/article/view/12920/12020> | Life course | Life course theory emphasises that the CL trajectory is not stable or consistent, but varies between individuals. Theory not cited. | 3 | Mentioned in passing | Aftercare can facilitate turning points for care-leavers. |
| Mølholt, A. K. (2021). ‘Sometimes I feel 60, sometimes I feel 13’: Perceptions of age among vulnerable young people in Denmark. *Journal of Youth Studies, 24*(1), 62-76. <https://doi.org/10.1080/13676261.2019.1687861> | Life course | Care-leavers do not follow a normal biographical life course, with the result that their perception of their age may also not be normative. Findings show that both the life course (Kupferberg) and the perception of their age vary considerably from a normative linear progression. Not fitting into the normative life course can be uncomfortable and disorienting. | 1 | Theory driven study | Need flexible, person-centred constructions of age, rather than normative ages (e.g., 18 or 21). Include children in age-normative activities so that they bridge the disjuncture between their perceived age/life course and their actual age/life course. |
| Power, L., & Raphael, D. (2018). Care leavers: A British affair. *Child & Family Social Work, 23*(3), 346-353. <https://doi.org/10.1111/cfs.12421> | Life course | Life Course model (Hertzman; Due et al.) looks at the difference stages of life and the study uses the model that states the longer care-leavers experience disadvantage, the greater the adverse health effects. It incorporates a life course/pathway model to show how stages in the life course (prior to care, during care, and after care) interact with these factors to shape care-leavers’ health outcomes. | 1 | Study is based on the life course model | Authors suggested that public policies should include direct investment in social care and a broader focus on employment standards, housing development, and other social determinants of health of care-leavers due to theory pointing out the plight of care-leavers at different stages of their life. Therefore, health of care-leavers can be improved if population specific strategies and wider welfare programmes are developed and implemented. |
| Pryce, J., Napolitano, L., & Samuels, G. M. (2017). Transition to adulthood of former foster youth: Multilevel challenges to the help-seeking pProcess. *Emerging Adulthood, 5*(5), 311-321. <https://doi.org/10.1177/2167696816685231> | Life course | For youth aging out of the foster care system, these developmental tasks outlined are further complicated by their simultaneous exit from the system. While many young adults from more advantaged backgrounds navigate this transition with extensive help from family, care-leavers do not have such resources available. Life-course theory (Elder) stresses the importance of considering young people’s transition to adulthood within the larger personal, intrapersonal and systemic levels. | 3 | Mentioned twice in literature review only | Authors recommend support that directly, builds on relational network to be considered for practice and the interpersonal relationship skills trainings, in preparing young people to age out. Peer- and group-based interventions, including group mentoring and supportive networks with other young people aging out. |
| Refaeli, T., Mangold, K., Zeira, A., & Kongeter, S. (2017). Continuity and discontinuity in the transition from care to adulthood. *British Journal of Social Work, 47*(2), 325-342. <https://doi.org/10.1093/bjsw/bcw016> | Life course | The study uses a life course perspective (Grunwald & Thiersch). The study examines patterns of continuity and discontinuity in care-leavers’ transition to independent living. The study finds that while care-leavers value continuity, they also value and create discontinuity. | 1 | Life course theory is used in the literature section and analysis. | Social workers should recognize that continuity and achievement of the young people; Social workers need to get a better understanding of the dynamics and interactions between different paths that care-leavers face; Social work practice needs to recognize that stability in life courses is not only an outcome of continuous relationships, but also of places and activities. |
| Reimer, D., & Schäfer, D. (2015). The use of biographical narratives to explain favourable and unfavourable outcomes for children in foster care. *Adoption & Fostering, 39*(1), 5-20. <https://doi.org/10.1177/0308575914565084> | Life course | The study used the life course theory (Elder) to differentiate how favourable life courses differ from unfavourable ones, with the assumption that all of the persons involved started life under extremely difficult circumstances. | 1 | Life course is used in literature and analysis section | No theory-informed practice recommendations provided. |
| Spallek, M., & Haynes, M. (2021). Post-school transitions for children in out-of-home care: Evidence from the Australian Census Longitudinal Dataset. *Journal of Youth Studies, 24*(7), 901-924. <https://doi.org/10.1080/13676261.2020.1781803> | Life course | Life course theory (Elder) is the overarching theoretical framework in which past experiences, decisions and relationships are considered to influence transitions later in life. Applying the life course approach to educational achievement of children implied that the pursuit of further education following secondary school is a culmination of past experiences up to the point of transition. This aspect was used in the study. | 1 | Study was based on this framework | No theory-informed practice recommendations provided. |
| Bengtsson, M., Sjöblom, Y., & Öberg, P. (2020). Transitional patterns when leaving care: Care leavers’ agency in a longitudinal perspective. *Children and Youth Services Review, 118*, 105486. <https://doi.org/10.1016/j.childyouth.2020.105486> | Life course – agency | Agency (Hitlin & Elder), which is located within the broader life course theory, helps to inform how individuals navigate social systems and structures. Various categories of agency are identified. | 1 | The study was strongly directed by the theory | Agency should be fostered in disengagement planning, as this appears to shape aftercare outcomes. Services should be bespoke for individual young people. A gradual and individualised transition is important. |
| Solem, M.-B., Helgeland, I. M., Brannen, J., & Phoenix, A. (2020). Transitions to adulthood of ‘at risk’ young men: New analysis from two Norwegian qualitative longitudinal studies. *Children & Society, 34*(3), 220-233. <https://doi.org/10.1111/chso.12369> | Life course – agency | The study considers how male care-leavers, who had evidenced serious behavioural risks, exercised agency, showing how “agency is socially and structural located in intergenerational and other social relations”. Agency (Elder) is a component of the life course perspective. Participants were able to exercise agency to facilitate turning points, despite the challenges of their childhoods. | 1 | Agency directs the study from conceptualisation to findings | No theory-informed practice recommendations provided. |
| Zeira, A., Refaeli, T., Achdut, N., & Benbenishty, R. (2023). Economic self-sufficiency and the employment outcomes of care leavers: A 10-year follow up. *Child & Family Social Work, 28*(3), 635-645. <https://doi.org/10.1111/cfs.12990> | Life course – human capital | The longitudinal study examines the employment and economic self-sufficiency outcomes of care-leavers in Israel over a 10-year period, examining changes over time. Study mentions the life course perspective and its component human capital (Hutchison). In line with the life course perspective, the findings of the study highlight the inherent dependency between experiences in different life stages. | 2 | Theory provided early on, but does not appear in findings, and only once in discussion | Promote employment for care-leavers, to build skills, integrate into job market and secure better salaries. State should continue to support young people into young adulthood. |
| Achdut, N., Benbenishty, R., & Zeira, A. (2023). Labor market position among care leavers and their matched peers: A longitudinal comparative study. *Child Abuse & Neglect, 145*, 106406. <https://doi.org/10.1016/j.chiabu.2023.106406> | Life course – human capital, social capital | Human capital (Becker; Mincer & Polachek) and social capital (Putnam) factors, which are components of life course theory (Brady & Gilligan), are used to predict labour market participation at different points in the life course. Human capital was measured by educational attainment, psychotechnical score and post-care enrolment in higher education. Social capital was measured by pre-care family context, post-care family-related difficulties, completion of military service, and socioeconomic circumstances, among others. Three groups (clusters) of care-leavers were identified through the study. | 1 | Study was guided by explicit theoretical constructs and model | Theory-informed practice recommendations included promoting labour market participation by training in fields where skills are needed (e.g., information technology). Care staff and Israeli Defence Forces can cooperate to facilitate the transition from care to military service. Post-care policies are required for those who drop out of employment soon after leaving care. |
| Brady, E., & Gilligan, R. (2020b). Supporting care-experienced adults’ educational journeys: “Linked lives” over the life course. *Child & Family Social Work, 25*(2), 221-229. <https://doi.org/10.1111/cfs.12677> | Life course – linked lives | Life course theory (Elder; Hutchison) informs this study. It is argued that the educational journey of care-leavers over time is embedded within and shaped by networks of relationships – linked lives. Family is strongly foregrounded in linked lives, thus the role of family in shaping educational pathways of care-leavers. | 1 | Study set out to examine the utility of the theory for care-leaving | Recommend mobilising a wide range of family-like relationships, which can continue over time. |
| Gilligan, R., & Brady, E. (2023). What helps adult care leavers return to education? Exploring the relevance of learner identity and the life course perspective. *Journal of Youth Studies, 26*(10), 1373-1386. <https://doi.org/10.1080/13676261.2022.2098706> | Life course – linked lives | Linked lives (Hutchison) emphasises interdependence between people over the life course (Mayer). Support, learner identity, and educational memories emerge as factors that facilitate care-leavers returning to education | 1 | Study set out to explore how linked lives influence educational re-engagement | Networks of relationships are crucial in facilitating educational engagement after dropping out of education. The motivation of others may be greater than the motivation of the care-leaver. |
| Lemus, D., Farruggia, S. P., Germo, G., & Chang, E. S. (2017). The plans, goals, and concerns of pre-emancipated youth in foster care. *Children and Youth Services Review, 78*, 48-55. <https://doi.org/10.1016/j.childyouth.2017.05.003> | Lifespan theory of control | Lifespan theory of control (Schulz et al.) informed a study on care-leavers’ plans, goals and concerns for the immediate (1 year) and longer term (10 year) futures. Many participants felt they had control (agency) over educational and occupational plans and goals. Interestingly, some participants had no plan and no worries or concerns about their future. | 1 | Study driven by theory | Recommend that “youth in care would benefit from services focused on both immediate planning and goal setting prior to emancipation” (p. 54). |
| Bengtsson, M., Sjöblom, Y., & Öberg, P. (2018b). Young care leavers’ expectations of their future: A question of time horizon. *Child & Family Social Work, 23*(2), 188-195. <https://doi.org/10.1111/cfs.12399> | Liminality | There is a period between leaving the care setting (exit) and becoming independent (entry) in which the care-leaver is floating (liminal), characterised by uncertainty and not belonging. Short-term expectations are anxious while long-term expectations are positive, at both times of data collection (exit and liminal). A theory of liminality is not provided and no citation to the theory. | 3 | Liminality is used as a phase of leaving care, not as a theory. | No theory-informed practice recommendations provided. |
| Glynn, N. (2021). Understanding care leavers as youth in society: A theoretical framework for studying the transition out of care. *Children and Youth Services Review, 121*. <https://doi.org/10.1016/j.childyouth.2020.105829> | Liminality | Liminality (Turner) describes the journey from adolescence to young adulthood – an in-between space involving separation, margin/transition and reintegration. It can result in marginalisation. Liminality is located in social relationships. | 1 | Data collection was structured around the theoretical concepts | Care-leavers need a liminal space in which to explore, make mistakes, and value their lives. |
| Glynn, N., & Mayock, P. (2023). Housing after care: understanding security and stability in the transition out of care through the lenses of liminality, recognition and precarity. *Journal of Youth Studies, 26*(1), 80-97. <https://doi.org/10.1080/13676261.2021.1981838> | Liminality | Liminality (Furlong) describes the journey from adolescence to young adulthood – an in-between space involving separation, margin/transition and reintegration. It can result in marginalisation. Liminality is located in social relationships. Housing helps to bridge the gaps between life spaces. Secure housing creates a safe liminal space in which young people can navigate the larger life transitions. | 1 | Data collection was structured around the theoretical concepts | Policies are needed that permit care-leavers to remain in their placement or to live in time-secure (stable) housing. |
| Anghel, R. (2021). From ‘the New Man’ to care-leaver activists: Communist and contemporary discourses shaping 50 years of leaving care in Romania. *Child & Family Social Work, 26*(2), 258-269. <https://doi.org/10.1111/cfs.12819> | Marxism | In Romania, Marxist-Leninist ideas that the state could replace parents shaped out-of-home care for children (Dragoi). Bowlby’s theory of attachment was rejected. | 2 | Theory used to narrate the history of care-leaving | Further work is required to develop a rights-based construction of out-of-home care and care-leaving. |
| Sulimani-Aidan, Y., Melkman, E., & Greeson, J. K. P. (2020). The contribution of mentoring to the life skills of youth leaving care in Israel. *Child & Family Social Work, 25*(2), 345-354. <https://doi.org/10.1111/cfs.12690> | Mentoring | The mentoring theory (Greeson), states that the “process of creating positive change in the youths’ lives involves mentors serving different functions in the relationship and acting as parental figures, role models, and life coaches” (p. 347). This study aimed to examine the relationship between mentoring characteristics or functions and outcomes. | 1 | The study uses aspects of the mentoring theory in its literature and analysis. | The authors recommends that preparation programs be expanded and that care centers should integrate natural mentors as part as their intervention plan and future studies can be looked that include the perspectives of both mentors and youth. |
| Paul, J. C. (2020). Exploring support for LGBTQ youth transitioning from foster care to emerging adulthood. *Children and Youth Services Review, 119*, 105481. <https://doi.org/10.1016/j.childyouth.2020.105481> | Minority stress | This study aimed to generate knowledge about how supportive resources may be cultivated for LGBTQ transitioning youth in order to facilitate more positive outcomes for this population. Minority stress theory (Henderson & Testa) highlights the impact of minority stress on LGBTQ foster youth, highlighting the connection between anti-LGBTQ experiences and psychological distress, thereby guiding individuals towards promoting supportive environments for their well-being. | 2 | Study states that it is based on this framework, but mentioned only once in findings and discussion | Facilitate trainings for foster parents, caregivers, and others (i.e. child welfare agency employees, service providers) to better support LGBTQ transitioning foster youth. Training should particularly address handling stigma and discrimination. Services should be more tuned into the vulnerability and needs of LGBTQ youth. |
| Mendis, K., Gardner, F., & Lehmann, J. (2015). The education of children in out-of-home care. *Australian Social Work, 68*(4), 483-496. <https://doi.org/10.1080/0312407X.2014.963134> | Motivation | Weiner’s motivation theory is used. The study found that participants who returned to education later in life after realising its important (delayed group) “believed that their lack of confidence in their ability at school resulted from adults’ comments which diminished their already low self-esteem” (p. 491). They gave up on learning because they perceived themselves to lack the ability to learn. | 3 | Theory briefly mentioned in discussion of one finding | No theory-informed practice recommendations provided. |
| Mendis, K., Lehmann, J., & Gardner, F. (2018). Promoting academic success of children in care. *British Journal of Social Work, 48*(1), 106-123. <https://doi.org/10.1093/bjsw/bcx029> | Motivation | Weiner’s motivation theory is used. Study found that participants who returned to education later in life after realising its important (delayed group) perceived themselves to lack the ability to learn, leading to learned helplessness and abandoning their education. | 3 | Theory briefly mentioned in discussion of one finding | Build opportunities to develop children’s motivation to study |
| Gross, J. P., Geiger, J. M., Uhls, E., & King, G. (2023). The relationship between financial aid and postsecondary completion among youth formerly in foster care. *Child and Adolescent Social Work Journal, 40*(2), 207-219. <https://doi.org/10.1007/s10560-022-00884-y> | Multilevel model of college access | The multilevel model of college access (not cited) incorporates constructs from economic and sociological theories of college choice and access. The model nests human capital theory within four layers that recognize the social and cultural influences on whether and where people enrol in college. These layers are (1) the individual’s habitus, (2) the school and community context, (3) the higher education context, and (4) the broader social, economic and policy context. Financial aid predicted postsecondary completion, but other sources of support did not. | 1 | Theoretical model informed the selection of constructs to measure and model | A range of supports for care-leavers in postsecondary education is needed to get them through their education, including social support, postsecondary education support services, academic support services, and employment. |
| Nsonwu, M. B., Dennison, S., & Long, J. (2015). Foster care chronicles: Use of the arts for teens aging out of the foster care system. *Journal of Creativity in Mental Health, 10*(1), 18-33. <https://doi.org/10.1080/15401383.2014.935546> | Narrative therapy | The authors construct narrative therapy (an intervention) as a theory, based on narrative theories (White & Epston) that explain how narratives and their reconstruction change perceptions and experiences of the world, particularly in relation to identity. Participants reported improved self-image, self-healing and self-efficacy. | 1 | Was a practice model. | SW professionals need to develop the ability to engage with their clients by utilizing innovative and nontraditional forms of expression via the creative arts. |
| Stead, H. (2021). Rite of passage for care leavers. *Journal of Family Therapy, 43*(3), 445-457. <https://doi.org/10.1111/1467-6427.12350> | Narrative therapy | This study draws on ideas from narrative therapy (White), notably rites of passage, outsider witnessing, “co-research” and general principles of collaborative therapy. The study suggests that these approaches help to establish a secure base for care-leavers. | 1 | The theoretical base of the study, pulling through to practice | Strengthening relationships is an important factor in the therapeutic alliance. Attention should be given to the use of a reflecting team, high levels of transparency and acceptance, and consistent support by an accompanying adult. |
| Wilson, J. Z., & Golding, F. (2016). Latent scrutiny: personal archives as perpetual mementos of the official gaze. *Archival Science, 16*(1), 93-109. <https://doi.org/10.1007/s10502-015-9255-3> | Narrative-based model of identity | The study presents the experience of care-leavers as children, their own experiences of accessing the records and a ‘narrative’ model of the self (not cited) is utilised to discuss this aspect with a basic principle of the narrative-based model of identity is that the individual must have faith in their narrative(s). This theory is not explained in the article. | 1 | Analysis of the study is linked to the narrative-based model of identity. | The authors, who were past care-leavers, request for a re-evaluation of written personal records by practitioners in the field, in which the human rights of care-leavers have been impacted upon. |
| Cleaver, K. (2016). Acknowledging the struggle: Policy changes for state care leaving provisions. *Aotearoa New Zealand Social Work Review, 28*(2), 22-31. <https://doi.org/10.11157/anzswj-vol28iss2id221> | Neoliberalism | Neoliberalism (Garrett) negatively impacts the extent and depth and right to care among looked after children. It overlooks the inequalities between young people in the care system and young people in their family of origin. | 2 | Study is informed by theory, but not testing the theory | Need for research to map out the care-leaver population and its needs, and the quality and adequacy of service provision. Particular attention is need for Maori people. Post-care support is required |
| Barboza, G. E., & Valentine, R. (2022). A network analysis of post-traumatic stress among youth aging out of the foster care system. *Children and Youth Services Review, 140*, 106589. <https://doi.org/10.1016/j.childyouth.2022.106589> | Network | Network theory (Spiller) is used to explain the interactions between trauma-related symptoms and how these contribute PTSD and impairment. The study is not primary about leaving care – the population being studied are young people aging out of foster care. The network theory-informed study was conducted using network analysis. | 1 | The study is driven by a network theory-informed understanding of PTSD | Social support is needed to help care-leavers overcome trauma. Results suggest “support targeting a sense of foreshortened future, restricted affect and feelings of detachment as modifiable risk factors that may help promote successful transitions and positive long-term outcomes into adulthood.” |
| Storø, J. (2021). Leaving care in Norway in a historic and current perspective: As a function of the Nordic welfare model. *Child & Family Social Work, 26*(2), 240-247. <https://doi.org/10.1111/cfs.12805> | Nordic welfare model | The article discusses how the Nordic welfare model, which is sometimes held up as an ideal welfare model, may represent a problematic frame for leaving care support. The ‘parental functions’ of child welfare and social services are discussed and so is the connection between the welfare model’s ability to cover up for the needs of the many versus the few and vulnerable. | 1 | This is a conceptual or theoretical paper | No practice recommendations are made, beyond raising concerns about the Nordic welfare models inadequacies in caring for young people with little family support. |
| Gabriel, T. (2023). The child: Object or subject of child care? *Children and Youth Services Review, 150*, 107022. <https://doi.org/10.1016/j.childyouth.2023.107022> | Objectification | This paper explores the subjectification (Winkler) and objectification (Nussbaum) of young people in and leaving care. The terms subject and objective have implications for children’s participation and rights. | 1 | The whole paper – a theoretical piece – centres on the theory | “Subject orientation is the necessary precondition for any child care intervention that works.” |
| Paul-Ward, A., & Lambdin-Pattavina, C. A. (2016). New roles for occupational therapy to promote independence among youth aging out of foster care. *The American Journal of Occupational Therapy, 70*(3), 1-5. <https://doi.org/10.5014/ajot.2016.017426> | Occupational justice | Occupational injustice (Townsend & Wilcock) “occurs when a person’s participation in meaningful occupation is barred, limited, undeveloped, disrupted, or marginalized. The notion is relevant to co foster care because by design, the foster care system tends to marginalize its ‘members’ by institutionalizing them, nor necessarily within physical structures bur through systemic barriers (Paul-Ward, 2009). For example, the system disrupts occupations because children are often moved from one foster care home to another.” | 2 | Theory appears only in the section corresponding roughly to implications for professional practice. | Study calls for the profession to assist young people transitioning out of care. Not much else is mentioned. |
| Kearney, K. S., Naifeh, Z., Hammer, T., & Cain, A. (2019). “Family” ties for foster alumni in college: An open systems consideration. *Review of Higher Education, 42*(2), 793-824. <https://doi.org/10.1353/rhe.2019.0015> | Open systems | The study question was informed by retention and persistence theories (in education), but open systems theory (Morgan) was used for analysis, including concepts of individuality within the system, boundaries, strange attractors. Family was present though defined flexibly; boundary management with families was important; and relationships with family members were renegotiated. | 2 | Study informed by theory at the analysis phase | College definitions of ‘family’ need to be flexible and student defined. Support services for care-leavers need to be differentiated from general services. Educate care-leavers about boundary management. |
| Stevenson, B. J. (2017). Developing a career counseling intervention program for foster youth. *Journal of Employment Counseling, 54*(2), 75-86. <https://doi.org/10.1002/joec.12055> | Person in Environment (PIE) | Poor education outcomes in always an issue with young people leaving care. Theoretically sound models for providing career-related services to foster youth are absent. This article begins the process of integrating theory and research into a comprehensive framework for providing career counselling services to foster youth. | 3 | One mention of person-environment theory is made (without citation), in reference to fitting a person to the correct career. | No theory-informed practice recommendations provided. |
| Rice, J., Mullineux, J., & Killick, C. (2022). Female care leavers experience of the staff-child relationship while living in an intensive support children’s home in Northern Ireland. *Child Care in Practice, 28*(1), 4-19. <https://doi.org/10.1080/13575279.2019.1693979> | Personal construct | Personal construct theory (Kelly) allows the researcher and the participant to analyse the meaning of the core constructs during the conversation. It explains how people make sense of themselves and their environment, through dialogue between researcher and participant. | 2 | Used in the analysis of the study | No theory-informed practice recommendations provided. |
| Nurcombe-Thorne, A., Nadesan, V. S., & Van Breda, A. D. (2018). Experiences of ‘I’ and ‘we’ among former looked-after children in South Africa. *Child and Family Social Work, 23*(4), 640-648. <https://doi.org/10.1111/cfs.12456> | Person-centered | The purpose of this study was to explore participants’ perceptions of their experience of residential care, and guided by the principles of the person-centred approach, to holistically understand their experiences. The themes were all linked to the person-centred approach theory (Rogers). For example, participants in this study experienced a lack of active, if any, involvement in decisions regarding their care, and they found that the highly structured care environment did not adequately prepare them for life after care. | 1 | Linked theory to study findings. | Authors recommend that child and youth care facilities are to provide a therapeutic milieu for all children and young people in care. Therefore, promoting both the “I” and “we” in care is a management concern, not only a practice process (p. 8). Facilities should limit the worker‐to‐child ratio to allow for more opportunities for meaningful and individualized interaction. Child and youth care workers should be passionate about children, and given specialized training to provide a genuine and caring environment for children in care. Measures to reduce staff turnover are important to secure stable placements and continuity of care (p. 8). |
| Appleton, P. (2020). Anchors for deliberation and shared deliberation: Understanding planning in young adults transitioning from out-of-home care. *Qualitative Social Work, 19*(5-6), 1130-1146. <https://doi.org/10.1177/1473325019869810> | Planning norms | “It may also be of particular importance to understand young people’s positions on planning norms, in both individual and social-cooperative contexts, given some young people’s subjection to repeated violation of norms” (Morton). Understanding a young person’s planning norms can improve collaborative planning relationship. | 1 | This is a theoretical paper | This can help engage young people who have a norm of not planning. |
| Sims-Schouten, W., & Hayden, C. (2017). Mental health and wellbeing of care leavers: Making sense of their perspectives. *Child & Family Social Work, 22*(4), 1480-1487. <https://doi.org/10.1111/cfs.12370> | Positioning | This article is underpinned by positioning theory (Harre), which is about how people communicate to locate themselves and others, including rights and duties. This theory illuminated that the typical focus on the practicalities of leaving care need to be supplemented with clear dialogue on mental health and well-being, and the role of life-skills, as these constructs are often misunderstood. | 1 | Study was shaped by positioning, from conceptualisation to empirical. | A need to tackle stigma and understanding about mental health in leaving care projects. Practitioners need to make better use of existing assessments in this respect to inform more focussed provision. The research highlights the need for programmes that address the broader transitional care needs of young care-leavers in relation to mental health. Robust transitional and evidence‐based mental healthcare and stronger collaborations between the different professionals and agencies. |
| Bond, S., & van Breda, A. D. (2018). Interaction between possible selves and the resilience of care-leavers in South Africa. *Children and Youth Services Review, 94*, 88-95. <https://doi.org/10.1016/j.childyouth.2018.09.014> | Possible selves | Studies interested in future focus often focus on a general feeling of optimism or hope about the future. Possible selves (Markus & Nurius) is interested in the content of this focus and how it motivates current behaviour. This is constructed as an enabler of resilient outcomes. In addition, developing possible selves can lead to the identification and mobilisation of other resilience resources. | 1 | Study sets out of examine this theory’s contribution to preparation for care-leaving | Recommend that possible selves be actively developed in care-leavers, which will stimulate other resilience enablers and lead to better care-leavers outcomes. |
| Dumont, A., Lanctôt, N., & Paquette, G. (2022). “I had a shitty past; I want a great future”: Hopes and fears of vulnerable adolescent girls aging out of care. *Children & Youth Services Review, 134*, 106374. <https://doi.org/10.1016/j.childyouth.2022.106374> | Possible selves | Possible selves theory (Markus & Nurius) shaped what data were collected, viz. care-leavers hopes and expectations for the future. In addition to future hopes, participants’ future selves were also motivated by past fears. | 1 | Study shaped by the theory from conception | Facilitating young people to think about and develop possible selves, and to explore the pathways to achieving those selves, will be helpful for care-leavers. Positive role models aligned with their possible selves will be helpful |
| Lanctôt, N., & Turcotte, M. (2018). The ‘good mother’ struggles: Obstacles to the attainment of motherhood ideals among adult women formerly placed in residential care. *Child & Family Social Work, 23*(1), 80-87. <https://doi.org/10.1111/cfs.12386> | Possible selves | Authors recommend the use of possible selves theory (Markus & Nurius) “that focus on positive identity as a key aspect of resilience, in order to help mothers set realistic goals about who they want to become and define means to attain their objectives. Such a theory aims to empower and give agency to individuals” (p. 85). | 2 | Theory emerges only in the implications for practice, not the study | Recommend the use of possible selves with care-leaver mothers to reflect on their own identity, including the use of the clinical, “Possible Selves Mapping Interview”. |
| Glynn, N. (2021). Understanding care leavers as youth in society: A theoretical framework for studying the transition out of care. *Children and Youth Services Review, 121*. <https://doi.org/10.1016/j.childyouth.2020.105829> | Precarity | Precarity (Berlant) refers to relationships or situations of dependence, rather than independence. Structural factors constrain the actualisation of individuals. Precarity is located in social relationships. | 1 | Data collection was structured around the theoretical concepts | Precarity conveys a message of rejection by society of themselves and of their status as a young adult. |
| Glynn, N., & Mayock, P. (2023). Housing after care: understanding security and stability in the transition out of care through the lenses of liminality, recognition and precarity. *Journal of Youth Studies, 26*(1), 80-97. <https://doi.org/10.1080/13676261.2021.1981838> | Precarity | Precarity (Berlant) refers to relationships or situations of dependence, rather than independence. Structural factors constrain the actualisation of individuals. Precarity is located in social relationships. Precarity is to explain care-leavers’ experience of aftercare policies and unaffordable housing. The lack of affordable housing increases dependence and the lack of stability, and often leads to homelessness. | 1 | Data collection was structured around the theoretical concepts | Policies are needed that permit care-leavers to remain in their placement or to live in time-secure (stable) housing. |
| Huegler, N., & Ruch, G. (2022). Risk, vulnerability and complexity: Transitional safeguarding as a reframing of binary perspectives. *Practice, 34*(1), 25-39. <https://doi.org/10.1080/09503153.2021.1932787> | Psychosocial | The authors link psychosocial perspectives to psychodynamic theories, particularly Kleinian object relations and Bion’s theories. They use these theories (particularly splitting) to highlight the false separation of safeguarding for children vs safeguarding for adults. They propose a concept of transitional safeguarding to mediate the liminal space between these two. | 1 | Concept of transitional safeguarding is theory driven | A more nuanced approach to safeguarding is required during the transition period of leaving care. |
| Natalier, K., & Johnson, G. (2015). No home away from home: A qualitative study of care leavers’ perceptions and experiences of ‘home’. *Housing Studies, 30*(1), 123-138. <https://doi.org/10.1080/02673037.2014.943698> | Psychosocial | The study provides insight into the psychosocial concept of “home” (Mallett; Saunders & Williams), as experienced by the accounts that is provided by care-leavers. An “understanding what home means and if and how it is achieved provides insight into young people’s ‘social relations, their psychology, their emotions and their “lived experience” (p. 124). | 1 | Theory is used to base the study and results are analyzed using the theory as a basis. | The author recommends that simply providing housing, which is important, but is not enough. The housing provided needs to meet care-leavers psychosocial needs and be a stable shelter so that it becomes a home, thus providing ontological security and a sense of self. |
| Törrönen, M., Munn-Giddings, C., Gavriel, C., & Morris, D. (2018). Emotional participation of young adults starting their independent living. *Nordic Social Work Research, 8*(sup1), 65-78. <https://doi.org/10.1080/2156857X.2018.1489883> | Reciprocity | Findings of the study suggest that what was vital for young young adults is the meaning and continuity of social connections which support what was termed as their “emotional participation”. The study explores emotional participation in relation to the theory of reciprocity (Morrow). | 1 | Used in the introduction and analysis of the data. Study draws on the theory of reciprocity. | Authors recommend moving from individualistic practice onto community practice which takes into consideration biological parents, extended family members and/or other collectives in this way they could have relationships with at least some of the same people they have known during care, it would make the start of independent living easier. |
| Glynn, N. (2021). Understanding care leavers as youth in society: A theoretical framework for studying the transition out of care. *Children and Youth Services Review, 121*. <https://doi.org/10.1016/j.childyouth.2020.105829> | Recognition | Recognition theory (Honneth) indicates that people desire to be recognised as distinct and valued members of society, differentiated from others and groups, leading to self-recognition. Experiences of love and caring are key. Recognition is located in social relationships. | 1 | Data collection was structured around the theoretical concepts | Relationships are central to recognition, to feel loved and respected, thus relationship-based social work practice is important. |
| Glynn, N., & Mayock, P. (2023). Housing after care: understanding security and stability in the transition out of care through the lenses of liminality, recognition and precarity. *Journal of Youth Studies, 26*(1), 80-97. <https://doi.org/10.1080/13676261.2021.1981838> | Recognition | Recognition theory (Honneth) indicates that people desire to be recognised as distinct and valued members of society, differentiated from others and groups, leading to self-recognition. Experiences of love and caring are key. Recognition is located in social relationships. The theory is used to make sense of housing. Receiving stable housing is a tangible message of caring recognition – housing is an extension of care. | 1 | Data collection was structured around the theoretical concepts | Policies are needed that permit care-leavers to remain in their placement or to live in time-secure (stable) housing. |
| Paulsen, V., & Thomas, N. (2018). The transition to adulthood from care as a struggle for recognition. *Child & Family Social Work, 23*(2), 163-170. <https://doi.org/10.1111/cfs.12395> | Recognition | The theory of recognition (Honneth) helped to understand and unfold the challenges for young people leaving state care and, in particular, how experiences of misrecognition can threaten the foundations of self‐confidence, self‐respect, and self‐esteem. Where these things were not present, the experience was of being unsupported and left to founder, or at best to flounder. (p. 164) | 1 | Theory is linked to findings. | Authors recommend that direction be provided to social work practice and shows how the transitional support needs to focus broadly on the youths’ life situation and have a holistic approach where youths feel cared for and loved in key helping relationships It also point at the need for a strength‐based approach. They also recommend as per the findings in this article the need for creating and maintaining supportive relations of consistency and continuity. |
| Driscoll, J. (2019). Strangers and estrangement: Young people’s renegotiations of birth and foster family relationships as they transition out of care and the implications for the state as parent. *Children’s Geographies, 17*(5), 539-551. <https://doi.org/10.1080/14733285.2017.1422209> | Relational autonomy | Advances the notion of ‘foundational rights’ which are necessary to enable a child to exercise full autonomy. The state is responsible to enable a child to acquire the capacity to exercise full autonomy when they reach adulthood. This is enacted through relational autonomy (Hollingsworth), in which important relationships create a safe context within which young people can learn to exercise autonomy. | 2 | Theory is present but does not strongly drive the study or illuminate findings | State needs to exercise its role as corporate parent to enable relational autonomy, to enable foundational rights. |
| Abdullah, A., Cudjoe, E., Emery, C. R., & Frederico, M. (2020). Moving towards independent living in Ghana: Narratives from young adults about their kinship care experience. *Journal of Adolescence, 79*, 148-156. <https://doi.org/10.1016/j.adolescence.2020.01.005> | Resilience | Resilience (Luthar et al.) emerges as a theme in the study findings – development of resilience strategies. The theme addresses participants’ conception of resilience (surviving, mentality, forging ahead). Resilience is used as a concept, but not theorised. It appears in the discussion also. | 3 | Theory used post-hoc to explain findings | Authors suggest that kinship care develops resilience, though acknowledges that how this happens is not known. No clear recommendations are made for practice. |
| Adley, N., & Jupp Kina, V. (2017). Getting behind the closed door of care leavers: understanding the role of emotional support for young people leaving care. *Child & Family Social Work, 22*(1), 97-105. <https://doi.org/10.1111/cfs.12203> | Resilience | Resilience (Stein) as a theory concerning “the quality that enables some young people to find fulfilment in their lives despite their disadvantaged backgrounds, the problems or adversity they may have undergone or the pressure they may experience. Resilience is about overcoming the odds, coping and recovery.” | 3 | Mentioned up front but not used again in the study. | No theory-informed practice recommendations provided. |
| Artamonova, A., Guerreiro, M. d. D., & Höjer, I. (2020). Time and context shaping the transition from out-of-home care to adulthood in Portugal. *Children and Youth Services Review, 115*, 105105. <https://doi.org/10.1016/j.childyouth.2020.105105> | Resilience | Resilience (Rutter) enabled participants to navigate through adversities and overcome difficulties. | 1 | Theory informed both data collection and analysis | Men tend to be more resilient than women, because of the gendered structuring of society – different exposures to adversity and opportunities. |
| Bengtsson, M., Sjöblom, Y., & Öberg, P. (2018a). ‘Well, it’s up to me now’: Young care leavers’ strategies for handling adversities when leaving out-of-home care in Sweden. *Nordic Social Work Research, 8*(sup1), 8-18. <https://doi.org/10.1080/2156857X.2018.1428673> | Resilience | An ecological resilience perspective is adopted, with particular use of Ungar’s navigation (locating resources) and negotiation (leveraging the resources) themes, i.e. emphasizing agency within social ecologies. Findings show how care-leavers reframe inner experiences and build self-reliance, while also restructuring their social network | 1 | Explicit theoretical framework informs the study | Because the study is small, no practice recommendations are provided, other than a few lines that transitions out of care should be gradual and support prolonged. |
| Berridge, D. (2017). Driving outcomes: Learning to drive, resilience and young people living in residential care. *Child & Family Social Work, 22*(1), 77-85. <https://doi.org/10.1111/cfs.12198> | Resilience | Driving is stated to be a resilience (Rutter) resource that enables better outcomes despite the adversities related to growing up in care. However, resilience theory is presented in isolation from driving – thus little framing of how driving can constitute a resilience resource. Study found that driving boosted self-esteem and self-confidence and strengthened relational bonds (attachment) with care staff. | 2 | Extensive discussion of resilience theory up front, but not strongly linked to the topic | Suggest that driving lessons may be a useful component of care-leaving programmes, providing multiple benefits to care-leavers. |
| Bodiroa, A., & Ross, E. (2023). Challenges, agency and ecology in the transition of youth from state residential care to independent living in Johannesburg, South Africa. *Child & Youth Services, 44*(3), 328-354. <https://doi.org/10.1080/0145935X.2022.2113871> | Resilience | Resilience (Pinkerton & Van Breda) is framed as being located in the interactions between people and their environment. Findings are that individual resilience factors outweigh environmental, and that most of the environment is antithetical to successful transitions from care. | 1 | Social ecological resilience shaped the study question and analysis | More needs to be done to foster nurturing social environments (both relational and structural) that facilitate resilience in care-leavers. |
| Bogdanova, E. (2017). Russian SOS Children’s Villages and deinstitutionalisation reform: Balancing between institutional and family care. *Zhurnal Issledovanii Sotsialnoi Politiki [The Journal of Social Policy Studies], 15*(3), 395-406. <https://doi.org/10.17323/727-0634-2017-15-3-395-406> | Resilience | Resilience theory (Walsh) is used to show that SOS Children’s Villages avoid the pitfalls of institutional care, by maintaining family-like care settings. Negative impacts of being placed in care can be offset with resilience resources, e.g., a stable caring adult and reintegration into a caring family. | 2 | Study is informed by theory but its place is not central | Recommend that SOS Children’s Villages are aligned with this theory and should not be closed down or restructured under the deinstitutionalisation agenda |
| Bond, S., & van Breda, A. D. (2018). Interaction between possible selves and the resilience of care-leavers in South Africa. *Children and Youth Services Review, 94*, 88-95. <https://doi.org/10.1016/j.childyouth.2018.09.014> | Resilience | Resilience theory (Van Breda) is interested in factors that enable better-than-expected outcomes in the wake of adversity. A future orientation (possible self) is argued to be an important enabler. Moreover, other resilience enablers can help to develop a care-leaver’s possible self. | 1 | Study sets out of examine this theory’s contribution to preparation for care-leaving | Recommend that resilience-building interventions include a focus on developing possible selves. |
| Crous, G., Montserrat, C., Gallart-Mir, J., & Matás, M. (2021). ‘In the end you’re no longer the kid from the children’s home, you’re just yourself’: Resilience in care leavers over 25. *European Journal of Social Work, 24*(5), 896-909. <https://doi.org/10.1080/13691457.2021.1918063> | Resilience | This study is informed by resilience theory (Rutter). Personal resilience (ability to deal with adverse situations and perceived control and autonomy) and relational and environment resilience (education and social support) were important enablers of leaving care. The passage of time also helped reduce a focus on being a ‘care-leaver’. | 1 | Study questions shaped around the theory and findings interpreted in relation to the theory | Authors recommend that the resilience strategies they found should be promoted among care-leavers. No specific practice implications articulated. |
| Dima, G., & Bucuta, M. D. (2015). The process of transition from public care to independent living: A resilience-based approach. *Revista de Cercetare si Interventie Sociala, 50*, 53-65. <http://www.rcis.ro/images/documente/rcis50_04.pdf> | Resilience | Describes a programme to facilitate successful care-leaving. | 2 | Resilience theory present, but not clear how these shaped the study | Recommend a resilience-informed programme focused on successful transitions. |
| Disney, T., & Walker, C. (2023). Young people leaving care and institutionalised vulnerability in the Russian Federation. *Children & Youth Services Review, 155*, 107225. <https://doi.org/10.1016/j.childyouth.2023.107225> | Resilience | In literature review, resilience (Stein) used to explain ‘poor outcomes’ or vulnerability among care-leavers. | 3 | Theory mentioned only in introductory pages. | No theory-informed practice recommendations provided. |
| Frimpong-Manso, K. A. (2018). Building and utilising resilience: The challenges and coping mechanisms of care leavers in Ghana. *Children and Youth Services Review, 87*, 52-59. <https://doi.org/10.1016/j.childyouth.2018.02.016> | Resilience | Resilience theory (Rutter) informs coping resources among care-leavers, including informal social supports and self-reliance. | 2 | Theory informs the study, but doesn’t go beyond coping mechanisms | Build in opportunities for youth to form community connections prior to leaving care. |
| Frimpong-Manso, K. A. (2020). Stories of care-leaving: The experiences of a group of resilient young adults on their journey to interdependent living in Ghana. *Emerging Adulthood, 8*(1), 16-25. <https://doi.org/10.1177/2167696818807114> | Resilience | Resilience theory (Rutter) used to identify resilience enablers from a sample of ‘successful’ care-leaving: positive relationships, preparation for adulthood, social support and personal capacities. | 2 | Theory informs the study, but doesn’t clearly drive it | Promote a meaningful (informal) relationship with at least one caregiver, build care-leavers’ relationship skills and interdependent living skills, extend age of leaving care to 21. |
| Furey, R., & Harris-Evans, J. (2021). Work and resilience: Care leavers’ experiences of navigating towards employment and independence. *Child & Family Social Work, 26*(3), 404-414. <https://doi.org/10.1111/cfs.12822> | Resilience | Resilience theory (Ungar) was found to be located at the interaction between an enabling environment and emotionally supportive networks. The latter (relationships) is more important than the former (tangible resources). | 1 | Study constructed around resilience theory | Emotional support emerges as central to successful care-leaving, more than tangible resources. Even mentoring relationships (internships and apprenticeships) should focus more on emotional support. |
| Greeson, J. K. P., & Thompson, A. E. (2017). Development, feasibility, and piloting of a novel natural mentoring intervention for older youth in foster care. *Journal of Social Service Research, 43*(2), 205-222. <https://doi.org/10.1080/01488376.2016.1248269> | Resilience | Natural mentoring promotes resilience (Thomspon et al.). Authors developed a conceptual framework for a natural mentoring programme, in which the 12-week C.A.R.E. programme contributes to a growth-fostering relationship with a natural mentor, which contributes non-cognitive abilities (grit, resilience, affect regulation and future orientation), prosocial developmental outcomes and reduced health risk behaviours, which in turn lead to better life course outcomes. | 1 | Developed a theory of change that incorporated resilience | Recommend the implementation of their programme. |
| Häggman-Laitila, A., Salokekkilä, P., Satka, M., Toivonen, K., Kekolahti, P., & Ryynänen, O.-P. (2019). The coping of young Finnish adults after out-of-home care and aftercare services: A document-based analysis. *Children and Youth Services Review, 102*, 150-157. <https://doi.org/10.1016/j.childyouth.2019.05.009> | Resilience | Resilience theory (Stein) is used to understand individual development holistically, with a focus on strengths. The ‘moving on’ group of Stein’s typology as considered ‘resilient’, comprising 19% of the sample of 600 care-leavers. | 3 | Resilience mentioned only briefly | No theory-informed practice recommendations provided. |
| Hamilton, D. J., Taylor, B. J., Killick, C., & Bickerstaff, D. (2015). Suicidal Ideation and behaviour among young people leaving care: Case-file survey. *Child Care in Practice, 21*(2), 160-176. <https://doi.org/10.1080/13575279.2014.994475> | Resilience | Resilience (Daniel & Wassell) is important for overcoming life’s challenges, and is rooted in having a ‘secure base’ (i.e. attachment). A good quality placement can generate a secure bases, while educational engagement can contribute to resilience. | 3 | Theory just briefly mentioned in the literature review | No theory-informed practice recommendations provided. |
| Heyman, J. C., White-Ryan, L., Kelly, P., Farmer, G. L., Leaman, T. L., & Davis, H. J. (2020). Voices about foster care: The value of trust. *Children and Youth Services Review, 113*, 104991. <https://doi.org/10.1016/j.childyouth.2020.104991> | Resilience | Interested in the factors that might protect CL from homelessness. Resilience theory (Stein) provides insights into risk factors and protective factors that shape a care-leaver’s transition and outcomes. Attachment is a protective factor. Findings highlight independence/autonomy and recognising strengths as important for care-leaving. | 3 | Resilience theory introduced but not used in study | ?? |
| Hlungwani, J., & van Breda, A. D. (2020). Female care leavers’ journey to young adulthood from residential care in South Africa: Gender‐specific psychosocial processes of resilience. *Child & Family Social Work, 25*(4), 915-923. <https://doi.org/10.1111/cfs.12776> | Resilience | Resilience (Van Breda) is the social-ecological capacity to recover from adversity. Care-leaving resilience processes, identified in a previous study with men, were replicated with women. Previous results were confirmed and two additional female-specific processes were identified. | 1 | Resilience theory drove the study | The care-leaving processes found should be fostered among children in care, to facilitate resilient outcomes as they transition from care. |
| Hlungwani, J., & van Breda, A. D. (2022). Affording managed opportunities for independence to build looked-after young people’s resilience: Perceptions and experiences of care workers. *Journal of Children’s Services, 17*(2), 137-151. <https://doi.org/10.1108/JCS-10-2021-0044> | Resilience | Managed opportunities for independence is a practice approach located within a multisystemic resilience theory framework (van Breda). A resilience approach is adopted. The opportunities to exercise independence, with managed monitoring, enables children in care to practice for the independence they will experience after leaving care, thus building resilience and promoting resilient outcomes. | 1 | Resilience theory drives the study | Policies and procedures for promoting managed opportunities for independence should be formulated and implemented. |
| Isakov, A. B., & Hrnčić, J. (2018). Preparedness for emancipation of youth leaving alternative care in Serbia. *International Journal of Child, Youth & Family Studies, 9*(1), 83-107. <https://doi.org/10.18357/ijcyfs91201818121> | Resilience | Resilience (Stein) is understood as better care-leaving outcomes and is a hoped-for result of this study. | 2 | Resilience concepts are used to make sense of the data | Promote physical and mental health to improve resilient outcomes. Help care-leavers construct themselves as ‘successful’ to improve outcomes. |
| Kelly, P., Heyman, J., Zhai, F., & Salazar, A. (2023). Social and emotional supports during college years: Associations with post-college outcomes among alumni of foster care. *Child and Adolescent Social Work Journal, 40*(5), 607-621. <https://doi.org/10.1007/s10560-021-00806-4> | Resilience | Resilience (Greene) is constructed as the ability to overcome, cope with or recover from adversity. It includes systemic factors, such as the social environment and relational networks. Resilience theory is used to explain enablers for care-leavers after completion of tertiary education (post-college). Higher levels of social support and socialisation in college were associated with higher self-perceptions of happiness after college and lower use of public assistance. | 1 | Study was conducted to answer theoretically informed questions | Campus support programmes should be offered to care-leavers to facilitate transition into college, build relational networks, and graduate. Programmes should help care-leavers in college build meaningful and sustainable social networks. Social supports formed during college should be continued after graduation. Financial support for those in education should be extended beyond age 21. |
| Melkman, E. P., Refaeli, T., Bibi, B., & Benbenishty, R. (2016). Readiness for independent living among youth on the verge of leaving juvenile correctional facilities. *International Journal of Offender Therapy and Comparative Criminology, 60*(10), 1209-1225. <https://doi.org/10.1177/0306624X15575117> | Resilience | The study examines self-reports of readiness for independent living among adolescents in correctional settings on the verge of leaving care, and explores how individual, placement, and social support characteristics and predict such perceived readiness. Term resilience is mentioned in the discussion section, and not a theoretical basis of the study. | 3 | Mentioned in 2 sections in the entire article. Very briefly mentioned in the literature review and discussion section | The key role of the support networks within the institutions is vital to include the renegotiation of familial relationships within re-entry planning, identifying and strengthening sources of support within the family or ensuring alternative ones when these are lacking. |
| Mendes, P. (2018). Towards the social inclusion of young people transitioning from out-of-home care: An examination of the Home Stretch campaign. *Social Alternatives, 37*(1), 59-62. <https://openjournals.library.sydney.edu.au/SWPS/article/view/12920/12020> | Resilience | Outcomes are influenced by “individual agency or resilience (within a social context)” (p. 60). Theory not cited. | 3 | Mentioned in passing | No theory-informed practice recommendations provided. |
| Mendes, P., Standfield, R., Saunders, B., McCurdy, S., Walsh, J., & Turnbull, L. (2021). Aboriginal and Torres Strait Islander (Indigenous) young people leaving out-of-home care in Australia: A national scoping study. *Children and Youth Services Review, 121*, 105848. <https://doi.org/10.1016/j.childyouth.2020.105848> | Resilience | This scoping study explores indigenous (Aboriginal and Torres Strait Islander) children in Australia. Resilience mentioned but not cited. | 3 | Resilience once in literature review, but not theorised | No theory-informed practice recommendations provided. |
| Mendis, K., Lehmann, J., & Gardner, F. (2018). Promoting academic success of children in care. *British Journal of Social Work, 48*(1), 106-123. <https://doi.org/10.1093/bjsw/bcx029> | Resilience | Resilience (constructed as a personal characteristic) (Gilligan; Daniel) can increase educational engagement | 3 | Theory briefly mentioned in discussion of one finding | Enrich the care environment by reducing risk factors and increasing protective factors |
| Modi, K., Kasana, S., Azam, A., & Madhavan, L. (2021). Education, poverty and social exclusion: Assessment of youth leaving care. *SN Social Sciences, 1*(4), 82. <https://doi.org/10.1007/s43545-021-00102-z> | Resilience | This study examines the interrelatedness of education, poverty and social exclusion among care-leavers through secondary literature and empirical data from Udayan Care’s national study. Not clear how resilience (Stein) was used; just mentioned as the tenacity and resilience of care-leavers instil much hope in their being able to soar if this structural neglect of care-leavers is addressed and they are supported to achieve to their goals. | 3 | Mentioned twice in the article: once in literature review and once in the discussion section. | No theory-informed practice recommendations provided. |
| Modi, K., Prasad, A., & Mishra, J. (2018). Assisting youth leaving care: Understanding Udayan Care’s aftercare programme through the prism of ecological systems theory. *Scottish Journal of Residential Child Care, 17*(1), 1-24. <https://doi.org/10.17868/strath.00084531> | Resilience | Resilience (Masten) was explored in relation to mastery, relatedness and emotionality. Data on these measures suggest many care-leavers have high resilience | 1 | Study driven by the theory | Long-term caring relationships and personal resilience are important to foster in care. |
| Moore, J., Flynn, M., & Morgan, M. (2019). Social ecological resilience and mental wellbeing of Irish emigrant survivors of clerical institutional childhood abuse. *Child Abuse Review, 28*(1), 52-68. <https://doi.org/10.1002/car.2548> | Resilience | Resilience resources (Ungar) including personal resources, “such as problem-focused coping, altruism and defiance” as well as community and social inclusion. For Irish emigrant survivors compared to Irish survivors who remained in Ireland, a social identity not defined by abuse and instrumental support also facilitated mental well-being. | 1 | Study driven by theoretical framework | Relocating to a new country or area after a history of institutional child abuse may help to open up new ways of constructing one’s identity (other than as a survivor of abuse). |
| Moore, J., Thornton, C., & Hughes, M. (2017). On the road to resilience: The help-seeking experiences of Irish emigrant survivors of institutional abuse. *Child Abuse Review, 26*(5), 375-387. <https://doi.org/10.1002/car.2415> | Resilience | An important resilience resource (Ungar) for abuse survivors is therapeutic intervention. After negative experiences of seeking help, survivors achieved better outcomes through self-management and only disclosed abuse much later in life. Turning points (e.g., illness, death or having children) and peer support facilitated help seeking. | 1 | Study driven by theoretical framework | Greater awareness among practitioners about institutional child sexual abuse and willingness to engage will facilitate disclosure by survivors. Practitioners need to recognise atypical coping methods as valid resilience enablers for survivors. Promotion of peer support networks similarly facilitates help seeking. |
| Mupaku, W. M., van Breda, A. D., & Kelly, B. (2021). Transitioning to adulthood from residential childcare during COVID-19: Experiences of young people with intellectual disabilities and/or autism spectrum disorder in South Africa. *British Journal of Learning Disabilities, 49*(3), 341-351. <https://doi.org/10.1111/bld.12409> | Resilience | This study focuses on young people with intellectual disabilities and/or autism who, due to child welfare concerns, have grown up in children’s residential care and are now transitioning out of care at the age of 18 years towards young adulthood. COVID-19 lockdown cut participants off from family, friends, work and education, restricting their social-relational support systems. Thus, with this came a reduction of resilience (Ungar), and, thus, deteriorations in behaviour and mental health, with a suicide attempt. | 2 | Resilience mentioned in the Discussion and Conclusion section of the article | Recommendation was made that support services continue during lockdown, while adhering to COVID-19 regulations, to facilitate resilience and well-being among care-leavers, caregivers, and other members of the household. |
| Neal, D. (2017). Academic resilience and caring adults: The experiences of former foster youth. *Children and Youth Services Review, 79*, 242-248. <https://doi.org/10.1016/j.childyouth.2017.06.005> | Resilience | Academic resilience (Morales) is a construct tied to positive anomalies in educational outcomes for students labeled as at-risk. The theory is that aspirations and academic success represent highly meaningful accomplishments for youth who must transcend a multitude of barriers to attain them; these youth are considered academically resilient. | 1 | Resilience is mentioned throughout the study and the analysis is made drawing on the theory. | Authors recommend that researchers and practitioners, should be careful not to over rely on theories such as resilience for solutions that place unfair burdens on young, particularly for populations of young people who are experiencing structural and social inequities. |
| Nho, C. R., Park, E. H., & McCarthy, M. L. (2017). Case studies of successful transition from out-of-home placement to young adulthood in Korea. *Children and Youth Services Review, 79*, 315-324. <https://doi.org/10.1016/j.childyouth.2017.06.035> | Resilience | The study adopts a resilience model (Stein) and examines successful transitions of children from out-of-home care to young adulthood in Korea to draw implications for child welfare practice and policies. The purpose of this study was to identify factors that enabled young adults to successfully move to living independently. In the findings, some of the outcomes were linked to resilience that participants in this study presented a variety of individual factors that helped them succeed in young adulthood, including trusting in themselves rather than their environments, not being held back by their pasts, positive power, and the spirit of “I don’t give up.” These factors also illustrate resilience and an inner strength that participants displayed (p. 320). | 1 | Links theory to the findings. | Authors recommend that future research may be able to outline the core conditions that support or help develop resilience so that further implications for child welfare practice and policy can be drawn. |
| Olsson, T. M., Blakeslee, J., Bergström, M., & Skoog, T. (2020). Exploring fit for the cultural adaptation of a self-determination model for youth transitioning from out-of-home care: A comparison of a sample of Swedish youth with two samples of American youth in out-of-home care. *Children and Youth Services Review, 119*, 105484. <https://doi.org/10.1016/j.childyouth.2020.105484> | Resilience | The purpose of this study was to explore the fit of a self-determination model (i.e., My Life) for youth transitioning from out-of-home care for the Swedish context by comparing baseline characteristics of two American samples of adolescents in out-of-home care who have participated in and benefited from experimental tests of the self-determination model with a sample of adolescents in care in Sweden. The short version of the Resilience Scale was used (Wagnild & Young). | 3 | Mentioned twice in the findings and discussion section | No theory-informed practice recommendations provided. |
| Pinkney, S., & Walker, G. (2020). ‘It was me, but it was them that helped me’: Exploring the issues for care experienced young people within higher education. *Children and Youth Services Review, 108*, 104576. <https://doi.org/10.1016/j.childyouth.2019.104576> | Resilience | This study reinforces the importance of stable and secure loving relationships which helps to build resilience (Ungar), and that emotional issues need to be supported before children in care can make good use of educational opportunities. Resilience and the personal attributes of young people are of increasing interest to researchers. | 2 | Does not mention resilience as a theory but a term. Mentions made in the literature section to the social ecology of resilience (p. 4) links resilience to some findings | Building of social support systems assist in building resilience and academic success. |
| Rácz, A. (2018). Alternative care of children without family in Hungary. *Revista de Asistenta Sociala, 3*, 23-32. <http://www.swreview.ro/index.pl/alternative_care_of_children_without_family_in_hungary> | Resilience | Drawing on resilience theory (not cited), this study explores how the system helps “in the processing of traumas experienced by children within their family and when taken away from their family” to become “successful adults”. | 3 | Used the term twice resilience, with no citations, and no links to the findings. | No theory-informed practice recommendations provided. |
| Radey, M., Boel-Studt, S., & Collins, C. J. (2023). “Taking it day by day”: Maternity group homes in the lives of mothers aging out of foster care. *Children and Youth Services Review, 155*, 107293. <https://doi.org/10.1016/j.childyouth.2023.107293> | Resilience | This study aimed to understand maternity home characteristics, strengths, and challenges from mothers’ and providers’ perspectives. Resilience theory (Stein) offers a conceptual framework through which to consider the potential power of maternity homes in the lives of young pregnant and parenting teens aging out of foster care. | 2 | Mentioned in literature review and discussion | No theory-informed practice recommendations provided. |
| Refaeli, T. (2017). Narratives of care leavers: What promotes resilience in transitions to independent lives? *Children and Youth Services Review, 79*, 1-9. <https://doi.org/10.1016/j.childyouth.2017.05.023> | Resilience | The resilience (Stein) of care-leavers is examined, looking at three types of protective factors, viz. personal resources, support from family, and support from significant others. The study explores this aspect of resiliency by analysing the stories of care-leavers who were transitioning from military service to independent life. | 1 | The entire study is based on the use of the resilience theory. It is mentioned in the literature review, links are also made in the findings section. | An important intervention from the study should be to maintain the social relationships that care-leavers established while in care and to assist them establish new ones after care. To provide care-leavers with the skills they will need to establish different kinds of relationships. |
| Samarah, E. M. S., Schelbe, L., & Jackson, L. A. (2023). A photovoice study of college students who have experienced foster care, relative care, and/or homelessness. *Children and Youth Services Review, 151*, 107042. <https://doi.org/10.1016/j.childyouth.2023.107042> | Resilience | Unlike traditional conceptualizations of resilience (Luthar et al.) that suggest resilience as a largely individualistic process predicated on exposure to adversity and achievement via positive adaptation, “unconquered” was related to scholars becoming involved in a community, seeking support, and advocating for self and others. | 2 | Mentioned in the findings and discussion sections | No theory-informed practice recommendations provided. |
| Sekibo, B. (2020). Experiences of young people early in the transition from residential care in Lagos State, Nigeria. *Emerging Adulthood, 8*(1), 92–100. <https://doi.org/10.1177/2167696818822232> | Resilience | This article draws on resilience theory (Van Bred) to examine the aftercare experiences of young people who had recently left residential care in Nigeria. Findings show that care-leavers were filled with resilient optimism, in terms of personal and social factors. Care-leavers in the study showed a willingness to want to change and get back on the right path in the hope of better outcomes through persistence was greatly fuelled by their fear of failing, which kept them from settling for less; envisaging a future for themselves translated into resilience. | 1 | Entire study was based on the resilience theory. | No theory-informed practice recommendations provided. |
| Simpson, D., & Murphy, S. F. (2022). ‘So we beat on, boats against the current, borne back ceaselessly into the past’: Legacy, care leavers and university study. *Journal of Youth Studies, 25*(2), 259-274. <https://doi.org/10.1080/13676261.2020.1865527> | Resilience | The aim of the study was to capture information about care-leavers’ perceptions and experiences of higher education to add to understanding and insight in these areas. Resilience theory (Stein) included an interactional process, which helped them cope with adversity and trauma from the past. | 2 | Theory was used only in the findings section | No theory-informed practice recommendations provided. |
| Sims-Schouten, W., & Hayden, C. (2017). Mental health and wellbeing of care leavers: Making sense of their perspectives. *Child & Family Social Work, 22*(4), 1480-1487. <https://doi.org/10.1111/cfs.12370> | Resilience | The concept resilience (Stein) was used a few times to describe the preferred state of being of participants. | 3 | Mentioned in passing in literature review, findings and conclusion | “Leaving care projects need to have a more skilled and targeted approach to addressing mental health and wellbeing issues and building resilience.” |
| Solem, M.-B., Helgeland, I. M., Brannen, J., & Phoenix, A. (2020). Transitions to adulthood of ‘at risk’ young men: New analysis from two Norwegian qualitative longitudinal studies. *Children & Society, 34*(3), 220-233. <https://doi.org/10.1111/chso.12369> | Resilience | The study explores how male care-leavers negotiated the transition to adulthood and exercised agency. It identifies the ways in which supportive social relationships help to produce everyday practices that develop resilience and create positive turning points and potentially happy and successful futures. The concept of ‘social resilience’ (Hall & Lamont) is useful for understanding the relationship between social change and the life course. | 3 | Mentioned in introduction and conclusion | No theory-informed practice recommendations provided. |
| Spallek, M., & Haynes, M. (2021). Post-school transitions for children in out-of-home care: Evidence from the Australian Census Longitudinal Dataset. *Journal of Youth Studies, 24*(7), 901-924. <https://doi.org/10.1080/13676261.2020.1781803> | Resilience | Resilience theory (Fergus & Zimmerman) was said to complement the life course theory and provides a conceptual framework for studying and understanding why some children grow up to be ‘normal’ adults despite risks exposure. The link to resilience theory emphasises the importance of promotive factors that counteract risk factors, enabling children to mitigate the negative impacts of risk exposure. | 2 | Theory influences data collection and analysis | The focus should be on the modifiable promotive or resilience factors that were identified and related to the family living environment such as number of children in the household, education and employment status of parents and residential area. |
| Stein, M. (2019). Supporting young people from care to adulthood: International practice. *Child & Family Social Work, 24*(3), 400-405. <https://doi.org/10.1111/cfs.12473> | Resilience | This study explores practice examples, of peer research carried out in 11 countries relating to young people’s transitions from care to adulthood. Practice examples are drawn from young people’s pathways to adulthood, leaving care law and policy and participation. Resilience (Masten) is used as a basis of the analysis and it is argued that resilience is key for positive care-leaving outcomes, such as educational success and mental health. | 1 | Study draws on the resilience as a framework consistently through literature and analysis. | Author mentions the various practice programmes in the sectors of foster care, residential and kinship care and best policies in the various research that was looked at. He suggests that resilience provides but one approach to interrogating the leaving care practice examples that were provided. |
| Stephens, T., & Aparicio, E. M. (2017). “It’s just broken branches”: Child welfare-affected mothers’ dual experiences of insecurity and striving for resilience in the aftermath of complex trauma and familial substance abuse. *Children and Youth Services Review, 73*, 248-256. <https://doi.org/https://doi.org/10.1016/j.childyouth.2016.11.035> | Resilience | The study explores the life stories of American women of colour with a foster care history. The mothers report multifaceted trauma and substance abuse within their families, and relational, housing and financial insecurity. The study is shaped by resilience theory (not cited). Resilience was expressed through perseverance, faith, empathy, and establishing stable homes. | 1 | Resilience, though not theorised, is used a basis for the study from literature section through to analysis. | Authors recommend trauma-informed, comprehensive behavioural health services together with stable housing, financial and social support building (including faith-based communities), and parenting support. To improve children’s well-being, it is crucial to acknowledge that mothers may emerge from substance abuse cycles and develop a new, resilient path. |
| Sulimani-Aidan, Y. (2017a). Future expectations as a source of resilience among young people leaving care. *British Journal of Social Work, 47*(4), 1111–1127. <https://doi.org/10.1093/bjsw/bcw077> | Resilience | This study looks at various literature to examine the links between future prospects, resilience (Sulimani-Aidan) and outcomes, and focuses on two possible personal and environmental resources that can contribute to care-leavers’ positive future expectations: optimism and social support. It looks at possible correlations between future expectations with resilience, has little has been written about young people’s future expectations as a source of resilience. | 1 | Entire study is based on the resilience theory | Author recommends enhancing youth’s resilience by using interventions that teach cognitive–behavioural and social problem-solving skills and designing programmes that increase youth’s possibilities in different areas after leaving care such as financial independence and higher education could broaden their possibilities. |
| Sulimani-Aidan, Y. (2017b). ‘She was like a mother and a father to me’: searching for the ideal mentor for youth in care. *Child & Family Social Work, 22*(2), 862-870. <https://doi.org/10.1111/cfs.12306> | Resilience | The goal of this study was to describe the profile of a staff member who was able to establish a meaningful relationship with youth in care through the perspectives of 20 young adults aged 21–26 who left care in Israel. Mentoring relationships are supported by resilience (Goldner & Mayseless) among at-risk children and youth. | 3 | Resilience mentioned in the introduction only, in terms of mentoring relationships and the connection to resilience studies. | No theory-informed practice recommendations provided. |
| Sulimani-Aidan, Y. (2018a). Assets and pathways in achieving future goals of residential care alumni. *Children and Youth Services Review, 89*, 71-76. <https://doi.org/10.1016/j.childyouth.2018.04.023> | Resilience | Resilience theory (Masten) is used as a theoretical framework during the analysis process of the study. This study adds to the existing literature on the factors that promote resilience among youth leaving care, specifically in relation to the young adults’ own hopes and plans for the future. | 2 | Resilience theory cascades throughout the entire paper. | Practitioners need to assist care-leavers in reducing self-reliance as a source of resilience and increasing interdependence and health relationships. |
| Sulimani-Aidan, Y. (2018c). Present, protective, and promotive: Mentors’ roles in the lives of young adults in residential care. *American Journal of Orthopsychiatry, 88*(1), 69-77. <https://doi.org/10.1037/ort0000235> | Resilience | Resilience as a concept is discussed in terms of the emerging adulthood safe of life of young people leaving care and their challenges experienced. This study adds to the existing literature on the factors that promote resilience among youth leaving care, specifically in relation to the young adults’ own hopes and plans for the future. | 2 | Only mentioned in introduction only. | Authors recommend strengthening of the informal and formal support to increase resilience. |
| Sulimani-Aidan, Y. (2018d). Promoting resilience among adolescents in care from their social workers’ perspectives. *Children and Youth Services Review, 94*, 43-48. <https://doi.org/10.1016/j.childyouth.2018.09.005> | Resilience | This study explored the perceptions of thirty social workers regarding resilience (Stein) among the adolescents they treat and their view of the factors that contribute to resilience and their perceptions regarding their role in promoting it amongst this group of young people. The aim of this study is to add to examine the factors that promote resilience among adolescents in care | 1 | Study is based on resilience theory and links are made to it through the study | Author recommends adding to the intervention programmes to strengthen young people’s outlook for the future and widen the social workers’ knowledge of the young person’s social context to promote resilience. |
| Sulimani-Aidan, Y. (2020b). Social networks during the transition to adulthood from the perspective of Israeli care leavers and their social workers. *Children and Youth Services Review, 115*, 105075. <https://doi.org/10.1016/j.childyouth.2020.105075> | Resilience | The study involved 50 young adults and their social workers regarding their views on the role of formal and informal social networks when leaving care. Social networks are central to young people’s resilience (Masten), however care-leavers have a mix of supportive and unsupportive relationships with family members, leaving no clear-cut association between resilience and family support. | 1 | Entire study is based on the resilience theory | Care-leavers need to continue to receive support from various formal figures in their lives who can assess their needs holistically and promote their daily independent living and future accomplishments. Professionals in the field need to acknowledge the importance of informal social ties as a resource. |
| Sulimani-Aidan, Y., Achdut, N., Zeira, A., & Benbenishty, R. (2022). Psychological distress among care leavers during the transition to adulthood: Risk and protective factors throughout their life course. *Child & Family Social Work, 27*(2), 324-339. <https://doi.org/10.1111/cfs.12887> | Resilience | One of the psychological characteristics, which focused on optimism as one of the traits that contributes to resilience. Optimism was negatively correlated with psychological distress. | 2 | The study’s findings also correspond with resilience theory, which appears in the discussion | Study findings draw on resilience theory, which emphasizes the importance of optimism for the promotion of positive development. |
| Syme, A., & Hill, M. (2017). Professionals’ perceptions of the rocky routes to successful outcomes for young people in a children’s residential school. *Child & Family Social Work, 22*(1), 185-194. <https://doi.org/10.1111/cfs.12216> | Resilience | This is an evaluation study of an independent children’s residential school in Scotland which provided an opportunity to analyse and compare the views of different kinds of staff about desirable outcomes and how successful outcomes could be achieved. The study focusses on Resilience (Stein), as an approach in the study. | 2 | Mention is made of resilience in the introduction and parts of the discussion when looking at outcome. | No theory-informed practice recommendations provided. |
| Tahkola, E.-M., Metsäpelto, R.-L., Ruohotie-Lyhty, M., & Poikkeus, A.-M. (2021). Coping strategies in life stories of young adults with foster care backgrounds. *European Journal of Social Work, 24*(5), 910-921. <https://doi.org/10.1080/13691457.2021.1954885> | Resilience | This study focuses on coping strategies identified in interviews with 18 young adults (between 18 and 32 years) with foster care background and living in Finland. Resilience (Masten) is mentioned as part of coping. | 3 | Resilience is mentioned twice in study, with no links to the theoretical framework. | The importance of giving voice to young foster care adults’ coping (resilience) strategies, and sensitising foster families and professionals in the field to these strategies to better understand and support children and youth who face adversities. |
| Turner, L., & Percy-Smith, B. (2020). Care leavers and children’s services: Exploring the utility of communities of practice in theorising transition. *British Journal of Social Work, 50*(6), 1758-1774. <https://doi.org/10.1093/bjsw/bcz108> | Resilience | Self-care and resilience (not cited) are situated in supportive communities. Resilience is not a personal asset. Resilience varies across contexts. | 1 | Resilience is central to the argument | No theory-informed practice recommendations provided. |
| Turner, R., Vira, E. G., Bergström, M., & Olsson, T. M. (2023). Cultural adaptation of interventions and the a priori assessment of intervention fit: Exploring measurement invariance for American and Swedish youth leaving care. *Journal of Evidence-Based Social Work, 20*(3), 306-322. <https://doi.org/10.1080/26408066.2022.2159602> | Resilience | The study mentions to support youth transitioning from care, interventions focus on key constructs such as building resilience and self-efficacy. The term resilience is used to measure the transition, with the resilience scale being a tool (Wagnild & Young). | 1 | Study design, findings and discussion was based on the theory. | No theory-informed practice recommendations provided. |
| Van Breda, A. D. (2015). Journey towards independent living: A grounded theory investigation of leaving the care of Girls & Boys Town South Africa. *Journal of Youth Studies, 18*(3), 322-337. <https://doi.org/10.1080/13676261.2014.963534> | Resilience | This is a grounded study which identifies the processes that young people go through as they make the transition. Working from a resilience viewpoint, with an ecological emphasis, four central psychosocial processes arose that together explain the care-leaving experiences of the participants. | 1 | The theory is used to identify the psychosocial process of resilience. Links are made to the processes and the resilient theory. | The authors recommends that independent living programmes be established to assist youth differentiate authentic belonging from others by learning the social skills and processes for establishing strong ties. Therefore, programme development needs to gain understanding into the sources of these skills. Further research is also required on the social processes. |
| Van Breda, A. D. (2017). The Youth Ecological-Resilience Scale: A partial validation. *Research on Social Work Practice, 27*(2), 248-257. <https://doi.org/10.1177/1049731516651731> | Resilience | The purpose of this study is to provide a description and explanation of the design and validation of a new South African scale, called the Youth Ecological-Resilience Scale (YERS), which measures a range of resilience factors for with young people leaving the care system. Resilience theory, which has its focus on the ecological perspective provides a theoretical background within which the scale was designed. | 1 | Study is rooted in resilience theory. | Authors recommend that the YERS may prompt the design and provision of social work interventions, particularly those that are aligned with resilience theory, such as being guided by the strengths perspective and interventions to strengthen a young person’s resilience while in care by pre and post intervention. YERS is a tool that is rooted in an ecological theory of resilience that can be used in clinical practice. |
| Van Breda, A. D. (2018). The first two years out of residential care in South Africa: A critical period for care-leaving services. *Child Welfare, 95*(6), 63-82. <https://www.jstor.org/stable/48623607> | Resilience | This study looks at the changes in transitional or independent living outcomes of a group of residential care-leavers over their first and second years out of care. Data is drawn from a larger longitudinal study on care-leaving called Growth Beyond the Town. This article describes the changes, or lack of changes, in a range of independent living outcomes from one to two years out of care. | 3 | Resilience is mentioned only regarding the resilience assessment of young people which is a part of the study. | Author recommends thorough preparation for care-leavers is vital and this needs to be comprehensive and specific. Care-leavers require aftercare support during the first several months of being out of care to actualize their plan and services during the critical period of the first several months out of care are imperative. |
| Van Breda, A. D., & Dickens, L. F. (2017). The contribution of resilience to one-year independent living outcomes of care-leavers in South Africa. *Children and Youth Services Review, 83*, 264-273. <https://doi.org/10.1016/j.childyouth.2017.11.009> | Resilience | This study makes use of resilience (Ungar). This article draws on data from the only longitudinal study on care-leaving in South Africa. It uses resilience theory to explain the differences observed in independent living outcomes of care-leavers, one year after leaving the residential care of Girls and Boys Town and supports a social-ecological view of resilience. | 1 | Study is firmly embedded within the resilience theory | Authors recommends the importance of helping young people preparing to leave care by establishing connections back home so that by the time they transition the young person has an established support network. Also to encourage the teaching of social skills and need to address structural issues in society at large, those communities to which young people return to upon exit. |
| Van Breda, A. D., & Hlungwani, J. (2019). Journey towards independent living: Resilience processes of women leaving residential care in South Africa. *Journal of Youth Studies, 22*(5), 604-622. <https://doi.org/10.1080/13676261.2018.1523541> | Resilience | This study focuses on the resilience processes (van Breda) that facilitate better transitions of young women out of care and towards independent living. The aim of the study was to determine to what extent the resilience processes of young women are similar to those of men. | 1 | The study is embedded within the resilience theory. | “The authors conclude that resilience-building programmes can be implemented to prepare both males and females for leaving care” (p. 619). Practitioners working with young people leaving care can do similar work with both boys and girls in developing their capacity to apply these social processes during their transition from care. |
| Webb, L., Cox, N., Cumbers, H., Martikke, S., Gedzielewski, E., & Duale, M. (2017). Personal resilience and identity capital among young people leaving care: enhancing identity formation and life chances through involvement in volunteering and social action. *Journal of Youth Studies, 20*(7), 889-903. <https://doi.org/10.1080/13676261.2016.1273519> | Resilience | This study explores personal resilience (Ungar), in the form of self-esteem (confidence), ego strength (integrity and sense of purpose) and self-determination (agency) is believed to also be dependent on individualisation opportunities and identity capital among care-leavers and young people in care engaging in social activities through volunteering (p. 345). | 3 | The concept of resilience is used a few times in analysis, but not in depth in the study. | “Measured graduation towards individualisation appears to need continued support during exposure to opportunities for safe exploration; replicating graduation from the parental home. Examining the role of supported volunteering for care leavers found these exposures provide opportunities for the individualisation process, and at different stages of maturity” (p. 900). |
| Wilke, N. G., Roberts, M., Mitchell, T., & Howard, A. H. (2023). Spirituality as a protective factor: A multinational study of 267 adults separated from parental care as children. *Social Work and Christianity, 50*(2), 191-212. <https://doi.org/10.3403/swc.v50i2.309> | Resilience | The study explored the specific ways spirituality serves as a protective factor in a multinational sample of 267 individuals with care experience. Within resilience theory (Lee et al.), spirituality was identified as one protective factor. | 1 | Resilience discussed in literature review and mentioned in findings | The promotion of spirituality, as an instance of resilience, is recommended to affirm the role of spirituality, offer access to spirituality-related practices, consider spiritual communities as a source of support and provide opportunities to serve. |
| Neal, D. (2017). Academic resilience and caring adults: The experiences of former foster youth. *Children and Youth Services Review, 79*, 242-248. <https://doi.org/10.1016/j.childyouth.2017.06.005> | Resistance | Resistance was an important aspect of students’ lives that advanced their academic achievement and college aspirations as they found ways to resist their environments, prove people wrong, and avoid ending up like their biological parents. The theory of resistance (Solorzano & Delgado Bernal) was not a domain covered by the survey or interviews conducted for this research. However, students repeatedly spoke of resistance as a way to achieve academically and overcome life challenges. | 3 | Mentioned twice in article. Findings sections only | No theory-informed practice recommendations provided. |
| Mertz, M., & Andersen, S. H. (2017). The hidden cost of foster-care: new evidence on the inter-generational transmission of foster-care experiences. *British Journal of Social Work, 47*(5), 1377-1393. <https://doi.org/10.1093/bjsw/bcw132> | Resources | The lack of available support from their parents, may lead foster-care-leavers short on personal and social resources, which can facilitate intergenerational transmission of foster care experiences (Andersen & Fallesen). This emphasises coping skill and resource accessibility route. This study does find that lack of resources accounts for a fair amount, but not all, of the intergenerational transmission. | 1 | Study driven by theory | No theory-informed practice recommendations provided. |
| Raithel, J., Yates, M., Dworsky, A., Schretzman, M., & Welshimer, W. (2015). Partnering to leverage multiple data sources: Preliminary findings from a supportive housing impact study. *Child Welfare, 94*(1), 73-85. <https://www.jstor.org/stable/48623505> | Sanctuary Model | “The Sanctuary Model is a comprehensive approach to developing a trauma-sensitive culture in which psychological and social trauma can be addressed and resolved” (p. 75). It is based on the trauma theory. The model is not cited, but more information can be found at <https://www.thesanctuaryinstitute.org/>. | 3 | Mentioned twice in the study. | No theory-informed practice recommendations provided. |
| Luyten, I., Nuytiens, A., Christiaens, J., & Dumortier, E. (2018). Voicing young and older adult care-leavers in Belgium: How the experience of being in care shapes narratives of the self. *Longitudinal & Life Course Studies, 9*(1), 80-100. <https://doi.org/10.14301/llcs.v9i1.456> | Self as social construction | Study explores the construction of self in relation to care experience and as shifting over time, using life history interviews. Three selves emerge: collective, problematic and resilient selves. | 1 | Study driven by theory | Greater attention should be given to the internal and subjective experiences and processes of youth in care. |
| Bramsen, I., Kuiper, C., Willemse, K., & Cardol, M. (2019). My path towards Living on my own: Voices of youth leaving Dutch secure residential care. *Child & Adolescent Social Work Journal, 36*(4), 365-380. <https://doi.org/10.1007/s10560-018-0564-2> | Self-determination | Self-determination theory (Ryan & Deci) emphasises relational autonomy and volition – making one’s own choices. The study finds that autonomy emerged when care-leavers feel socially connected, i.e. relational autonomy. Youth express it as ‘doing your own thing’ in which they behaviour is congruent with their own internal value systems. Youth need time to rest and think. | 1 | Study driven by interest in autonomy as per self-determination theory | Provide youth with time to rest, reflect, think. Facilitate trusting dialogue between youth and significant others about their futures. |
| Goemans, M., van Breda, A. D., & Kessi, S. (2021). Experiences of young people preparing to transition out of cluster foster care in South Africa. *Child and Adolescent Social Work Journal, 38*(2), 227-237. <https://doi.org/10.1007/s10560-020-00704-1> | Self-determination | Self-determination (Ryan & Deci) helps shape a care-leaver’s space and life course. They decide to take up opportunities offered by the care setting. Self-determination is fuelled by competence, autonomy and relatedness | 2 | Study did not set out to investigate this theory, but findings were made sense of through extensive theory use | Self-determination is not autonomous, but relational – it emerges in the context a safe emotional relationship. |
| Hyde, R., & Atkinson, C. (2019). Care leavers’ priorities and the corporate parent role: A self-determination theory perspective. *Educational & Child Psychology, 36*(1), 40-57. <https://doi.org/10.53841/bpsecp.2019.36.1.40> | Self-determination | Self-determination theory’s (Ryan & Deci) three themes – competence, relatedness and autonomy – emerged in the findings regarding care-leavers’ experiences of pathway planning (preparing for adulthood). Given this, interdependence is a more meaningful goal and framing of the role of corporate parenting. | 1 | Study informed by theory | An interdependence model of transitional planning is proposed that considers the tensions between relatedness and autonomy in self-determination theory. |
| Itzhaki-Braun, Y., & Sulimani-Aidan, Y. (2022). Determination of life satisfaction among young women care leavers from the Ultraorthodox Jewish community. *Children and Youth Services Review, 136*, 106428. <https://doi.org/10.1016/j.childyouth.2022.106428> | Self-determination | Self-determination theory (Ryan & Deci) comprises competence, relatedness and autonomy. When these three needs are satisfied, well-being and optimal functioning can be attained. Religiosity, optimism and social support are linked to these. These ideas were tested with ultraorthodox women aging out of care. | 1 | Strong theory basis for the study | Competence, relatedness and autonomy are important needs of ultraorthodox female care-leavers. They need to learn to recognise and express their needs. Optimism needs to be strengthened. A supportive person is important, given the patriarchal and conservative culture of this group. |
| Jackson, M. S., Colvin, A. D., & Bullock, A. N. (2019). Strategies to address mental health challenges of foster youth transitioning to college. *Best Practices in Mental Health, 15*(1), 20-31. <https://doi.org/10.1007/s10560-022-00905-w> | Self-determination | Self-determination theory (Ryan & Deci) was evaluated for its utility in understanding the behaviour of care-leavers in higher education and their mental health. care-leavers in higher education experience greater mental health challenges than others in higher education. care-leavers are reluctant to seek help, due to stigma and mistrust. Yet self-determination theory says there is an innate drive to overcome adversity. | 2 | Self-determination theory informs but does not drive the study | Mentorship, academic support and counselling are needed to mobilise self-determination theory constructs: autonomy, competence, and relatedness. |
| Leathers, S. J., Holtschneider, C., Ludington, M., Ross, E. V., & Barnett, J. L. (2023). Mentoring, employment assistance, and enhanced staff outreach for older youth in care: Outcomes from a randomized controlled trial. *Children and Youth Services Review, 153*, 107095. <https://doi.org/10.1016/j.childyouth.2023.107095> | Self-determination | The importance of factors such as youth self-determination (Ryan & Deci) and investment in their goals, experiential opportunities to use new skills in a supported context, relationships with mentors or staff, and the impact of trauma experiences. The positive effects of using the positive youth approach are consistent with self-determination theory and its emphasis on building optimal social contexts to support motivation. | 2 | Theory used to interpret study findings | No theory-informed practice recommendations provided. |
| Olsson, T. M., Blakeslee, J., Bergström, M., & Skoog, T. (2020). Exploring fit for the cultural adaptation of a self-determination model for youth transitioning from out-of-home care: A comparison of a sample of Swedish youth with two samples of American youth in out-of-home care. *Children and Youth Services Review, 119*, 105484. <https://doi.org/10.1016/j.childyouth.2020.105484> | Self-determination | The study was interested in self-determination (Ryan & Deci). The purpose of this study was to explore the fit of a self-determination model (i.e., My Life) for youth transitioning from out-of-home care for the Swedish context by comparing baseline characteristics of two American samples of adolescents in out-of-home care who have participated in and benefited from experimental tests of the self-determination model with a sample of adolescents in care in Sweden. Studies show that self-determination enhancement interventions improve outcomes for youth transitioning from out-of-home care. | 1 | The study was based on this model as an intervention strategy. | The model calls for the cultural adaptation of imported interventions is a process to be approached systematically. Self-determination of youth should be promoted in programmes. |
| Turner, R., Vira, E. G., Bergström, M., & Olsson, T. M. (2023). Cultural adaptation of interventions and the a priori assessment of intervention fit: Exploring measurement invariance for American and Swedish youth leaving care. *Journal of Evidence-Based Social Work, 20*(3), 306-322. <https://doi.org/10.1080/26408066.2022.2159602> | Self-determination | The study explores the use of measurement invariance to help identify whether key theoretical constructs in a social work intervention’s theory of change fit differently in different contexts (US and Sweden). Self-determination (not cited) was the main theory informing the intervention’s theory of change. | 1 | Study design, findings and discussion was based on the theory. | Measurement invariance between US and Swedish adolescents was found. No theory-informed practice recommendations provided. |
| Olsson, T. M., Blakeslee, J., Bergström, M., & Skoog, T. (2020). Exploring fit for the cultural adaptation of a self-determination model for youth transitioning from out-of-home care: A comparison of a sample of Swedish youth with two samples of American youth in out-of-home care. *Children and Youth Services Review, 119*, 105484. <https://doi.org/10.1016/j.childyouth.2020.105484> | Self-efficacy | Self-efficacy (Bandura; Betz et al.) was seen as related to self-determination – some overlap of the constructs (e.g., enactive mastery and vicarious learning). | 1 | Study design included self-efficacy in three scales. | No theory-informed practice recommendations provided. |
| Forenza, B., & Lardier, D. T. (2017). Sense of community through supportive housing among foster care alumni. *Child Welfare, 95*(2), 91-115. <https://www.jstor.org/stable/48623569> | Sense of community | The sense of community (McMillan & Chavis), a sense of belonging to one’s residential community, is thought to improve care-leaving outcomes. Those living in supported accommodation experience strong sense and positive of community in their neighbourhood. | 1 | Study driven by theory | Care-leavers placed in support housing should be helped to form connections with their community. |
| Appleton, P. (2020). Anchors for deliberation and shared deliberation: Understanding planning in young adults transitioning from out-of-home care. *Qualitative Social Work, 19*(5-6), 1130-1146. <https://doi.org/10.1177/1473325019869810> | Shared deliberations | Shared deliberations (Bratman) focus “primarily on shared deliberations the young person may have with friends, family and the informal social network, and how this indicates what and who is personally important to the young person.” Deliberating with friends, family and informal social networks can improve decision making, informed by shared deliberations theory. | 1 | This is a theoretical paper | This approach counters the survivalist self-reliance that is thought to be harmful in the long run. |
| Berejena Mhongera, P., & Lombard, A. (2017). Who is there for me? Evaluating the social support received by adolescent girls transitioning from institutional care in Zimbabwe. *Practice, 29*(1), 19-35. <https://doi.org/10.1080/09503153.2016.1185515> | Social capital | Social capital (within Sustainable livelihoods) (Department for International Development) is used to answer the question, “what is the nature of the social support provided by stakeholders (individuals or organisations) during and after institutional care and how adequate is this support in meeting one’s livelihood needs?” (p. 21) Social capital is one of the 5 assets in Sustainable livelihoods, and “refers to social resources that individuals draw from to achieve their livelihood goals such as networks, membership of groups, relationships of trust and access to wider institutions of society.” The study identified sources of social capital, the social support they provided and the adequacy of that support. Support was not continued after leaving care, with the result that social capital did not help to secure livelihoods. | 1 | Study sets out to examine the contribution of social capital to care-leavers | A case management approach to ensuring a continuum of support to care-leavers through care into postcare is needed. |
| Cox, N., Martikke, S., Cumbers, H., & Webb, L. (2023). Reinventing selves and connections through community volunteering: How care leavers and their supporters create a space for agency and self-work. *Voluntary Sector Review, 14*(2), 293–313. <https://doi.org/10.1332/204080522X16454630207045> | Social capital | Social capital (Putnam) (with its focus on bonding, bridging and linking capital) is expanded in this study with two other related concepts: agency and self-work (Rose). The study was interested in how these theoretical concepts are developed through volunteering activity, and how they contribute to self-efficacy and an evolving self. | 1 | This cluster of theories inform the study throughout | A compassionate, pastoral, and relational approach may be helpful in working with care-leavers. |
| Gilligan, R., & Arnau-Sabatés, L. (2017). The role of carers in supporting the progress of care leavers in the world of work. *Child & Family Social Work, 22*(2), 792-800. <https://doi.org/10.1111/cfs.12297> | Social capital | Caregivers share social capital (not cited) with looked-after children in their care, by opening up new work opportunities, which can contribute to work/employment support. They ‘bridge’ young people into work using their contacts. | 1 | Study driven by the theory | Encourage caregivers to use their contacts as social capital to leverage work opportunities for young people. |
| Gimeno-Monterde, C., Gómez-Quintero, J. D., & C. Aguerri, J. (2021). Unaccompanied young people and transition to adulthood: Challenges for child care services. *Children and Youth Services Review, 121*, 105858. <https://doi.org/10.1016/j.childyouth.2020.105858> | Social capital | Social capital (Eriksson) is mentioned as an important personal asset of support, that is hard for people of different ethnic backgrounds to generate. The theory is mentioned in only one paragraph. | 3 | There is only passing reference to theory | No theory-informed practice recommendations provided. |
| Martikke, S., Cumbers, H., Cox, N., Webb, L., Gedzielewski, E., & Duale, M. (2019). Building bridges into the community: Social capital in a volunteering project for care leavers. *Children & Society, 33*(2), 111-125. <https://doi.org/10.1111/chso.12300> | Social capital | Aims to explore the roles of bridging and bonding social capital (Putnam) for care-leavers. Found that social capital was important, that bridging and bonding roles were often carried by the same person. Bonding capital forms a foundation for other social capital. Bonding forms through face-to-face relationships, which can later be maintained through social media. | 1 | Study driven by theory | Both bonding and bridging capital are important, with the former being a precondition for the capacity of the latter to be helpful. Partnerships across different services may help to create a network of different types of social capital. Face-to-face capital seems an important foundation. |
| Mendes, P. (2018). Towards the social inclusion of young people transitioning from out-of-home care: An examination of the Home Stretch campaign. *Social Alternatives, 37*(1), 59-62. <https://openjournals.library.sydney.edu.au/SWPS/article/view/12920/12020> | Social capital | Outcomes are influenced by “the availability of positive relationships via what is called social capital through professional and informal support networks” (p. 60). Theory not cited. | 3 | Mentioned in passing | No theory-informed practice recommendations provided. |
| Mullan, P. (2022). Care leaver’s outcomes in Ireland: The role of social capital. *Journal of Children’s Services, 17*(2), 97-110. <https://doi.org/10.1108/JCS-05-2021-0023> | Social capital | This paper aims to explore the outcomes experienced by young people leaving care in Ireland today through the theoretical lens of Bourdieu’s and Coleman’s social capital. In drawing upon the lived experience of these care-leavers, this work will discuss how their in-care and post-care experiences shaped their exposure to and development of sources of social capital, which in turn proved to be a significant factor in shaping their in-care and post-care outcomes. | 1 | Entire study is driven by this theory | EPIC (Empowering People in Care) should support the development of a national peer-to-peer support and mentoring network for children in care and care-leavers. Aftercare should be made available, on a statutory basis, to all young people leaving care. |
| Radey, M., Schelbe, L., McWey, L. M., & Holtrop, K. (2017). Me, myself and I: perceptions of social capital for mothers ageing out of the child welfare system. *Child & Family Social Work, 22*(2), 981-991. <https://doi.org/10.1111/cfs.12318> | Social capital | The focus of this study was mother and service provider perceptions of social relationships and support among mothers ageing out of the child welfare system. Using the social capital framework (Bourdieu), findings highlight social capital elements, social capital outcomes and capitalization present in the experiences of mothers ageing out. Results indicated that mothers lacked social capital, which was associated with needs in all aspects of support, including expressive and instrumental outcomes. | 1 | The entire study was shaped around the theory of social capital | Through Evidenced-based mentoring programmes mentors can increase mothers’ capitalization by assisting with basic tasks. A reliable mentor could be a resource for mothers ageing out as they learn life skills to help prevent limited collective assets from faltering. Regarding policy, findings support the need to extend the opportunity for youth to receive services to beyond age 18. (p. 989) |
| Shotton, L. H. (2021). From care to caring: Using Bourdieu to explore care experienced students journeys into and through nurse education. *Research in Post-Compulsory Education, 26*(4), 442-460. <https://doi.org/10.1080/13596748.2021.1980662> | Social capital | Bourdieu’s theory of practice and of social capital provides insight into understanding more about the journeys of five care-experienced students into, through and beyond nursing education. Although the study itself claims to use ‘theory of practice’, the term ‘practice’ is seldom used (9 times), while capital is used frequently (41 times). | 1 | Entire study was based on Bourdieu’s social capital | No theory-informed practice recommendations provided. |
| Simpson, D., & Murphy, S. F. (2022). ‘So we beat on, boats against the current, borne back ceaselessly into the past’: Legacy, care leavers and university study. *Journal of Youth Studies, 25*(2), 259-274. <https://doi.org/10.1080/13676261.2020.1865527> | Social capital | This study blended linked lives from life course theory (Brady & Gilligan) and bonding and bridging social capital (Webster et al.). The aim of the study was to capture information about care-leavers’ perceptions and experiences of higher education to add to understanding and insight in these areas. Many participants expressed this through wanting to prove others wrong – that they could achieve a higher education. | 1 | Theory was used in the thematic analysis, findings and discussion section of the study | No theory-informed practice recommendations provided. |
| Tobolowsky, B. F., Madden, E. E., & Scannapieco, M. (2017). Living on the edge: The postsecondary journeys of foster care alumni. *College Student Affairs Journal, 35*(1), 86. <https://doi.org/10.1353/csj.2017.0007> | Social capital | This study explores the postsecondary educational experiences of foster alumni and examines how their social networks or relationships support or impede their academic progress. Social capital theory (Bourdieu, Coleman & Putnum) provides a theoretical lens to explore the challenges as they enter higher education and the role networks, including student affairs professionals and others, may or may not play in their academic progression. | 3 | Used in only the literature review section of the article. And one sentence in conclusion. | Authors recommend peer mentors, who understand their circumstances and can help care-leavers resolve issues, training for personnel, and voluntary organisations or campus programmes to be put in place to manage and support alumni. |
| Tyrell, F. A., & Yates, T. M. (2017). A growth curve analysis of housing quality among transition-aged foster youth. *Child & Youth Care Forum, 46*(1), 91-117. <https://doi.org/10.1007/s10566-016-9370-1> | Social capital | The current study explored the level and change in housing quality among 172 youth across their first 2 years following their exit from the child welfare system. “Social capital theory holds that individuals with more material resources, knowledge, and information obtained via social relationships and networks, are more likely to experience positive developmental outcomes than their peers with less social capital (Laser & Leibowitz)” (p. 111). Exposure to domestic violence reduced social capital. Social relationships are necessary, but not sufficient for social capital. | 2 | Only addressed social capital in the findings section. | No theory-informed practice recommendations provided. |
| Van Breda, A. D. (2015). Journey towards independent living: A grounded theory investigation of leaving the care of Girls & Boys Town South Africa. *Journal of Youth Studies, 18*(3), 322-337. <https://doi.org/10.1080/13676261.2014.963534> | Social capital | One of the social processes identified in the study is networking for goal attainment. Social capital theory was used in the analysis of this process. | 2 | Mentioned in the findings and linked to one of the social processes | Author recommends care-leavers be taught skills to be aware of their social environment and distinguish strong from weak ties. |
| Van Breda, A. D. (2020). Patterns of criminal activity among residential care-leavers in South Africa. *Children and Youth Services Review, 109*, 104706. <https://doi.org/10.1016/j.childyouth.2019.104706> | Social capital | This paper reports on a longitudinal study of young people transitioning out of care and journeying towards young adulthood over a period of five years. Theoretical explanations for care-leavers transition into crime include the social capital theory (Cusick et al.). The author states that the lack of bonding among care-leavers, for example, may leave them vulnerable to criminal activity | 3 | Mentioned once only in literature section of article. | Author recommends “preventive intervention to ‘crime-proof’ young people preparing to leave care and an option to extend care beyond age 18, who do engage in crime – even fairly minor criminal activity – should be referred into a diversion programme” (p. 9) |
| Webb, L., Cox, N., Cumbers, H., Martikke, S., Gedzielewski, E., & Duale, M. (2017). Personal resilience and identity capital among young people leaving care: enhancing identity formation and life chances through involvement in volunteering and social action. *Journal of Youth Studies, 20*(7), 889-903. <https://doi.org/10.1080/13676261.2016.1273519> | Social capital | This study explores personal resilience, in the form of self-esteem (confidence), ego strength (integrity and sense of purpose) and self-determination (agency) is believed to also be dependent on individualisation opportunities and identity capital among care-leavers and young people in care engaging in social activities through volunteering. Findings suggest that social capital (Côté), which may be a factor in enabling their access after leaving care. | 2 | Links are made to the theory of social capital in the analysis section. | Authors recommend volunteering or similar activities, with ongoing support, may enhance social capital through group work, communication and interpersonal skills, with a resulting increase in personal capital such as confidence and self-belief. These “supported activities facilitate exposure to agentic development opportunities” (p. 900). |
| Butler, K., & Benoit, C. (2015). Citizenship practices among youth who have experienced government care. *Canadian Journal of Sociology (Online), 40*(1), 25-49. <https://doi.org/10.2307/canajsocicahican.40.1.25> | Social citizenship | Social citizenship (Marshall) for young people in care sees the state as a parent (unlike others who see the parent as the parent), thus being a citizen means being cared for a child of the state – “the state as parent” (p. 26). Looked-after young people tend to experience citizenship benefits less than others. Study found three different patterns by which young people express citizenship. | 1 | Study is driven by theoretical questions | No theory-informed practice recommendations provided. |
| Swan, M., Holt, S., & Kirwan, G. (2018). ‘Who do I turn to if something really bad happens?’ Key working and relationship-based practice in residential child care. *Journal of Social Work Practice, 32*(4), 447-461. <https://doi.org/10.1080/02650533.2018.1503161> | Social constructionism | The research methodology conducted in this study was “influenced by a phenomenological approach in conjunction with social constructionist theory, this frames the research approach for this study, as it is investigating young people’s experiences of key working in the social context of residential care” (p. 452). | 3 | Only used in methodology section | No theory-informed practice recommendations provided. |
| Park, K., & Courtney, M. E. (2023). Mitigating risks of incarceration among transition-age foster youth: considering domains of social bonds. *Child & Adolescent Social Work Journal, 40*(4), 473-486. <https://doi.org/10.1007/s10560-022-00891-z> | Social control | This study explores how different domains of social bonds affect legal system involvement for youth approaching adulthood from foster care. Social control theory (Hirschi) offered a framework for understanding crime in relation to social bonds. It suggests that crime engagement stems from broken social bonds, and thus youth with strong social bonds experience a reduced risk for engaging in crime—the more extensive youths’ social bonds are, the less youth are likely to engage in crime. Overall, the theory, suggesting that the likelihood of avoiding crime is influenced by relational bonds and connections to conventional institutions. | 1 | Theory is used as a conceptual base and to interpret findings | Institutional sources of social control, such as education and employment, can effectively reduce incarceration among youth transitioning from foster care to adulthood. |
| Lee, J. S., & Ballew, K. M. (2018). Independent living services, adjudication status, and the social exclusion of foster youth aging out of care in the United States. *Journal of Youth Studies, 21*(7), 940-957. <https://doi.org/10.1080/13676261.2018.1435854> | Social exclusion | A social exclusion framework (Lee & Patton) used “to examine associations between independent living service receipt, adjudication status, and early adult outcomes” among care-leavers (p. 940). Social exclusion was operationalised as homelessness and incarceration. Study found that “postsecondary education support and education financial services at baseline were related to a higher likelihood of being socially connected and lower likelihood of being socially excluded at age 19. Being an adjudicated delinquent and receiving special education and career services were related to a lower likelihood of being socially connected.” | 1 | Study driven by theory | No theory-informed practice recommendations provided. |
| Lee, J. S., Gimm, G., Mohindroo, M., & Lever, L. (2023). Assessing homelessness and incarceration among youth aging out of foster care, by type of disability. *Child & Adolescent Social Work Journal, 40*(6), 743-759. <https://doi.org/10.1007/s10560-022-00817-9> | Social exclusion | The study investigates individual factors and experiences that are associated with homelessness and incarceration to inform how environments and systems may be improved to better prevent social exclusion among youth aging out of care, especially those with a disability. Social exclusion framework (Lee & Patton) is an extension of the social model of disability in that the framework theorizes a process, which allows for an examination of the developmental transition to adulthood. Social exclusion is a process characterized by “catastrophic detachment” from conventional society, which occurs in multiple dimensions, such as the economic, spatial, and political. | 1 | Theory is used as a conceptual base and to interpret findings | Transitional services for the most high-risk youth should be bolstered. further extending transition services to age 25, especially for the most high-risk youth, may make a significant difference in reducing the likelihood of the social exclusion of these youth |
| Gwenzi, G. D., & Ringson, J. (2023). “Why would they call me an orphan when I have parents”: Care leavers’ experiences of labelling and stereotyping whilst living in residential care facilities in Zimbabwe. *New Ideas in Psychology, 68*, 100968. <https://doi.org/10.1016/j.newideapsych.2022.100968> | Social identity | Social identity (Tajfel) refers to the portion of one’s personal self-concept that is rooted in group membership. Conflict is explained as a function of group-based self-definitions. Stronger groups may pejoratively label weaker groups. Because most children in care (in Zimbabwe) were orphans, all children in care were termed ‘orphans’, including those who weren’t, leading to distorted social identities for non-orphan looked-after children. | 1 | The study set out to use a theory to explain a social phenomenon | Efforts should be made to decentre the term ‘orphan’ and to either use a more inclusive term or more accurate individual terms. Negative stereotyping should be challenged. |
| Bond, S. (2018). Care-leaving in South Africa: an international and social justice perspective. *Journal of International & Comparative Social Policy, 34*(1), 76-90. <https://doi.org/10.1080/21699763.2017.1413994> | Social justice | Social justice (Abel & Austin, Powers & Faden) is used to understand and critique social policy and legislation in four countries. Aspects of social justice theory are used to show how states fail care-leavers by not adequately drafting and implementing social policy to care for them. | 1 | Study sets out to use a social justice lens throughout | Implicit recommendation that states development and implement social policy for care-leavers as an expression of social justice. |
| Kind, N., Seker, S., d’Huart, D., Bürgin, D., Jenkel, N., Boonmann, C., Habersaat, S., Urben, S., Fegert, J. M., Clemens, V., Bürgin, D., & Schmid, M. (2023). High-risk substance use and psychosocial functioning in young adult care leavers: Findings from a 10-year follow-up study. *Children and Youth Services Review, 155*, 107290. <https://doi.org/10.1016/j.childyouth.2023.107290> | Social learning | This longitudinal study aimed to examine the relationship between persistence in high-risk substance use and psychosocial functioning in young adults who were previously placed within child welfare and juvenile justice institutions in Switzerland. Social learning theory (Bandura) is mentioned that suggests that adaptive and maladaptive behaviour are learned by observing others | 3 | Mentioned twice in the report-background to study and at the end of discussion. | Developing and testing peer-interventions and conducting research targeted towards the social context of substance use within residential care are important from a social-learning theoretical perspective, as peer context can potentiate but also protect from aversive substance use. |
| Mertz, M., & Andersen, S. H. (2017). The hidden cost of foster-care: new evidence on the inter-generational transmission of foster-care experiences. *British Journal of Social Work, 47*(5), 1377-1393. <https://doi.org/10.1093/bjsw/bcw132> | Social learning | Children learn by observing and emulating their parents’ positive and negative behaviours, which can facilitate intergenerational transmission of foster care experiences (Dunlap et al.). This emphasises an intergenerational route via parenting experiences. Even when controlling for parental resources, parental foster care experience still predicts child’s foster care experience, perhaps resulting from social learning. | 1 | Study driven by theory | No theory-informed practice recommendations provided. |
| Lee, J. S., Gimm, G., Mohindroo, M., & Lever, L. (2023). Assessing homelessness and incarceration among youth aging out of foster care, by type of disability. *Child & Adolescent Social Work Journal, 40*(6), 743-759. <https://doi.org/10.1007/s10560-022-00817-9> | Social model of disability | The study investigates individual factors and experiences that are associated with homelessness and incarceration to inform how environments and systems may be improved to better prevent social exclusion among youth aging out of care, especially those with a disability. This paper’s conceptual framework has two parts, one which is Oliver’s social model of disability, which asserts that what causes individuals’ disability is not impairments themselves but “the disabling barriers faced in society” (p. 1024). The initial goal of the social model of disability was to create more accessibility for people with disabilities in societies and workplaces (Davis). | 2 | Recommendations implicitly rely on theory | Transition services should be bolstered and extension of transition services to age 25 will help to remove barriers to successful transitioning. |
| Blakeslee, J. E. (2015). Measuring the support networks of transition-age foster youth: Preliminary validation of a social network assessment for research and practice. *Children and Youth Services Review, 52*, 123-134. <https://doi.org/10.1016/j.childyouth.2015.03.014> | Social network | The paper draws on social network theory (Blaeslee). It “presents a methodological approach to measure foster youth support networks, and describes these networks in terms of their capacity to provide support as a function of size, composition, and density, and in terms of actual support provision through identified relationships” (p. 123). The study considers “network characteristics as an explanatory factor in foster youth transition outcomes” (p. 124) using network theory. The findings indicate that the personal network measurement protocol predicts continued educational enrolment. | 1 | Developed a method for analysing care-leavers’ social networks and findings resulting from it | Recommend further research on social networks and use of the tool they have developed. Implicitly recommend continued emphasis on developing the social networks of young people preparing to leave care. |
| Marion, É., Paulsen, V., & Goyette, M. (2017). Relationships matter: Understanding the role and impact of social networks at the edge of transition to adulthood from care. *Child & Adolescent Social Work Journal, 34*(6), 573-582. <https://doi.org/10.1007/s10560-017-0494-4> | Social network | The social networks (Forsé) of care-leavers were studied. Most, but not all, care-leavers were socially connected, but had smaller networks that other youth. Two thirds regarded their care worker as an important part of their network. Quality of relationships not associated with care-leaving outcomes. | 1 | Study driven by theory | Relationship between care-leaver and youth worker is critically important. |
| Sundly, A., Keating, M., Effiong, A., & Saif, A. O. (2022). Neuroscientific evidence and care leaving: a multidisciplinary critical commentary. *International Journal of Child, Youth & Family Studies, 14*(1), 30-46. <https://doi.org/10.18357/ijcyfs141202321283> | Social reproduction | The study explores why legislators and policymakers use evidence from neuroscience and economics to justify increased investment in early childhood, but not in emerging adults. Using Bourdieu’s theory of social reproduction, preference is given to the type of knowledge that preserves the social structures that work to ensure the multigenerational flow of capital among dominant groups. | 1 | Entire study is based on social reproduction theory | Based on the social reproduction theory, recommendations are made that while investment in early childhood development is important, based on neurodevelopment at that age, investment in emerging adults is similarly important, based on neurodevelopment at that age. Policies should be continuously critically reviewed and challenged. |
| Atwool, N. (2020). Transition from care: Are we continuing to set care leavers up to fail in New Zealand? *Children and Youth Services Review, 113*, 104995. <https://doi.org/10.1016/j.childyouth.2020.104995> | Social support | Various forms of informal social support (Greeson et al.) are necessary, including from family, peers and mentors. These are explained. | 1 | This is a theoretical or conceptual paper | A wide range of social supports are necessary for improved care-leaving. |
| Curry, S. R., & Abrams, L. S. (2015). Housing and social support for youth aging out of foster care: State of the research literature and directions for future inquiry. *Child & Adolescent Social Work Journal, 32*(2), 143-153. <https://doi.org/10.1007/s10560-014-0346-4> | Social support | A combination of formal and informal support is necessary in the care-leaving journey. Social support (Cohen & Syme) has multiple functions, e.g. instrumental and affective. As much as self-sufficiency (ito housing) is an important goal, it needs to be within the context of supportive social connections. Policy tends to push for self-sufficiency and self-reliance as enablers of secure housing. | 2 | Theory is used, but does not drive the article | Policies should prioritise both independent living and supportive connections as jointly important for care-leaving outcomes. |
| Melkman, E. P. (2017). Childhood adversity, social support networks and well-being among youth aging out of care: An exploratory study of mediation. *Child Abuse & Neglect, 72*, 85-97. <https://doi.org/10.1016/j.chiabu.2017.07.020> | Social support | Social support networks (Uchino) are found to mediate the relationship between earlier adversity and current well-being. In addition, higher levels of family dysfunction were associated with smaller, less contact and less satisfaction with support networks among care-leavers, confirming Vranceanu et al.’s theory of the mediating role of social support. | 1 | Study guided by theory | Targeted interventions are required to increase social support and its availability. The social skills of youth should also be developed to reciprocally facilitate a supportive relationship. |
| Melkman, E. P., & Benbenishty, R. (2018). Social support networks of care leavers: Mediating between childhood adversity and adult functioning. *Children and Youth Services Review, 86*, 176-187. <https://doi.org/10.1016/j.childyouth.2018.01.040> | Social support | Social support networks can mediate adversity (Turner; Vranceanu). The “relationship [between childhood adversity and adult functioning] was fully or partially mediated by social support, across the various types of support and outcomes examined. Network size and network adequacy were the most prominent predictors of functioning; the latter more consistently so. Whereas emotional, practical, and information and guidance support all significantly contributed to greater adjustment to post-school settings and economic wellbeing, only practical support was related to fewer housing difficulties” (p. 176). | 1 | Study guided by theory | Care-leavers need instrumental support to navigate out of care. Ties with family should be promoted. Care-leavers social skills should be developed. |
| Gwenzi, G. D. (2023b). Provision of transitional housing: A socially sustainable solution for care leavers in Zimbabwe. *Practice, 35*(2), 103-120. <https://doi.org/10.1080/09503153.2022.2083100> | Social sustainability | Social sustainability (Cuthill) addresses the often-neglected social dimensions of sustainability, with a focus on social justice and equity, social infrastructure, engaged governance and social capital. This study aimed to explore the usefulness of social sustainability for the provision of social services to care-leavers, specifically transitional housing. | 1 | The study was designed to explore the usefulness of social sustainability | Transitional housing should be provided to care-leavers, even in resource constrained settings, due to the sustainable benefits it holds for care-leaver outcomes. |
| Dima, G., & Bucuta, M. D. (2015). The process of transition from public care to independent living: A resilience-based approach. *Revista de Cercetare si Interventie Sociala, 50*, 53-65. <http://www.rcis.ro/images/documente/rcis50_04.pdf> | Stein’s groups | Draws on Stein’s three groups of care-leavers: moving on, surviving and strugglers. | 3 | Stein’s groups only mentioned | No theory-informed practice recommendations provided. |
| Häggman-Laitila, A., Salokekkilä, P., Satka, M., Toivonen, K., Kekolahti, P., & Ryynänen, O.-P. (2019). The coping of young Finnish adults after out-of-home care and aftercare services: A document-based analysis. *Children and Youth Services Review, 102*, 150-157. <https://doi.org/10.1016/j.childyouth.2019.05.009> | Stein’s Groups | Stein has categorised care-leavers into three groups: moving on, surviving and strugglers. Used data to divide a group of care-leavers into these three groups. Survivors were the largest group (75%), followed by the moving on group (19%) and strugglers (7%). | 1 | The theory drove the analysis of data | Need to provide increased support with planning for leaving care. The extension of care to age 25 will allow a gradual transition for the strugglers. |
| Keshri, A. K. (2023). Understanding orphaned young people’s experience of leaving care in India: A qualitative study. *Developmental Child Welfare, 5*(2), 79–92. <https://doi.org/10.1177/25161032231171973> | Stein’s groups | This study explored the Indian orphaned young people’s perspectives on leaving care. Conducting interviews with 13 Indian youth with significant care experience in institutional and residential arrangements. The understanding of care-leaving presented in the study was based on retrospective insights that young people with care experience shared regarding their experiences of leaving care. Findings categorise care-leavers as fitting into Stein’s ‘extended and abrupt’ group. | 2 | Stein’s theory briefly in the literature review section and discussion | Transition planning should start early and extended support after leaving care are recommended. |
| Neagu, M., & Sebba, J. (2019). Who do they think they are: Making sense of self in residential care, foster care, and adoption. *Children and Youth Services Review, 105*, 104449. <https://doi.org/10.1016/j.childyouth.2019.104449> | Stigma | The author states that stigma can lead to self-identifying as bad, dangerous or weak. Findings that when care-leavers disclose their care-leaving status to friends their trust, the relationship comes closer, to confirm Goffman’s stigma theory. “Young people that were in placements where they were listened to, were supported to overcome stigma and to re-establish their self-esteem, managed to achieve identity”. “This finding supports Goffman’s claim that when people become closer, stereotyping is replaced by sympathy, understanding and a realistic assessment of the person.” (p. 3) | 1 | Study was driven by theory and analysis looked at aspects of the theory | Authors findings suggest that stigmatization is reported at all levels of care, therefore children in care should be provided extra support from carers and professionals in overcoming this challenge. Teachers should be trained on how to support children in care to manage their care status while at school. |
| Collins, M. E., & Augsberger, A. (2021). Impacts of policy changes on Care-Leaving Workers in a time of coronavirus: Comparative analysis of discretion and constraints. *Journal of Comparative Policy Analysis: Research and Practice, 23*(1), 51-62. <https://doi.org/10.1080/13876988.2020.1841560> | Street-level bureaucracy | Street-level bureaucracy (Lipsky; Smith & Donaldson) themes include discretion and accountability, professionalism, and resources and constraints. The theory suggests the workers could use their discretion to make their work easier, or to go out of their way to advocate for a client. During COVID-19, extra guidelines were provided to workers, to prioritise those most vulnerable. | 1 | The study design and analysis were shaped by the theory | The extra leeway to exercise discretion among child welfare workers during COVID-19 may fall away post COVID. |
| McCormick, M., Anthony, J., & Townsend, E. T. (2023). Fostering safer spaces: Retaining and empowering SGM students with a lived history of foster care. *Child & Adolescent Social Work Journal, 40*(2), 255-270. <https://doi.org/10.1007/s10560-022-00889-7> | Strength-based | Strength-focus (Saleebey) is an important part of any social work act and speaks to aspects of the NASW Code of Ethics, which encourage social work­ers to see the value of human relationships and to respect the inherent dignity and worth of those with whom they work. As such, the authors added a more specific strengths focus to the second round of litera­ture review and included a focus on resiliency. | 2 | Mentioned as a theoretical framework and only briefly later | Helping care-leavers recognise the power they acquired in overcoming previous rejections focuses on their strengths, to empower them, to believe they can make it to college. |
| Nho, C. R., Park, E. H., & McCarthy, M. L. (2017). Case studies of successful transition from out-of-home placement to young adulthood in Korea. *Children and Youth Services Review, 79*, 315-324. <https://doi.org/10.1016/j.childyouth.2017.06.035> | Strength-based | The study adopted a strength-based approach (Harwick et al.) to the study, to identify how care-leavers managed to successfully transition out of care. | 1 | Theory driven. Links made to findings | No theory-informed practice recommendations provided. |
| Radey, M., Schelbe, L., McWey, L. M., Holtrop, K., & Canto, A. I. (2016). “It’s really overwhelming”: Parent and service provider perspectives of parents aging out of foster care. *Children and Youth Services Review, 67*, 1-10. <https://doi.org/10.1016/j.childyouth.2016.05.013> | Strength-based | Not used as a theory, but a term. Strengths theory not cited. The goal of this research was to understand the daily experiences of parents aging out, their strengths, and their needs from parents’ and providers’ perspectives. | 3 | Mentions the word “strength” in the literature and findings but no link to the strengths perspective. | Authors recommend a structured peer and mentoring support for parents. |
| Samarah, E. M. S., Schelbe, L., & Jackson, L. A. (2023). A photovoice study of college students who have experienced foster care, relative care, and/or homelessness. *Children and Youth Services Review, 151*, 107042. <https://doi.org/10.1016/j.childyouth.2023.107042> | Strength-based | This study documents strengths-based approaches (not cited) to supporting youth with foster care and homelessness experiences by describing experiences being a college student, and becoming “Unconquered” - in other words resilient. Related to resilience, being “unconquered” can be understood as a dynamic process of strength building in the face of adversity. | 3 | Mentioned three times in article | No theory-informed practice recommendations provided. |
| Sulimani-Aidan, Y. (2018d). Promoting resilience among adolescents in care from their social workers’ perspectives. *Children and Youth Services Review, 94*, 43-48. <https://doi.org/10.1016/j.childyouth.2018.09.005> | Strength-based | Social workers use this strength-based perspective (Saleebey) in their intervention programs to enhance young people’s resilience. The study investigates has to how they lay the foundation for this perspective, | 3 | Not much mentioned on the perspective. | Author recommends that the implications for practice mean working on more intervention programmes from a strength-based perspective to further promote resilience |
| Berejena Mhongera, P. (2017). Preparing for successful transitions beyond institutional care in Zimbabwe: adolescent girls’ perspectives and programme needs. *Child Care in Practice, 23*(4), 372-388. <https://doi.org/10.1080/13575279.2016.1215291> | Sustainable livelihoods | Sustainable livelihoods (Department for International Development) are used “to assess people’s capabilities, assets and the livelihood strategies they pursue to achieve their livelihood goals”. Sustainable livelihoods are used to interpret how participants construct a ‘successful transition’ out of care. All care-leavers included livelihood assets (human, social, physical, financial and natural) in their construction. | 1 | Study set out to understand what Sustainable livelihood assets are required | Care-leaving programmes should promote acquisition of assets that facilitate livelihoods. Positive youth development programmes and mentoring are suggested. |
| Berejena Mhongera, P., & Lombard, A. (2016). Poverty to more poverty: An evaluation of transition services provided to adolescent girls from two institutions in Zimbabwe. *Children and Youth Services Review, 64*, 145-154. <https://doi.org/10.1016/j.childyouth.2016.03.013> | Sustainable livelihoods | Sustainable livelihoods (Department for International Development) are used to evaluate the services provided to adolescent girls transitioning out of care. To what extent did these services develop the sustainable livelihood assets? They found that the programmes did not address the Sustainable livelihood assets, with the result that care-leavers experienced poverty postcare. | 1 | Set out the understand how care-leaving programmes facilitate sustainable livelihoods | Programmes (before and after leaving care) need to work to develop the sustainable livelihood assets to reduce poverty experienced after leaving care. |
| Berejena Mhongera, P., & Lombard, A. (2018). Promoting successful transitions beyond institutional care: A programme-based service delivery model linked to a case management system. *Social Work/Maatskaplike Werk, 54*(1), 53-68. <https://doi.org/10.15270/54-1-614> | Sustainable livelihoods | Sustainable livelihoods (Department for International Development) not used to inform the study. The study itself is empirically driven, comparing a programme against contracted standards. A programme-based case management system is proposed, but is not clear how this is informed by sustainable livelihoods. | 2 | Sustainable livelihoods mentioned throughout the study, but is not a key focus of the study. Rather the focus is on a programme. | Authors recommend implementing their system, which is informed by sustainable livelihoods principles. |
| Macleod, G., Dallas-Childs, R., Brough, C., & Toye, M. (2021). ‘She just got me’: Supporting care experienced young people negotiating relationships and identities at school. *Journal of Research in Special Educational Needs, 21*(S1), 25-35. <https://doi.org/10.1111/1471-3802.12543> | Symbolic interactionism | The study examines the complex relationship between care experience, mental health and exclusion from school and how young people negotiate these different experiences and identities. The study took a symbolic interactionist lens (Meltzer & Petra) to the analysis and showed how the interaction with significant others shapes young people’s identities. | 1 | The entire study is based using this theory | Improved collaboration between all professionals working in children’s services will also be of benefit. For all participants, managing their care identity in interactions with peers, caused them the most difficulty. This suggests a need to educate all pupils on understandings of the wider systemic reasons why some children are placed in care, and a need for schools to look out for, and respond to, bullying. |
| Thompson, R. W., Duppong Hurley, K., Trout, A. L., Huefner, J. C., & Daly, D. L. (2017). Closing the research to practice gap in therapeutic residential care: Service provider–university partnerships focused on evidence-based practice. *Journal of Emotional and Behavioral Disorders, 25*(1), 46-56. <https://doi.org/10.1177/1063426616686757> | Teaching-family | The study offers an approach to address evidence-supported practices into service provider programs. This was based on this family-teaching model (Thompson & Daly) that originated in Boys Town and has appeared to have very positive outcomes in working with young juveniles. | 3 | Limited reference to the theory surrounding the model. | Authors recommend partnership and collaborative work between universities and residential treatment centres. |
| Leathers, S. J., Holtschneider, C., Ludington, M., Ross, E. V., & Barnett, J. L. (2023). Mentoring, employment assistance, and enhanced staff outreach for older youth in care: Outcomes from a randomized controlled trial. *Children and Youth Services Review, 153*, 107095. <https://doi.org/10.1016/j.childyouth.2023.107095> | Theory of change | This study contributes to examining the impact of job readiness training, mentoring, and outreach. Youth were interviewed at baseline when they were 17–20 years old and 14 months later to assess youth outcomes including employment, school enrolment, delinquency, and depression programmes. The intervention’s theory of change posited that youth would benefit from the intervention through an increase in adult support, which would support positive outcomes across. The implicit model of change in many programs is that skill deficits pose a major barrier to retaining housing and employment as well as completion of educational programs. | 3 | Mentioned once in the literature review article | These outcomes were unrelated to changes in social support needs, counter to expectations. Future research is needed to build on these findings and those from other programs with positive effects in other areas such as educational outcomes to create effective service systems for older youth in care and development of an integrated service model with more consistent positive effects on outcomes in early adulthood. The development of a coordinated network of services to better support youth. |
| Neagu, M., & Sebba, J. (2019). Who do they think they are: Making sense of self in residential care, foster care, and adoption. *Children and Youth Services Review, 105*, 104449. <https://doi.org/10.1016/j.childyouth.2019.104449> | Threatened identity | Several interviewees reported that disclosure supported them in making friends among peers, something they had avoided before in order to escape the threatening position of stigma. Sharing their care status with a few peers, in a strategic way overtime is reflected in several of the narratives in the study, confirming Breakwell’s claim that self-disclosure to a small number of people can be part of isolation, a coping strategy for threatened identities. Threatened identity theory (Breakwell) assisted in understanding care-leavers journey of self and describes some aspects to put in place to assist young people leaving care | 1 | Used in the analysis of the data collected. | No theory-informed practice recommendations provided. |
| Storø, J. (2017). Which transition concept is useful for describing the process of young people leaving state care? A reflection on research and language. *European Journal of Social Work 20*(5), 770-781. <https://doi.org/10.1080/13691457.2016.1255879> | Transition | The concept of transition was looked at in terms of a process. There is a claim that the field lacks theory, and that several theoretical perspectives are needed to understand the transition of young people. 18 papers were studied which are seen as contributions to a pre-theorisation stage. However, they do not provide a clear answer to the question of whether there is a need for a comprehensive transition theory. | 1 | Transition as a concept is used throughput the article to explain the care-leaving process. | Due to the difficulty of understanding of the concept of transition, of both males and females, on the journey from care to adulthood, the author recommends that the concept further be developed in future research. |
| Söderqvist, Å., Bülow, P. H., & Sjöblom, Y. (2015). “In Sweden work is more important than the culture, actually!” The care leaving process for unaccompanied youths from the perspective of social workers. *Transnational Social Review, 5*(3), 241-257. <https://doi.org/10.1080/21931674.2015.1082778> | Transnational | The focus of this article is to present reflections about the care-leaving process for unaccompanied youths from a professional social work perspective. More specifically, it is to explore and clarify the meaning making that the professionals do during care-leaving in relation to the unaccompanied youths’ minority backgrounds. This theoretical framework relies on aspects outlined by the transnational perspective to look at the variety of possible ways to interpret and understand the processes of leaving care. The transnational perspective added another dimension, making it possible to focus on specific circumstances surrounding this group of care-leavers. | 1 | The whole study is based on the transnational perspective. | No theory-informed practice recommendations provided. |
| Fusco, R. A., & Kulkarni, S. J. (2018). “Bedtime is when bad stuff happens”: Sleep problems in foster care alumni. *Children and Youth Services Review, 95*, 42-48. <https://doi.org/10.1016/j.childyouth.2018.10.024> | Trauma | A ‘trauma theory framework’ (no author cited) is indicated, but the text is in this regard is empirical. Study finds relationship between experiences of trauma (before and in care) to sleep disturbance, and further disturbances after care. | 3 | Trauma theory is not discussed, only trauma as a phenomenon | No theory-informed practice recommendations provided. |
| Mølholt, A. K., Bengtsson, T. T., & Frederiksen, M. (2023). Navigating educational success: Modes of expectation among care‐experienced young people. *British Educational Research Journal*, Advance online publication. <https://doi.org/10.1002/berj.3908> | Trust, risk and uncertainty | Explores modes of expectation among care-experienced young people when navigating educational success. Based on Luhmann’s theory of trust, risk and uncertainty, the study conceptualizes three distinct ‘modes of expectation’ that each represent a specific way of experiencing uncertainty and developing expectations about future outcomes of decisions and actions depending on the level of complexity and the young person’s ability to reduce the resulting uncertainty. | 1 | Theory is used as a conceptual base and to interpret findings | No theory-informed practice recommendations provided. |
| Butterworth, S., Mendes, P., & Flynn, C. (2020). Young people leaving out-of-home care in Victoria, Australia: An exploration of factors influencing positive transitions. *Social Work & Policy Studies, 3*(2), 1-23. <https://openjournals.library.sydney.edu.au/SWPS/article/view/14772/13401> | Turning points | The paper does not have a strong theoretical focus, but is interested in “factors influencing positive transitions” of care-leavers, most phrased as ‘turning points’ (Hutchison), which aligns with resilience theory and a strengths-based approach. Various findings concerning what facilitated transitions from care are presented. | 2 | The study was broadly but not rigorously informed by theory | Supportive networks are important to facilitate leaving care. |
| Goemans, M., van Breda, A. D., & Kessi, S. (2021). Experiences of young people preparing to transition out of cluster foster care in South Africa. *Child and Adolescent Social Work Journal, 38*(2), 227-237. <https://doi.org/10.1007/s10560-020-00704-1> | Ubuntu | Ubuntu theory (Van Breda) argues that a person is only a person through their connections with other persons. For those in care, ubuntu is experienced in the foster care setting (with foster ‘siblings’ and ‘parents’). Ubuntu is comparable to the western notion of interdependence. | 2 | Study did not set out to investigate this theory, but findings were made sense of through theory use | Ubuntu requires relationships of community connectedness and mutual care and support. |
| Moodley, R., Raniga, T., & Sewpaul, V. (2020). Youth transitioning out of residential care in South Africa: Toward ubuntu and interdependent living. *Emerging Adulthood, 8*(1), 45-53. <https://doi.org/10.1177/2167696818812603> | Ubuntu | Ubuntu (Van Breda) is an African worldview that speaks, among others, to the interconnectedness or interdependence of all people and that no person stands alone or separate from others. Ubuntu is often clan-based, so that children are raised within the clan, even if not by their parents, and share a surname with the clan. Independence is not a goal, but rather interdependence. Westernisation and neoliberalism have undermined Ubuntu. South African policy tends to be western rather than African in its ambitions. Study findings support the underlying importance of Ubuntu values. | 1 | Theory driven study | The push for independence should be replaced with interdependent living. Multiple systems should be mobilised to provide supportive contexts for care-leavers. |
| Paul, J. C. (2020). Exploring support for LGBTQ youth transitioning from foster care to emerging adulthood. *Children and Youth Services Review, 119*, 105481. <https://doi.org/10.1016/j.childyouth.2020.105481> | Youth mentoring | The primary objectives for the study included identifying who provides support for LGBTQ youth, the types of support provided, and whether the youth had any unmet needs related to supports and services. Youth mentoring theory (Rhodes) explains how youths’ connections to supportive non-parental adults in the child welfare system (e.g. caseworkers, foster parents, group home staff) can influence young adult outcomes. More specifically, this theory suggests that establishing meaningful relationships with these adults can lead to improved socio-emotional, cognitive and identity development among foster youth, and ultimately more positive outcomes in adulthood. | 1 | Study is based on this framework | No theory-informed practice recommendations provided. |
