# Supplementary material for: Theories used to explain care-leavers’ journey out of care: A scoping review
Source: PLoS One. 2025 Jun 13;20(6):e0325776. doi: 10.1371/journal.pone.0325776 (PMC12165428; doi:10.1371/journal.pone.0325776)
Supplement: Attachment — Submitted filename: PLOS one review Dec 2024.docx [file pone.0325776.s003.docx]

PLOS ONE

Theories used to explain care-leavers’ journey out of care: A scoping review

--Manuscript Draft--

| Manuscript Number: | PONE-D-24-53785 |
| --- | --- |
| Article Type: | Research Article |
| Full Title: | Theories used to explain care-leavers’ journey out of care: A scoping review |
| Short Title: | Theories used to explain care-leavers’ journey out of care: A scoping review |
| Corresponding Author: | Adrian D. van Breda, D Lit et Phil University of Johannesburg SOUTH AFRICA |
| Keywords: | leaving care; theory; scoping review; resilience; emerging adulthood; attachment; ecological |
| Abstract: | This study was motivated by Mike Stein’s 2006 critique of care-leaving research as reflecting a ‘poverty of theory’. A scoping review of care-leaving journal articles was conducted for the nine-year period from 2015 to 2023. 252 articles met the inclusion criteria, including that theory must be explicitly mentioned. 133 theories were used across these publications, with resilience theory being used in 24% of articles, life course in 10%, emerging adulthood and attachment in 8%, social capital in 6% and ecological theory in 4%. Over half of the publications were driven by theory (from conceptualisation, through research design, to interpretation of findings), while a fifth were informed in parts by theory and a quarter alluded only briefly to theory. Three quarters of the articles utilised theory to formulate practice recommendations for care- leaving services. Only two theories could be identified that were constructed to explain care-leaving. Although most theories considered the care-leaver within their social environment, there was little use of structural, systemic, critical and rights-oriented theories. The study concludes that Stein’s original concern has been somewhat addressed over the past several years. Other studies find that around a quarter to half of care-leaving publications use theory. This study confirms that, of those studies that make at least some use of theory, most weave theory firmly into the study and mobilise that theory to make recommendations for practice. Nevertheless, research should become yet more theory-driven, contextual, systemic, rights-oriented and critical theories should be used more frequently, and more needs to be done to build theory for care-leaving. |
| Order of Authors: | Adrian D. van Breda, D Lit et Phil |
|  | Sasambal Reuben, PhD |
| Additional Information: |  |
| Question | Response |
| Financial Disclosure  Enter a financial disclosure statement that describes the sources of funding for the work included in this submission. Review the [submission guidelines](http://journals.plos.org/plosone/s/submission-guidelines#loc-financial-disclosure-statement) for detailed requirements. View published research articles from [*PLOS ONE*](https://journals.plos.org/plosone/) for specific examples.  This statement is required for submission and will appear in the published article if the submission is accepted. Please make sure it is accurate. | The author(s) received no specific funding for this work. |

*Powered by Editorial Manager® and ProduXion Manager® from Aries Systems Corporation*

| Funded studies  Enter a statement with the following details:   - Initials of the authors who received each award - Grant numbers awarded to each author - The full name of each funder - URL of each funder website - Did the sponsors or funders play any role in the study design, data collection and analysis, decision to publish, or preparation of the manuscript? |  |
| --- | --- |
| Did you receive funding for this work? |  |
| Competing Interests  Use the instructions below to enter a competing interest statement for this submission. On behalf of all authors, disclose any [competing interests](http://journals.plos.org/plosone/s/competing-interests) that could be perceived to bias this work—acknowledging all financial support and any other relevant financial or non- financial competing interests.  This statement is required for submission and will appear in the published article if the submission is accepted. Please make sure it is accurate and that any funding sources listed in your Funding Information later in the submission form are also declared in your Financial Disclosure statement.  View published research articles from  [*PLOS ONE*](http://journals.plos.org/plosone/) for specific examples. | The authors have declared that no competing interests exist. |

*Powered by Editorial Manager® and ProduXion Manager® from Aries Systems Corporation*

| NO authors have competing interests  Enter: *The authors have declared that no competing interests exist.*  Authors with competing interests  Enter competing interest details beginning with this statement:  *I have read the journal's policy and the authors of this manuscript have the following competing interests: [insert competing interests here]* |  |
| --- | --- |
| * typeset |  |
| Ethics Statement  Enter an ethics statement for this submission. This statement is required if the study involved:   - Human participants - Human specimens or tissue - Vertebrate animals or cephalopods - Vertebrate embryos or tissues - Field research   Write "N/A" if the submission does not require an ethics statement.  General guidance is provided below. Consult the [submission guidelines](http://journals.plos.org/plosone/s/submission-guidelines#loc-guidelines-for-specific-study-types) for detailed instructions. Make sure that all information entered here is included in the Methods section of the manuscript. | N/A |

*Powered by Editorial Manager® and ProduXion Manager® from Aries Systems Corporation*

| Format for specific study types  Human Subject Research (involving human participants and/or tissue)   - Give the name of the institutional review board or ethics committee that approved the study - Include the approval number and/or a statement indicating approval of this research - Indicate the form of consent obtained (written/oral) or the reason that consent was not obtained (e.g. the data were analyzed anonymously)   Animal Research (involving vertebrate animals, embryos or tissues)   - Provide the name of the Institutional Animal Care and Use Committee (IACUC) or other relevant ethics board that reviewed the study protocol, and indicate whether they approved this research or granted a formal waiver of ethical approval - Include an approval number if one was obtained - If the study involved *non-human primates*, add *additional details* about animal welfare and steps taken to ameliorate suffering - If anesthesia, euthanasia, or any kind of animal sacrifice is part of the study, include briefly which substances and/or methods were applied   Field Research  Include the following details if this study involves the collection of plant, animal, or other materials from a natural setting:   - Field permit number - Name of the institution or relevant body that granted permission |  |
| --- | --- |
|  |  |
| Data Availability  Authors are required to make all data underlying the findings described fully available, without restriction, and from the time of publication. PLOS allows rare exceptions to address legal and ethical concerns. See the [PLOS Data Policy](http://journals.plos.org/plosone/s/data-availability) and [FAQ](http://journals.plos.org/plosone/s/data-availability#loc-faqs-for-data-policy) for detailed information. | Yes - all data are fully available without restriction |

*Powered by Editorial Manager® and ProduXion Manager® from Aries Systems Corporation*

| A Data Availability Statement describing where the data can be found is required at submission. Your answers to this question constitute the Data Availability Statement and will be published in the article, if accepted. |  |
| --- | --- |
| Important: Stating ‘data available on request from the author’ is not sufficient. If your data are only available upon request, select ‘No’ for the first question and explain your exceptional situation in the text box. |  |
| Do the authors confirm that all data underlying the findings described in their manuscript are fully available without restriction? |  |
| Describe where the data may be found in full sentences. If you are copying our sample text, replace any instances of XXX with the appropriate details.   - If the data are held or will be held in a public repository, include URLs, accession numbers or DOIs. If this information will only be available after acceptance, indicate this by ticking the box below. For example: *All XXX files are available from the XXX database (accession number(s) XXX, XXX.*). - If the data are all contained within the manuscript and/or Supporting Information files, enter the following: *All relevant data are within the manuscript and its Supporting Information files.* - If neither of these applies but you are able to provide details of access elsewhere, with or without limitations, please do so. For example:   *Data cannot be shared publicly because of [XXX]. Data are available from the XXX Institutional Data Access / Ethics Committee (contact via XXX) for researchers who meet the criteria for access to confidential data.*  *The data underlying the results presented in the study are available from (include the name of the third party* | All relevant data are within the paper and its Supporting Information  files. As this is a scoping review of other research, the paper contains methodology and reporting based on the ‘Preferred Reporting Items  for Systematic Reviews and Meta-Analysis’. The paper includes database and search strategies and findings, which support replication of the article. There is no ethical or legal restrictions relating to this paper and the submission contains all raw data required to replicate the results of the study. |

*Powered by Editorial Manager® and ProduXion Manager® from Aries Systems Corporation*

| *and contact information or URL).*   - This text is appropriate if the data are owned by a third party and authors do not have permission to share the data.   * typeset |  |
| --- | --- |
| Additional data availability information: |  |

*Powered by Editorial Manager® and ProduXion Manager® from Aries Systems Corporation*

Article manuscript	[Click here to access/download;Manuscript;Care-leaving](https://www2.cloud.editorialmanager.com/pone/download.aspx?id=38373575&guid=a3c5ffe9-18d3-4752-95c6-b04e7bb26d6e&scheme=1)[<https://www2.cloud.editorialmanager.com/pone/download.aspx?id=38373575&guid=a3c5ffe9-18d3-4752-95c6-b04e7bb26d6e&scheme=1> theories v8 - paper.docx](https://www2.cloud.editorialmanager.com/pone/download.aspx?id=38373575&guid=a3c5ffe9-18d3-4752-95c6-b04e7bb26d6e&scheme=1)

1

1 ***Theories used to explain care-leavers’ journey out of care:***
2 ***A scoping review***
3 Adrian D. van Breda^1¶^*, Sasambal Reuben^2&^
4 [avanbreda@uj.ac.za,](mailto:avanbreda@uj.ac.za) <https://orcid.org/0000-0002-9984-9180>
5 [sasamn@hotmail.com,](mailto:sasamn@hotmail.com) <https://orcid.org/0009-0003-6316-1127>
6 ^1^ Department of Social Work & Community Development, University of Johannesburg,
7 Johannesburg, South Africa
8 ^2^ Department of Social Work & Community Development, University of Johannesburg,
9 Johannesburg, South Africa 10
11 * Corresponding author
12 Email: [avanbreda@uj.ac.za](mailto:avanbreda@uj.ac.za) (AvB) 13
14 ^¶^ Conceptualisation, Data curation, Conceptualised the study, Supervision, Formal analysis, Writing
15 – Original Draft Preparation
16 ^&^ Data curation, Formal analysis, Writing – Review & Editing

2

# 17 Abstract

18 This study was motivated by Mike Stein’s 2006 critique of care-leaving research as reflecting a
19 ‘poverty of theory’. A scoping review of care-leaving journal articles was conducted for the nine-
20 year period from 2015 to 2023. 252 articles met the inclusion criteria, including that theory must be
21 explicitly mentioned. 133 theories were used across these publications, with resilience theory being
22 used in 24% of articles, life course in 10%, emerging adulthood and attachment in 8%, social
23 capital in 6% and ecological theory in 4%. Over half of the publications were driven by theory
24 (from conceptualisation, through research design, to interpretation of findings), while a fifth were
25 informed in parts by theory and a quarter alluded only briefly to theory. Three quarters of the
26 articles utilised theory to formulate practice recommendations for care-leaving services. Only two
27 theories could be identified that were constructed to explain care-leaving. Although most theories
28 considered the care-leaver within their social environment, there was little use of structural,
29 systemic, critical and rights-oriented theories. The study concludes that Stein’s original concern has
30 been somewhat addressed over the past several years. Other studies find that around a quarter to
31 half of care-leaving publications use theory. This study confirms that, of those studies that make at
32 least some use of theory, most weave theory firmly into the study and mobilise that theory to make
33 recommendations for practice. Nevertheless, research should become yet more theory-driven,
34 contextual, systemic, rights-oriented and critical theories should be used more frequently, and more
35 needs to be done to build theory for care-leaving.

36

# 37 Keywords

38 leaving care, theory, scoping review, resilience, emerging adulthood, attachment, ecological

# 39 Introduction

40 Children and adolescents, whose families are unable to provide them with adequate care, come to the
41 attention of child welfare services and, in some cases, are placed in alternative care [1, 2], also known
42 as out-of-home care [3] or foster care [4]. Alternative care may range from a short-term emergency
43 placement to a permanent placement and may include foster care (with a relative or non-related foster
44 carer), residential or group home care (including children’s homes that may be small family-like
45 homes or larger institutional settings) or adoption. The principle preference is for children to remain
46 in their family of origin (with their parents or other family members) or another family (non-related
47 foster care), rather than in residential placements [1].
48 While in principle, placements out of the family should be as brief as possible [1], in practice,
49 alternative care placements often continue for two or more years, often until the young person reaches
50 the legal age at which they must leave care (usually at age 18), though care can often be extended by
51 a few additional years [5, 6]. At this stage, the young person typically must transition out of the child
52 protection or child welfare system towards independent living. This transition is known as ‘leaving
53 care’ or ‘care-leaving’, and the young people leaving care as ‘care-leavers’ [7].
54 There has been a rapidly growing interest in care-leaving and care-leaver outcomes over the past
55 several years [8]. This is most prominent in North America, Australia and Europe, but also now in
56 many countries in Africa and Asia [8-13].
57 The interest in care-leaving results from several factors, particularly the body of research that shows
58 the comparatively negative outcomes of care-leavers compared with other young people transitioning
59 out of families [14-17]. While some have argued that it is alternative care itself that produces these
60 outcomes [18], others argue that the children who come into the alternative care system are already
61 at higher levels of vulnerability than children who do not [19, 20].
62 Notwithstanding the growing body of literature on the transition out of care and post-care outcomes
63 or markers, Stein, in his seminal 2006 paper [21], lamented the poverty of theory underpinning this
64 research, which had tended to be empirical and descriptive. He writes, “Although there is a growing
65 body of international empirical work on young people aging out of care, very few of these studies
66 have been informed by theoretical approaches” [21]. His paper proposes three theories that could be
67 useful in informing care-leaving research – attachment, focal and resilience theories – within an
68 overall framework of social exclusion.
69 Many authors have heeded Stein’s call; much of the current care-leaving literature appears to have a
70 stronger theoretical base and some work is being done to develop a theory of care-leaving. For example,
71 the first part of the edited book by Mann-Feder and Goyette [8] is termed ‘theoretical perspectives’,
72 and covers developmental [22], network [23], resilience [24] and human rights [25] theories. A recent
73 edited volume [10] foregrounds theories and/or theoretical constructs like stability and instability, the
74 interpersonal-psychological theory of suicide, social capital and resilience.
75 However, while there appears to be a growth in theory-informed and theory-building research, there
76 has not been a review of these theories, to determine what theories are being used and how they are
77 used to explain the care-leaving transition. Such a review would be helpful in mapping out the range
78 of theories that inform the journey out of care. This could, in turn, strengthen the development of
79 theory-informed interventions for care-leavers.
80 The aim of this paper, therefore, is to report the findings of a scoping literature review of recent care-
81 leaving literature to answer the following question: What theories are used or being generated to
82 explain the transition out of care towards young adulthood?
83 The following sub-questions will be asked:
84 1. What are the most frequently used theories to explain care-leaving?
85 2. To what extent does theory inform care-leaving research?
86 3. To what extent do theories inform practice recommendations?
87 4. What bespoke care-leaving theories have been constructed?
88 The following section will set out the methodology used in conducting this review. The reporting of
89 the review was informed by the Preferred Reporting Items for Systematic Review and Meta-Analysis
90 (PRISMA) Statement and checklist [26] (S1 Checklist). We did not register a scoping review protocol
91 for this study. Thereafter, the findings of each of the research questions will be set out. The findings
92 will finally be discussed, conclusions drawn about the state of theory in care-leaving research and
93 recommendations made for the way forward.

94

# 95 Methodology

96 MunnPeters [27] state that a scoping review is differentiated from a regular literature review in that
97 it “includes exhaustive search for information”, aims to be “transparent and reproducible” and
98 includes “multiple reviewers” to improve reliability. Scoping reviews act as a way for gaps in the
99 current literature to be recognised, allowing further research to be conducted. The authors conducted
100 a scoping literature review of studies that used one or more theories to explain care-leavers’ journey
101 out of care. To the authors’ knowledge, this is the first scoping literature review of the theories that
102 are used to explain care-leavers’ journey out of care.

103

## 104 Search strategy

105 A scoping review search typically includes a selection of information sources inclusive of electronic
106 databases. For the purposes of this study, a selection of electronic databases was searched. Searches
107 were conducted in February 2024. An initial meeting was held with an independent expert, who was
108 well versed in the field of literature searches and who guided and advised on the manner to proceed
109 with the initial search. Thereafter a comprehensive search of literature was undertaken within the
110 following databases: EBSCOhost, Project Muse, ProQuest Central, ProQuest Sociology, Sage,
111 Science Direct, Scopus, SpringerLink, Taylor & Francis and Wiley Online.
112 The following search terms were used for all databases:
113 "Leaving care" AND theor*
114 An initial search using a wider range of terms could not be accommodated by several of the databases
115 and was thus abandoned in favour of these simpler terms. In addition, an initial search of the full texts
116 of the publications in the databases generated tens of thousands of hits, with very few relevant ones;
117 thus, the search terms were restricted to title, keywords and abstract.

118

## 119 Inclusion and exclusion criteria

120 The inclusion and exclusion criteria were the following:
121 1) All studies had to have a publication date (including an early view or online date) within the nine-
122 year period of 2015 to 2023;
123 2) Publications had to be written in English, for practical reasons and to avoid the cost of translation,
124 but were not limited to any location, country or region;
125 3) We excluded books, book chapters, book reviews, conference proceedings, editorials, policy
126 guidelines and grey literature;
127 4) Journal articles had to focus on young people preparing to leave care, young people in the process
128 of leaving care or people who had left care (whether recently or long ago); and
129 5) The articles had to include (at least in passing) theory used or developed to explain care-leavers’
130 transition out of care.
131 The year 2015 was selected as the start date for the search because the authors opted to use Van
132 Breda’s South African grounded theory study [28] as a point of reference for this study. We regard
133 his paper as a first attempt in South Africa to develop a care-leaving theory, and thus pegged it as our
134 starting point for the review. To ensure trustworthiness of the data, the EBSCOhost database was
135 used as a pilot study, and we thereafter carried out the same search on the other databases. According
136 to Shamseer, Moher [29], piloting is seen as strategic in the selection process “to minimize errors”,
137 thereby allowing for further trustworthiness in the overall study.

138

## 139 Screening steps

140 The authors worked co-operatively throughout the search process, examining and reviewing each
141 other’s work. This was important to ensure trustworthiness of the study.
142 1) We conducted the pilot search of EBSCOhost together and deliberated over the search terms to
143 ensure they balanced inclusivity and manageability.
144 2) We then conducted the searches of all the remaining databases together, exporting all data to .ris
145 or .enw files. These were imported into EndNote, which we used as our data management
146 platform.
147 3) The first author used EndNote’s “Find Duplicates” function to identity and delete duplicate
148 articles. This author also manually scanned all the remaining articles to identify and delete
149 duplicates.
150 4) The remaining articles were divided in half, with each author screening their half of the articles
151 for the inclusion/exclusion criteria. This review was based primarily on the title and abstract, and
152 where necessary, the full text was downloaded to finalise the decision. When there was
153 uncertainty about excluding an article, we consulted with each other. We then screened all of each
154 other’s work in full to ensure consensus. A list of reasons for excluding articles was kept.
155 5) The first author downloaded and attached the full texts of each article into the database of screened
156 publications. These were again divided in half, with each author reading and screening half the
157 manuscripts. This led, in some cases, to additional articles being dropped due to not meeting the
158 theory criterion (for example, articles that mentioned using grounded theory methods but that
159 didn’t develop a theory of leaving care). The authors consulted each other on all such exclusions.
160 6) The first author ‘cleaned up’ the EndNote database, ensuring correct formatting of titles,
161 sequencing of author first and last names, ensuring DOI numbers, etc.
162 7) The final collection of papers included in the scoping review totalled 252 records.
163 The PRISMA flow diagram of the study selection process is displayed in Figure 1.
164 **Figure 1.** PRISMA flow diagram

165

## 166 Data analysis

167 The authors exported the references from EndNote into a Word table format and again divided the
168 final collection of publications between them. Each article was scrutinised to identity the theory or
169 theories that were used in the article. When an article mentioned more than one theory, the entry of
170 that article was duplicated, so that each theory per article could be analysed. We then analysed how
171 the theory was used to explain the care-leaving process and whether there were any practical
172 applications emerging from the theory (not from the findings per se), writing our notes into the data
173 extraction table (see S1 Table).
174 Through this process of analysing the data we began to recognise that some publications made
175 extensive use of theory while others only passing use. Extensive conversations in relation to the
176 articles led to the development of a typology of the role or place of theory in the publications. An
177 iterative process of formulating the typology and then testing it against articles led us to a three-fold
178 typology, viz. theory-driven studies, theory-informed studies and theory-alluded studies, which will
179 be discussed in the findings.
180 In our analysis, we worked both to quantify the theories used and how they are used, and to
181 qualitatively illustrate the use of theory in explaining care-leaving processes.

182

# 183 Findings

184 A total of 252 articles met the screening criteria and comprise the dataset for this study. Some
185 articles appear more than once in the dataset, because multiple theories in one article created
186 multiple rows in the table. In total, the review comprises 342 theory-article pairs. The distribution of
187 articles and theory-article pairs per year is depicted in Figure 2. The graph shows no trend in either
188 the number of theory-informed care-leaving publications per year over the nine-year period –
189 articles range from 14 to 42 - nor the number of theory-article pairs – which ranged from 17 to 56.
190 The average theories per article per year range from 1.21 to 1.56 across the nine years.
191 **Figure 2.** Theory-informed care-leaving publications per year 192

## 193 Theories used

194 All articles in this review used at least one theory, as this was core to the sampling criteria. On
195 average, each article used 1.36 theories. In total, 133 theories were identified. Ninety-four (70.7%)
196 of these theories were used in only one article and a further 17 (12.8%) theories were used in only
197 two articles – these will be listed, but not discussed. The remaining 22 (16.5%) theories were each
198 used in three to 64 articles and form the focus of analysis. S1 Table contains details of all articles
199 and theories, for the interested reader.
200 The 94 theories that were used only once are: Adultification [30], Anchors for deliberation [31],
201 Anti-Black Racism [30], Arrested adulthood [32], Behaviour Determinants Intervention [33], Care
202 [34], Citizenship [35], Communities of Practice (CoP), Conservation of resources [36],
203 Constellations of participation [37], Continuum [38], Convoy model [39], Critical social theory of
204 youth empowerment [40], Cultural capital [41], Cultural connectedness [42], Cumulative
205 advantages and disadvantages [43], Decoloniality [44], Delayed adulthood [32], Dialectical critical
206 realism [45], Drama therapy [46], Dramaturgical approach [47], Dyadic responses to trauma theory
207 [48], Early maladaptive schemas [49], Emotional intelligence [50], Emotional-management model
208 [51], Epistemic injustice [52], Existential well-being [53], Experience [54], Explanatory modelling
209 [55], Family practices approach [43], Family systems [56], Family theory [57], Feminism [58],
210 Feminist pathways [59], Future perspectives [60], General strains [61], Habitus [62], Home [63],
211 Hope [64], Human Capability Approach [65], Human capital [66], Human development [67],
212 Identity capital [68], Individualisation [69], Institutional ethnography [70], Institutionalised
213 vulnerability [71], Kinning [72], Learned helplessness [73], Lifespan theory of control [74],
214 Marxism [75], Mentoring [76], Minority stress [77], Multilevel model of college access [78],
215 Narrative-based model of identity [79], Neoliberalism [80], Network [81], Nordic welfare model
216 [82], Objectification [83], Occupational justice [84], Open systems [85], Person-centred [86],
217 Person-in-Environment (PIE) [87], Personal construct [88], Planning norms [31], Positioning [89],
218 Reciprocity [90], Relational autonomy [91], Resistance [34], Resources [92], Sanctuary Model [93],
219 Self as social construction [94], Self-efficacy [95], Sense of community [96], Shared deliberations
220 [31], Social citizenship [97], Social constructionism [98], Social control [99], Social identity [100],
221 Social justice [101], Social model of disability [102], Social reproduction [103], Social
222 sustainability [104], Stigma [105], Street-level bureaucracy [106], Symbolic interactionism [107],
223 Teaching-family [108], Theory of change [109], Threatened identity [105], Transition [110],
224 Transnational [111], Trauma [112], Trust, risk and uncertainty [113], Turning points [114], and
225 Youth mentoring [77].
226 The 17 theories that were used only twice are: Ambiguous loss [115, 116], Belonging [117, 118],
227 Critical disability [70, 119], Empowerment [84, 120], Ethic of care [121, 122], Family [123, 124],
228 Hierarchy of needs [125, 126], Homeostasis [127, 128], Institutional logics [119, 129], Motivation
229 [130, 131], Narrative therapy [46, 132], Precarity [133, 134], Psychosocial [135, 136], Social
230 exclusion [102, 137], Social learning [92, 138], Social network [139, 140], and Ubuntu [115, 141].
231 Table 1 lists the 22 theories that were used in three or more articles in descending order of
232 frequency. Percentages in the frequency column refer to the percentage of the 252 articles in which
233 each theory is used. Together, these 22 theories account for 63% of all the theory-article pairs (214
234 out of 342). The remaining columns (Driven, Informed and Alluded) will be discussed later in the
235 article.
236 **Table 1.** Prominent theories for care-leaving

| **Theory** | **Citations** | **Frequency** | **Driven** | **Informed** | **Alluded** |
| --- | --- | --- | --- | --- | --- |
| Agency | [107, 142-146] | 6 (2%) | 4 (67%) | 2 (33%) | 0 (0%) |
| Attachment | [28, 39, 48, 92, 147-161] | 19 (8%) | 8 (42%) | 4 (21%) | 7 (37%) |
| Developmental social  welfare | [146, 162, 163] | 3 (1%) | 0 (0%) | 0 (0%) | 3 (100%) |
| Ecological | [48, 146, 164-171] | 10 (4%) | 5 (50%) | 4 (40%) | 1 (10% |
| Emerging adulthood | [5, 32, 44, 63, 103, 157,  172-186] | 21 (8%) | 8 (38%) | 3 (14%) | 10 (48%) |
| Focal | [5, 187-189] | 4 (2%) | 1 (25%) | 1 (25%) | 2 (50%) |
| Future orientation | [60, 126, 178, 179] | 4 (2%) | 3 (75%) | 0 (0%) | 1 (25%) |
| Identity | [190-192] | 3 (1%) | 2 (67%) | 1 (33%) | 0 (0%) |
| Interdependence | [35, 190, 193] | 3 (1%) | 3 (100%) | 0 (0%) | 0 (0%) |
| Intersectionality | [69, 194, 195] | 3 (1%) | 2 (67%) | 0 (0%) | 1 (33%) |
| Journey towards  independent living | [28, 196, 197] | 3 (1%) | 3 (100%) | 0 (0%) | 0 (0%) |
| Life course | [43, 66, 68, 184, 198-217] | 24(10%) | 18 (75%) | 3 (13%) | 3 (13%) |

| **Theory** | **Citations** | **Frequency** | **Driven** | **Informed** | **Alluded** |
| --- | --- | --- | --- | --- | --- |
| Liminality | [133, 134, 198] | 3 (1%) | 2 (67%) | 0 (0%) | 1 (33%) |
| Possible selves | [173, 218, 219] | 3 (1%) | 2 (67%) | 1 (33%) | 0 (0%) |
| Recognition | [133, 134, 220] | 3 (1%) | 3 (100%) | 0 (0%) | 0 (0%) |
| Resilience | [28, 34, 36, 39, 65, 68, 71,  89, 95, 131, 147, 150, 152,  157, 160, 163, 170, 173,  178, 180, 189, 196, 197,  206, 212, 214, 221-258] | 64 (25%) | 29 (45%) | 16 (25%) | 19 (30%) |
| Self-determination | [95, 109, 115, 254, 259-262] | 8 (3%) | 5 (63%) | 3 (38%) | 0 (0%) |
| Social capital | [28, 61, 206, 246, 257, 263-  272] | 15 (6%) | 8 (53%) | 3 (20%) | 4 (27%) |
| Social support | [190, 273-275] | 4 (2%) | 3 (75%) | 1 (25%) | 0 (0%) |
| Stein’s groups | [226, 231, 276] | 3 (1%) | 1 (33%) | 1 (33%) | 1 (33%) |
| Strength-based | [195, 239, 244, 250, 277] | 5 (2%) | 1 (20%) | 1 (20%) | 3 (60%) |
| Sustainable  livelihoods | [58, 278, 279] | 3 (1%) | 2 (67%) | 1 (33%) | 0 (0%) |

237

238 It should be noted that theories are sometimes difficult to label or frame, because some theories and
239 theoretical constructs overlap and others form components of larger theories. For example, life
240 course theory features frequently, but comprises several sub-themes, such as linked lives. Some
241 articles [e.g., 216] centred on linked lives and not the other components of life course, while other
242 articles focus on life course, giving limited attention to linked lives [e.g., 43]. In such cases, we
243 endeavoured to indicate this by coding the theory at multiple levels, e.g., “Life course – linked
244 lives” in the case of Brady & Gilligan [216], and simply “Life course” in the case of Adeboye et al.
245 [43].
246 Resilience theory [280, 281] is the most prominent theory used to explain care-leaving. It appears in
247 a quarter of the articles. While resilience theory used to be highly individualistic and could be used
248 to infer that care-leavers were entirely responsible for their own care-leaving journey, a
249 socioecological approach to resilience is increasingly used [e.g., 237, 250, 282], attending to
250 personal, relational and systemic or structural resilience enablers. Resilience theory has the
251 advantage of helping to identify the kinds of resilience processes that facilitate a smoother transition
252 out of care.
253 Life course theory [283] is the second most prevalent theory in care-leaving research, appearing in a
254 tenth of articles. It helps to explain how one’s journey through life is influenced by personal and
255 environmental factors, and thus connects with cognate concepts like agency, linked lives and social
256 capital. It was seldom used in the past – only eight articles in the five years from 2015 to 2019 – but
257 has become prominent this decade – 15 articles in the three years from 2020 to 2023 [e.g., 204,
258 284].
259 Emerging adulthood [285] is the third most prevalent theory (8%). It refers to a stage of human
260 development corresponding roughly to ages 18-29, which is the age range in which most care-
261 leaving takes place and is thus the age range of most care-leaving research. Although emerging
262 adulthood theory has several core concepts (as does life course theory), much of the care-leaving
263 research that uses emerging adulthood uses the term primarily to refer to an age range [e.g., 183,
264 286, 287].
265 Attachment theory [288] is a fourth theory shaping the theorisation of care-leaving (8%). This is
266 understandable given that being placed in care frequently results from ruptures in children’s
267 primary attachment relationships and the removal from the family and placement into care, while
268 the later aging out of care are additional disruptions of attachment. Notions of relational
269 permanence, belonging and placement stability are informed by attachment theory, and are
270 considered key to the well-being of care-leavers [289]. Attachment theory is thus important for
271 care-leaving research [e.g., 150, 152, 155].
272 Social capital, with its concepts of bonding, bridging and linking [290], is the fifth most prominent
273 theory used in recent care-leaving publications (6%). Social capital intersects with resilience and
274 life course theories, making it a rich connector for theorisation. All three categories of social capital
275 are shown to be helpful for care-leavers in different ways and thus an important construct for this
276 population [e.g., 257, 267, 268].
277 The last theory to be presented here was found in 10 articles (4%): ecological or social ecological
278 theory [291]. This theory aligns with the person-in-environment principle that is core to social
279 work’s identity [292, 293]. It links to resilience and life course theories, and also to attachment and
280 social capital, because of its focus on the social environment. It thus informs several care-leaving
281 studies [e.g., 170, 171, 294].
282 It is not possible in this article to review all 133 theories that appear in care-leaving publications
283 over the past nine years. It is apparent, though, that there is a wide spectrum of theories that could
284 be helpful for theorising young people’s experiences of transitioning out of care into young
285 adulthood, and that some of those that are not in the six we have briefly discussed here may be
286 pregnant with possibilities, as seen in life course theory that has rapidly risen into prominence in the
287 past few years. Other theories that are marginal in care-leaving research, such as possible selves,
288 ethic of care, liminality, institutional logics and anti-Black racism, could be found by researchers to
289 be valuable to shed fresh light on the leaving care journey.

290

## 291 Theory-informed care-leaving publications

292 This study did not aim to determine what percentage of care-leaving publications incorporated
293 theory. Instead, it aimed to understand how theory was used in those papers that did include theory.
294 Nevertheless, in the process of gathering data, several topic-focused scoping reviews provided
295 information on the percentage of papers that included a theoretical framework. Gahagan et al.’s
296 [295] scoping review of publications on post-secondary education among care-leavers found that
297 only 13 out of 58 studies (22%) published between 1997 and 2022 stated an explicit theoretical
298 framework. Eight of these used social-ecology, three life course, and one each intersectionality,
299 feminist and phenomenological.
300 Another scoping review, conducted by Stubbs, Baidawi [296], focused on care-leavers’ experience
301 of informal support. Fifty-eight papers, published from 2013 to 2020, were included in the review,
302 thus a more recent sample of publications than Gahagan et al.’s study above. The authors reported
303 that 57% of the studies included theoretical explanations, and that resilience theory was the most
304 prevalent theory (one quarter of publications). Thereafter, 14% used social capital theory, 12%
305 emerging adult theory, and 9% attachment theory, comparable to the results of this study (see Table
306 1) even though their study had a narrower focus on informal support for care-leavers than our focus
307 on care-leavers per se.
308 Hodgson, Cordier [297] developed a matrix guide for analysing qualitative care-leaving data. As
309 part of their study, they mapped theories that inform care-leaving research into a graphic (Figure 3).
310 Larger circles imply more studies using those theories. The authors note the emphasis on individual
311 or personal theories, rather than macro, structural and sociological theories, and point to right-based
312 theories as emergent.
313 **Figure 3.** Theories of leaving care 314

## 315 Use of theories to explain care-leaving

316 Through the review of the use of theories of the selected articles, we identified three main ways in
317 which theories were used or the place of theory in the studies. We termed these as theory-driven,
318 theory-informed and theory-alluded studies. All three of these categories did use theory, but in
319 different ways. Over half of the papers were theory driven (53%), a fifth were theory informed
320 (21%) and a quarter were theory alluded (26%).
321 First, theory-driven studies were those in which theory was introduced prior to the methodology
322 section, thereby shaping the study design. Many of these studies constructed the research question
323 in theoretical terms, e.g., to apply a theory to the data or to test a theory’s relevance in practice. In
324 most of these cases, the theory was woven throughout the study, from introduction to conclusion.
325 We found that over half (n=197, 58%) of theory-article pairs were theory driven.
326 For example, Barratt, Appleton [142] build on a previous related study [145] to explore the
327 relevance of Archer’s concept of internal conversations within her broader theory of agency.
328 Archer’s theory is introduced and explicated in the opening pages of the study. The study aim is
329 framed within the theory, viz. “Our aim in this small-scale research project was to use the internal
330 conversations model to explore how care leavers come to understand, interpret and then act (or not
331 act) in relation to their current context and past experiences” [142]. The research methodology
332 explicitly focuses on these internal conversations. For example, the first interview with care-leavers
333 was an “in-depth, semi-structured, interview which focused on their internal conversations” and the
334 interview scheduled “was informed by Archer (2003, 161-162)” [142].
335 The eight themes that emerged from Barratt et al.’s [142] study are similarly framed within
336 Archer’s theory, for example, “The fourth theme, internal conversations as unhelpful or ineffectual,
337 highlights that for some care leavers internal conversations could be experienced as unhelpful or
338 even harmful”, and “The last three themes all relate to the overarching complexity of social
339 relationships for this group and how this was expressed and worked through in internal
340 conversation”. The discussion reflects on the findings and draw on Archer’s theory and other
341 studies on internal conversations. For example, “we found that the internal conversations and
342 meaning-making of the participants are powerfully shaped by their experiences of trauma and
343 disappointment, which may inhibit their sense of themselves as active agents in the world” [142].
344 The theory drivenness of this study is crudely evident in the fact that the term ‘internal
345 conversation’ appears 61 times in the article. A comparable example is the study of Bennwik and
346 Oterholm [35] on policy values related to support for care-leavers with disabilities. A key theory
347 informing this study is citizenship, even though the term does not appear in the title and only once
348 near the end of the abstract. However, the term is used 33 times in the article, appearing on every
349 page except the first page.
350 Bennwik and Oterholm [35] introduce the term ‘citizenship’ on the second page in a section titled
351 ‘central theoretical perspectives’, and on the following page they theorise the construct, stating,
352 “Citizenship as a concept has changed and developed over time, but fundamentally it deals with
353 questions of membership in society based upon certain rights and obligations.” They refer to
354 Nussbaum in asserting that “all human beings are citizens, independently of their contributions to
355 society”.
356 The study was conducted through a document analysis, which included a search for the term
357 ‘citizenship’. They extended the notion of citizenship to inclusive citizenship, that de-emphasised
358 the contribution a citizen makes to society. For care-leavers with disability, this meant decentring
359 the notion of ‘independent adulthood’ and centring on the notion of ‘inclusive citizenship’.
360 Second, theory-informed studies were those in which theory was either introduced early in the
361 paper, but not pulled through into the methodology, findings and discussion, or introduced mainly
362 in the discussion section, as a way of retrospectively illuminating and explaining the research
363 findings. The study may have been driven primarily by empirical concerns and not framed within a
364 theory. But once the data was in hand, the researchers identified one or more theories to help make
365 sense of and theoretically contextualise the study findings.
366 We found that a fifth (n=67, 20%) of articles were theory informed.
367 Some theory-informed studies introduce theoretical concepts in the opening pages of the article, but
368 do not pull them through to the methodology, findings and conclusions. For example, Goyette,
369 Blanchet [149] examined the “role of placement instability on employment and educational
370 outcomes among adolescents leaving care”. On the second page, in a literature review, they write an
371 intensive paragraph on attachment theory, which they argue helps to explain “the negative outcomes
372 associated with placement instability” [149], citing Bowlby and numerous other authors. After page
373 2, attachment is never mentioned again until the reference list. Although attachment theory
374 apparently informed the conceptualisation of the study, in relation to placement instability, it did not
375 drive the study in the way described in the previous two theory-driven studies.
376 Other theory-informed studies brought theory in towards the end to interpret findings. For example,
377 Mupaku, Van Breda [189] studied the experience of care-leavers with disabilities transitioning out
378 of residential care during the COVID-19 pandemic. The study is not theoretically framed up front.
379 But in the discussion of the findings, social-ecological resilience is introduced to explain how
380 COVID-19 led to an atrophy of social-ecological resources – family, friends, work, education –
381 which lowered the resilience of care-leavers and made them more vulnerable to deterioration in
382 behaviour and mental health.
383 The authors also draw on focal theory, which is used to explain how the transition out of care
384 during ordinary times entails a pile-up of transitions that overwhelm the capacity of many care-
385 leavers. For care-leavers with disabilities who are transitioning out of care during COVID-19, the
386 pile-up increased substantially, overloading care-leavers with too many transitions at one time.
387 Finally, in the recommendations, the authors recommend that in line with focal theory, transitional
388 tasks should be spread over a longer period, and in line with social ecological resilience theory,
389 care-leavers and their families need to be buffered with networks of social services.
390 Third, theory-alluded studies were those which mentioned theories or theoretical constructs in
391 passing, but did not link these clearly to the study or its findings. These studies were largely
392 designed to address empirical questions. They did not frame the study or the findings in theoretical
393 terms, but merely alluded to theory in passing.
394 We found that a quarter (n=78, 23%) of articles were theory alluded.
395 An article on patterns of criminal behaviour among care-leavers in South Africa [61] refers to two
396 theories in the literature review: general strains theory and social capital (or bonding capital) theory.
397 Each theoretical construct is explained and applied to criminal activity in a sentence or two. The
398 methodology is not informed by these theories, and they are not used in the discussion or
399 recommendations to explain the findings or make recommendations for policy or practice. These
400 theories are alluded to, but not utilised. Both could possibly have contributed to a richer analysis
401 and interpretation of the data.
402 An article by Dutta [165] explored the preparation of girls for leaving residential care in India. In
403 the discussion section, the author refers briefly to Bronfenbrenner’s ecological theory, identifying
404 three of the levels (micro, meso and macro). In a figure, six factors that affect preparation for
405 leaving care are plotted into these levels. Other than using the levels as headings, there is no further
406 discussion of the findings according to social ecological theory. Thus, this theory is alluded to in
407 this article, but not explicated or applied in any depth.

408

## 409 Practical application of theories

410 Most studies on leaving care produce recommendations or implications for practice. Among the 342
411 theory-article pairs in this review, 81 (24%) did not make recommendations informed by theory.
412 Some may have made recommendations based on the study findings but did not link these
413 recommendations to theory. On the other hand, three quarters of the theory-article pairs (76%) did
414 make recommendations that were, to at least some degree, informed by theory.
415 For example, Dumont, Lanctôt [218] published a possible selves theory-driven article on the hopes
416 and fears of girls transitioning out of care in Canada. In their implications for practice, they
417 recommended that social workers and other care workers should facilitate young people to reflect
418 on their hoped-for possible selves, and to develop these into viable future selves. On the other hand,
419 the girls needed support in avoiding their feared possible selves. They recommended also that they
420 explore the actual pathways to achieving these selves. And they encouraged workers to find role
421 models who aligned with each girl’s possible selves, to help strengthen and actualise these possible
422 selves into actual selves.
423 Berejena Mhongera [58] utilised sustainable livelihoods theory in her study of adolescent girls
424 transitioning from care in Zimbabwe. To build livelihoods in the face of the gender-specific
425 challenges girls face leaving care, she recommends including girls in participative decision making
426 at both personal and strategic levels to develop competencies required in adulthood. She
427 recommended fostering connections between societal systems to facilitate their participation in
428 these systems and to develop self-efficacy and the capacity to advocate. Access to a range of assets
429 should be facilitated, cognisant of the various ways girls are excluded from assets in a patriarchal
430 context, so that they can draw on these assets after leaving care.
431 Hollingworth and Jackson [187] reanalysed data from two previous studies in the UK and Europe
432 utilising focal theory, to determine if focal theory “would have helped predict their educational
433 progression or otherwise” [187]. They found that, indeed, care-leavers were often overwhelmed
434 with the pileup of challenges in rapid succession. The authors recommend that care-leavers be given
435 more latitude in planning their own pathways out of care, rather than being disengaged on their 18^th^
436 birthday. This would allow care-leavers to separate and sequence the numerous tasks and challenges
437 they must navigate in leaving care. The authors argue that practitioners need to understand focal
438 theory and its implications for transitional planning, which they suggest could be done in half a day.
439 Such training would make practitioners more mindful of the pileup of challenges and more
440 confident to find ways and take time to space these challenges out. Finally, they indicate that care-
441 leavers themselves would also benefit from an understanding of focal theory, so that they could
442 identify periods of pileup and negotiate more spacing for themselves.
443 Finally, we draw on six studies that use self-determination theory – four as theory-driven [95, 259-
444 261] and two as theory-informed studies [115, 262]. We are integrating the findings to illustrate
445 how multiple studies, drawing on a common theory, can be synergised to generate integrated
446 practice recommendations. These studies argue that self-determination is a relational construct,
447 rather than a personal one, and that both the capacity or opportunity to self-determine and processes
448 of self-determination occur in socio-cultural contexts. Females raised in ultraorthodox religious
449 communities, for example, may not be permitted to exercise self-determination, thus they need to
450 learn to recognise and express their needs in partnership with a supportive other. They also need to
451 be given life experiences that build optimism for greater self-determination. Thus, self-
452 determination – whether and how it is exercised – must be considered within each person’s cultural
453 contexts. All care-leavers need time to rest, reflect and think to enable the capacity for self-
454 determination. There is a need for opportunities to build trusting and emotionally safe relationships
455 with significant others, so that self-determination of their futures can be negotiated in relational
456 contexts. To this end, working towards interdependence, rather than independence as the goal for
457 leaving care is necessary. Mentoring is a potentially valuable practice for supporting self-
458 determination, by building voices of difference into a care-leavers’ network of relationships. In
459 combination, these interventions will foster relatedness, competence and autonomy, contributing to
460 effective self-determination.

461

## 462 Theories developed specifically for care-leaving

463 Only two studies could be found that appeared to construct a theory of or for care-leaving. Several
464 studies were sampled because the term ‘grounded theory’ was used, but excluded from the review
465 because there was no evidence of a ‘theory’ being formulated. There are various types of theories
466 [298], the most relevant of which for this paper was explanatory theories, i.e. theories that helped to
467 explain the care-leaving journey. Five studies that used grounded theory methods were included in
468 the review, but did not produce what we could recognise as ‘a theory of care-leaving’ [63, 183, 205,
469 299, 300].
470 A grounded theory study that did appear to meet the requirements of an explanatory theory was Van
471 Breda’s model of the ‘journey towards independent living’ [28, 197, 301], which comprises four
472 interconnected psychosocial processes: striving for authentic belonging, networking people for goal
473 attainment, contextual responsiveness and building hopeful and tenacious self-confidence. These
474 four processes are constrained or held by contextual boundaries and environmental interface [28].
475 After the original study, conducted only with male care-leavers, the study was replicated with
476 female participants. The original findings were confirmed [197] and two additional processes
477 specific to female care-leavers were identified, viz. embracing motherhood and taking on
478 responsibilities [301]. Ongoing research on this theory is currently underway, but publications fell
479 outside the timeframe of this study [e.g., 302].
480 Another study used Bayesian explanatory network models for building a theory or explanatory
481 model regarding “disruptive behaviour among Finnish care leavers who have been receiving
482 aftercare services for around two years” [55]. A model can be considered ‘explanatory’ if it presents
483 “the causal connection of the variables and interventions to the outcome variable” [55]. Disruptive
484 behaviour in this study includes regular substance use, threats of violence, problems managing
485 finances, and social interaction patterns [55]. Completing “secondary or vocational education and
486 primary placement at a foster family”, as well as the “use of employment office services … were
487 significantly associated with less disruptive behaviour” [55].

488

# 489 Limitations

490 Arguably, the biggest limitation of this review was the search term (“Leaving care” AND theor*).
491 Not all research on leaving care uses the term ‘leaving care’ or ‘care leaving’ – terms like ‘aging out
492 of care’ or ‘care-experienced people’ are used by many scholars. This may have resulted in relevant
493 publications being omitted from the search. We initially developed a comprehensive collection of
494 search terms, but found that most databases could not handle the multiple options or that the search
495 resulted in a massively overinclusive collection of mostly irrelevant information.
496 Furthermore, the words theory, theorise, theoretical, etc. were not necessarily stated in the article.
497 Publications may have referred to the resilience perspective or strengths approach, for example, and
498 would be missed because of the absence of the word ‘theor*’.
499 Notwithstanding these limitations and the likelihood that some relevant publications were missed, a
500 large collection of literature was sourced over a nine-year period, which we believe fairly reflects
501 the spread of theories used in care-leaving studies.
502 All the articles included in this review mentioned theory. We do not know how many articles on
503 care-leaving did not mention theory, and thus are unable to say what percentage of care-leaving
504 studies incorporated a theoretical lens of some sort. For that, an exhaustive review of all care-
505 leaving studies would be required, which was beyond the scope of our study.

506

# 507 Discussion and recommendations

508 In 2006, Stein raised concerns about the limited use of theory to inform care-leaving research. This
509 clarion call resounded and was taken up by many scholars working in the field of care-leaving. Of
510 the three theories Stein recommended as having potential to explicate the care-leaving process,
511 resilience is the front runner, with attachment also being well utilised. Focal theory lags considerably,
512 though studies using it provide helpful insights into the pile-up of challenges that care-leavers face
513 and have useful implications for practice. By contrast, life course theory, which Stein mentions in
514 passing in the second last paragraph of his paper, is currently the second most frequently used
515 theoretical framework for explaining care-leaving.
516 The emphasis on theory-informed research does not mean that empirical descriptive research is not
517 useful. It provides useful data on patterns that shape research, practice, policy and resource
518 allocation. However, research that is grounded in theory has the potential to illuminate the
519 experience of leaving care and the design of interventions to facilitate care-leaving. While this study
520 does not address the question of whether theory use is increasing, two other scoping reviews
521 provide evidence suggestive of an increase in theory-informed care-leaving research over time.
522 What this study distinctively shows is the diversity in the way theories are utilised in care-leaving
523 research. Over half of the studies that mentioned theory used theory extensively to inform and drive
524 the research questions, study design and interpretation of data, including recommendations for
525 policy and practice. This is encouraging, as such research not only uses theory rigorously, but may
526 also contribute to building and developing those theories.
527 However, a fifth of studies were theory informed, meaning some use of theory was made, at the
528 front or back of the study, but not pulled all the way through from conceptualisation to
529 recommendations. And a quarter of studies mentioned theories only in passing, which we termed
530 theory-alluded studies. There were studies that were driven primarily by one theory, and that also
531 utilised other theories in an informed or utilised fashion. The studies reviewed utilised, on average
532 1.4 theories each, suggesting that while some studies focused in on one theory, others may have
533 used two or more theories to weave together a multifaceted theoretical construction of leaving care.
534 It appears that many, if not most, of the theories used in three or more studies construct care-leaving
535 as a person-in-environment interaction. Some theories, that may be thought of as individual, such as
536 resilience, attachment, agency and focal, are utilised in contextualised ways, foregrounding the
537 interactions between care-leavers and their social environment. At very least, they are located in
538 interpersonal relationships, but frequently they are extended further into interactions with the
539 systems and structures of society.
540 Other theories, however, have a distinctively contextual and environmental focus, such as social
541 ecological theory, developmental social welfare and sustainable livelihoods, though with the
542 exception of ecological theory these are seldom used. Some other theories lean towards critical
543 theory, such as intersectionality. But others, such as anti-black racism, critical disability, ethic of
544 care, feminism, Marxism, minority stress, precarity and social justice, have been used only once or
545 twice over the past nine years. It will be good to see structural, systemic, critical and rights-oriented
546 theories enjoying greater use in the years to come.
547 Encouragingly, three quarters of the studies in this review used theory to inform practice
548 recommendations. The extent to which theory informed these recommendations varies from a token
549 nod to theory to a rigorous application of theory into care-leaving practice, but given Stein’s [21]
550 concern about “the poverty of theory”, even some use of theory to inform practice, and not only
551 data, is encouraging.
552 What remains sparse are theories specifically developed for care-leaving. Only one viable theory,
553 tested across more than one study, has emerged and it has not gained traction among other scholars.
554 It is, arguably, not necessary to construct new theories if existing theories can be customised or
555 applied to care-leaving. Nevertheless, theory development may prove helpful, not only for care-
556 leavers, but for all young people transitioning from childhood into adulthood.

557

# 558 Conclusion

559 In conclusion, we wish to celebrate the care-leaving scholarly community’s attention to Stein’s
560 seminal call almost 20 years ago. It does appear that care-leaving research is making greater use of
561 theory and when they do, theory is used to drive the research, rather than merely to inform it or to
562 briefly allude to it. This is leading to richer and more experience-near explanatory narratives about
563 the journey of leaving care, which should inform and strengthen care-leaving practice.
564 We suggest the following areas of future growth: (1) an increase in theory-driven research and a
565 decrease in theory-alluded research, so that theory is used purposefully and rigorously to make
566 sense of the care-leaving experience; (2) an increase in the use of contextual, systemic, rights-
567 oriented and critical theories, particularly given the increasingly fractured society that many care-
568 leavers transition into; and (3) an increase in theory building for care-leaving.

569

# 570 Acknowledgements

571 Honouring the contribution of care-experienced people: We stand with those who are care-
572 experienced. We acknowledge and respect their stories. We thank them for sharing their expertise
573 and wisdom and their extraordinary contributions to research, policy and practice. Learning from
574 their lived experience and working together towards improving care systems for future generations
575 is a privilege.

576

### 577 References

578 1.	United Nations. Guidelines for the alternative care of children. Geneva, Switzerland: United
579 Nations General Assembly; 2010.
580 2.	RSA. Children's Amendment Act, No 17 of 2022. Pretoria, South Africa: Government
581 Printers; 2022.
582 3.	Mendes P, Johnson G, Moslehuddin B. Young people leaving state out-of-home care.
583 Melbourne, Australia: Australian Scholarly Publishing; 2011.
584 4.	Collins ME. Macro perspectives on youths aging out of foster care. Washington DC:
585 National Association of Social Workers; 2015.
586 5.	Van Breda AD, Munro ER, Gilligan R, Anghel R, Harder A, Incarnato M, et al. Extended
587 care: Global dialogue on policy, practice and research. Children and Youth Services Review.
588 2020;119:105596. doi: 10.1016/j.childyouth.2020.105596
589 6.	Strahl B, van Breda AD, Mann-Feder V, Schröer W. A multinational comparison of care-
590 leaving policy and legislation. Journal of International and Comparative Social Policy.
591 2021;37(1):34-49. doi: 10.1017/ics.2020.26
592 7.	Dixon J, Stein M. Leaving care: Throughcare and aftercare in Scotland. London: Jessica
593 Kingsley; 2005.
594 8.	Mann-Feder VR, Goyette M, editors. Leaving care and the transition to adulthood:
595 International contributions to theory, research, and practice. Oxford, UK: Oxford University Press;
596 2019.
597 9.	Stein M, Munro ER, editors. Young people's transitions from care to adulthood:
598 International research and practice. London: Jessica Kingsley Publishers; 2008.
599 10.	Keller S, Oterholm I, Paulsen V, Van Breda AD, editors. Living on the edge: Innovative
600 research on leaving care and transitions to adulthood: Policy Press; 2024.
601 11.	Mendes P, Snow P, editors. Young people transitioning from care: International research,
602 policy and practice. London: Palgrave; 2016.
603 12.	Van Breda AD, Frimpong-Manso K. Editorial: Leaving care in Africa. Emerging
604 Adulthood. 2020;8(1):3-5. doi: 10.1177/2167696819895398
605 13.	Stein M. Young people's transitions from care to adulthood in European and postcommunist
606 Eastern European and Central Asian societies. Australian Social Work. 2014;67(1):24-38. doi:
607 10.1080/0312407X.2013.836236
608 14.	Dickens LF. One-year outcomes of youth exiting a residential care facility in South Africa.
609 Child & Family Social Work. 2018;23(4):558-65. doi: 10.1111/cfs.12411
610 15.	Kääriälä A, Hiilamo H. Children in out-of-home care as young adults: A systematic review
611 of outcomes in the Nordic countries. Children and Youth Services Review. 2017;79:107-14. doi:
612 10.1016/j.childyouth.2017.05.030
613 16.	Okpych N, Courtney M. Longitudinal analyses of educational outcomes for youth
614 transitioning out of care in the US: Trends and influential factors. Oxford Review of Education.
615 2019;45(4):461-80. doi: 10.1080/03054985.2019.1592747
616 17.	Marion É, Paulsen V. The transition to adulthood from care: A review of current research.
617 In: Mann-Feder VR, Goyette M, editors. Leaving care and the transition to adulthood: International
618 contributions to theory, research and practice. Oxford, UK: Oxford University Press; 2019. p. 107-
619 30.
620 18.	Ainsworth F, Hansen P. Family foster care: Can it survive the evidence? Children Australia.
621 2014;39(2):87-92. doi: 10.1017/cha.2014.5
622 19.	McSherry D. Remembering what the Big Friendly Giants said: To understand outcomes,
623 you first need to understand context. Children Australia. 2018;43(2):91-4. doi: 10.1017/cha.2018.24
624 20.	Rutter M. Children in substitute care: Some conceptual considerations and research
625 implications. Children and Youth Services Review. 2000;22(9):685-703. doi: 10.1016/S0190-
626 7409(00)00116-X
627 21.	Stein M. Young people aging out of care: The poverty of theory. Children and Youth
628 Services Review. 2006;28(4):422-34. doi: 10.1016/j.childyouth.2005.05.005
629 22.	Mann-Feder VR. How can I be a real adult? Develomental theory as a framework for
630 practice, policy, and research on care leaving. In: Mann-Feder VR, Goyette M, editors. Leaving
631 care and the transition to adulthood: International contributions to theory, research and practice.
632 Oxford, UK: Oxford University Press; 2019. p. 9-30.
633 23.	Goyette M. Social networks and social support in the transition to adulthoood: A reflection
634 on social integration processes. In: Mann-Feder VR, Goyette M, editors. Leaving care and the
635 transition to adulthood: International contributions to theory, research and practice. Oxford, UK:
636 Oxford University Press; 2019. p. 31-50.
637 24.	Gilligan R. Resilience, transitions, and youth leaving care. In: Mann-Feder VR, Goyette M,
638 editors. Leaving care and the transition to adulthood: International contributions to theory, research
639 and practice. Oxford, UK: Oxford University Press; 2019. p. 51-68.
640 25.	Munro E. Reflections on upholding the rights of youth leaving out-of-home care. In: Mann-
641 Feder VR, Goyette M, editors. Leaving care and the transition to adulthood: International
642 contributions to theory, research and practice. Oxford, UK: Oxford University Press; 2019. p. 69-
643 86.
644 26.	Page MJ, McKenzie JE, Bossuyt PM, Boutron I, Hoffmann TC, Mulrow CD, et al. The
645 PRISMA 2020 statement: An updated guideline for reporting systematic reviews. Systematic
646 Reviews. 2021;10(1):89. doi: 10.1186/s13643-021-01626-4
647 27.	Munn Z, Peters MDJ, Stern C, Tufanaru C, McArthur A, Aromataris E. Systematic review
648 or scoping review? Guidance for authors when choosing between a systematic or scoping review
649 approach. BMC Medical Research Methodology. 2018;18(1):143. doi: 10.1186/s12874-018-0611-x
650 28.	Van Breda AD. Journey towards independent living: A grounded theory investigation of
651 leaving the care of Girls & Boys Town South Africa. Journal of Youth Studies. 2015;18(3):322-37.
652 doi: 10.1080/13676261.2014.963534
653 29.	Shamseer L, Moher D, Clarke M, Ghersi D, Liberati A, Petticrew M, et al. Preferred
654 reporting items for systematic review and meta-analysis protocols (PRISMA-P) 2015: Elaboration
655 and explanation. BMMJ. 2015;349:g7647. doi: 10.1136/bmj.g7647
656 30.	Edwards T, Chowdhury R, Laylor A, Parada H, King B. Pushed, dropped, or fleeing from
657 care: The narratives and adultification of black youth who have aged out of Ontario’s child welfare
658 system. Child & Youth Services. 2023:Advance online publication. doi:
659 10.1080/0145935X.2023.2173565
660 31.	Appleton P. Anchors for deliberation and shared deliberation: Understanding planning in
661 young adults transitioning from out-of-home care. Qualitative Social Work. 2020;19(5-6):1130-46.
662 doi: 10.1177/1473325019869810
663 32.	Doucet M, Pratt H, Dzhenganin M, Read J. Nothing about us without us: Using
664 Participatory Action Research (PAR) and arts-based methods as empowerment and social justice
665 tools in doing research with youth ‘aging out’ of care. Child Abuse & Neglect. 2022;130:105358.
666 doi: 10.1016/j.chiabu.2021.105358
667 33.	Alderson H, Brown R, Copello A, Kaner E, Tober G, Lingam R, et al. The key therapeutic
668 factors needed to deliver behavioural change interventions to decrease risky substance use (drug
669 and alcohol) for looked after children and care leavers: a qualitative exploration with young people,
670 carers and front line workers. BMC Medical Research Methodology. 2019;19:1-12. doi:
671 10.1186/s12874-019-0674-3
672 34.	Neal D. Academic resilience and caring adults: The experiences of former foster youth.
673 Children and Youth Services Review. 2017;79:242-8. doi: 10.1016/j.childyouth.2017.06.005
674 35.	Bennwik I-HB, Oterholm I. Policy values related to support for care leavers with
675 disabilities. European Journal of Social Work. 2021;24(5):884-95. doi:
676 10.1080/13691457.2020.1751589
677 36.	Sulimani-Aidan Y, Achdut N, Zeira A, Benbenishty R. Psychological distress among care
678 leavers during the transition to adulthood: Risk and protective factors throughout their life course.
679 Child & Family Social Work. 2022;27(2):324-39. doi: 10.1111/cfs.12887
680 37.	Ehlke C, Schröer W. Fluid transitions: “Weak” constellations of participation in the process
681 of leaving care. Children and Youth Services Review. 2023;151:107036. doi:
682 10.1016/j.childyouth.2023.107036
683 38.	Golding F, Lewis A, McKemmish S, Rolan G, Thorpe K. Rights in records: A Charter of
684 Lifelong Rights in Childhood Recordkeeping in Out-of-Home Care for Australian and Indigenous
685 Australian children and care leavers. The International Journal of Human Rights. 2021;25(9):1625-
686 57. doi: 10.1080/13642987.2020.1859484
687 39.	Adley N, Jupp Kina V. Getting behind the closed door of care leavers: Understanding the
688 role of emotional support for young people leaving care. Child & Family Social Work.
689 2017;22(1):97-105. doi: 10.1111/cfs.12203
690 40.	Wilson DB. Youth in transition and foster care alumni as empowered consumers. Journal of
691 Family Social Work. 2020;23(5):411-30. doi: 10.1080/10522158.2019.1681335
692 41.	Tobolowsky BF, Scannapieco M, Aguiniga DM, Madden EE. Former foster youth
693 experiences with higher education: Opportunities and challenges. Children and Youth Services
694 Review. 2019;104:104362. doi: <https://doi.org/10.1016/j.childyouth.2019.05.039>
695 42.	Mendes P, Saunders B, Baidawi S. Indigenous young people transitioning from out-of-home
696 care (OOHC) in Victoria, Australia: The perspectives of workers in indigenous-specific and non-
697 indigenous non-government services. International Indigenous Policy Journal. 2016;7(3). doi:
698 10.18584/iipj.2016.7.3.2
699 43.	Adeboye TK, Guerreiro MdD, Höjer I. Unveiling the experiences of young people in foster
700 care: Perspectives from Portugal and Nigeria. International Social Work. 2019;62(1):433-46. doi:
701 10.1177/0020872817731147
702 44.	Van Breda AD, Pinkerton J. Raising African voices in the global dialogue on care-leaving
703 and emerging adulthood. Emerging Adulthood. 2020;8(1):6-15. doi: 10.1177/2167696819870356
704 45.	Houston S. Dialectical critical realism, transformative change and social work. Critical and
705 Radical Social Work. 2023;11(1):27-41. doi: 10.1332/204986021X16455451510551
706 46.	Nsonwu MB, Dennison S, Long J. Foster care chronicles: Use of the arts for teens aging out
707 of the foster care system. Journal of Creativity in Mental Health. 2015;10(1):18-33. doi:
708 10.1080/15401383.2014.935546
709 47.	Carey C. Coping with stigma: Experiences and responses of former youth in care.
710 Qualitative Sociology Review. 2023;19(3):26-51. doi: 10.18778/1733-8077.19.3.02
711 48.	Refaeli T, Zeira A, Benbenishty R. Ten years later: Satisfaction with intimate relationships
712 and parenthood of young people aging out of residential care. Child Abuse & Neglect.
713 2022;129:105635. doi: 10.1016/j.chiabu.2022.105635
714 49.	Couvrette A, Lanctôt N. Between parental efficacy and parental distress: How early
715 maladaptive schemas contribute to the motherhood experience of young mothers who have aged out
716 of care. Adoption & Fostering. 2017;41(2):170-87. doi: 10.1177/0308575916662162
717 50.	Kennedy TD, Flach Y, Detullio D, Millen DH, Englebert N, Edmonds WA. Exploring
718 emotional intelligence and IQ as predictors of success of foster care alumni. Journal of Child and
719 Family Studies. 2019;28(12):3286-95. doi: 10.1007/s10826-019-01503-8
720 51.	Østergaard J. Misfitting feelings: Young care leavers’ emotional work during the transition
721 to adulthood. Child Care in Practice. 2023;29(3):278-96. doi: 10.1080/13575279.2023.2167809
722 52.	Bromark K, Knutsson O, Spånberger Weitz Y. Co-designing a dynamic tool to enhance
723 participation for young people: A participatory design project with young service users and social
724 workers. Children and Youth Services Review. 2023;147:106856. doi:
725 10.1016/j.childyouth.2023.106856
726 53.	Törrönen M, Munn-Giddings C, Vornanen R. Existential well-being among young people
727 leaving care: Self-feeling, self-realisation, and belonging. Ethics and Social Welfare.
728 2023;17(3):295-311. doi: 10.1080/17496535.2022.2140176
729 54.	Goldfarb KE. ‘Self-responsibility’ and the politics of chance: Theorizing the experience of
730 Japanese child welfare. Japanese Studies. 2016;36(2):173-89. doi:
731 10.1080/10371397.2016.1208531
732 55.	Karki S, Ryynänen O-P, Salokekkilä P, Häggman-Laitila A. Bayesian analysis of the factors
733 explaining the disruptive behaviour of care leavers: A retrospective document analysis. Children
734 and Youth Services Review. 2023;155:107174. doi: 10.1016/j.childyouth.2023.107174
735 56.	Sulimani-Aidan Y. Left behind: How to encourage biological parents' involvement in their
736 children's group homes. Child & Family Social Work. 2018;23(2):180-7. doi:
737 <https://doi.org/10.1111/cfs.12397>
738 57.	Font SA, Berger LM, Cancian M, Noyes JL. Permanency and the educational and economic
739 attainment of former foster children in early adulthood. American Sociological Review.
740 2018;83(4):716-43. doi: 10.1177/0003122418781791
741 58.	Berejena Mhongera P. Preparing for successful transitions beyond institutional care in
742 Zimbabwe: Adolescent girls’ perspectives and programme needs. Child Care in Practice.
743 2017;23(4):372-88. doi: 10.1080/13575279.2016.1215291
744 59.	Staines J, Fitzpatrick C, Shaw J, Hunter K. ‘We need to tackle their well being first’:
745 Understanding and supporting care-experienced girls in the youth justice system. Youth Justice.
746 2023:Advance online publication. doi: 10.1177/14732254231191977
747 60.	Van Audenhove S, Vander Laenen F. Future expectations of young people leaving youth
748 care in Flanders: The role of personal and social capital in coping with expected challenges. Child
749 & Family Social Work. 2017;22(1):256-65. doi: 10.1111/cfs.12233
750 61.	Van Breda AD. Patterns of criminal activity among residential care-leavers in South Africa.
751 Children and Youth Services Review. 2020;109:104706. doi: 10.1016/j.childyouth.2019.104706
752 62.	Kenny K. The educational experiences of children in care across five decades: A new
753 perspective on the education of looked after children in the UK. Adoption & Fostering.
754 2023;47(1):22–39. doi: 10.1177/03085759231157415
755 63.	Klodnick VV, Samuels GM. Building home on a fault line: Aging out of child welfare with
756 a serious mental health diagnosis. Child & Family Social Work. 2020;25(3):704-13. doi:
757 10.1111/cfs.12747
758 64.	Sulimani-Aidan Y, Melkman E, Hellman CM. Nurturing the hope of youth in care: The
759 contribution of mentoring. American Journal of Orthopsychiatry. 2019;89(2):134-43. doi:
760 10.1037/ort0000320
761 65.	Modi K, Kasana S, Azam A, Madhavan L. Education, poverty and social exclusion:
762 Assessment of youth leaving care. SN Social Sciences. 2021;1(4):82. doi: 10.1007/s43545-021-
763 00102-z
764 66.	Achdut N, Benbenishty R, Zeira A. Labor force attachment among care leavers in Israel: A
765 longitudinal cross-sequential between-groups design. Child Abuse & Neglect. 2022;133:105860.
766 doi: 10.1016/j.chiabu.2022.105860
767 67.	Miller JJ, Chih M-Y, Washington E. Conceptualizing a mobile app for foster youth
768 transitioning to adulthood: A mixed-method approach. Journal of Technology in Human Services.
769 2016;34(2):145-70. doi: 10.1080/15228835.2015.1108260
770 68.	Artamonova A, Guerreiro MdD, Höjer I. Time and context shaping the transition from out-
771 of-home care to adulthood in Portugal. Children and Youth Services Review. 2020;115:105105.
772 doi: 10.1016/j.childyouth.2020.105105
773 69.	Rambajue R, O’Connor C. Intersectional individualization: Toward a theoretical framework
774 for youth transitioning out of the child welfare system. Journal of Public Child Welfare.
775 2022;16(2):156-76. doi: 10.1080/15548732.2020.1856284
776 70.	Bennwik I-HB, Oterholm I, Kelly B. ‘My disability was my own responsibility’: An
777 institutional ethnography of the transitional experiences of disabled young people leaving care.
778 Children and Youth Services Review. 2023;146:106813. doi: 10.1016/j.childyouth.2023.106813
779 71.	Disney T, Walker C. Young people leaving care and institutionalised vulnerability in the
780 Russian Federation. Children and Youth Services Review. 2023;155:107225. doi:
781 10.1016/j.childyouth.2023.107225
782 72.	Jensen SG. The rebuilding of fragmented memories, broken families and rootless selves
783 among Danish care leavers. Journal of Family History. 2021;46(1):77–91. doi:
784 10.1177/0363199020967582
785 73.	Gomez RJ, Ryan TN, Norton CL, Jones C, Galán-Cisneros P. Perceptions of learned
786 helplessness among emerging adults aging out of foster care. Child and Adolescent Social Work
787 Journal. 2015;32(6):507-16. doi: 10.1007/s10560-015-0389-1
788 74.	Lemus D, Farruggia SP, Germo G, Chang ES. The plans, goals, and concerns of pre-
789 emancipated youth in foster care. Children and Youth Services Review. 2017;78:48-55. doi:
790 10.1016/j.childyouth.2017.05.003
791 75.	Anghel R. From ‘the New Man’ to care-leaver activists: Communist and contemporary
792 discourses shaping 50 years of leaving care in Romania. Child & Family Social Work.
793 2021;26(2):258-69. doi: 10.1111/cfs.12819
794 76.	Sulimani-Aidan Y, Melkman E, Greeson JKP. The contribution of mentoring to the life
795 skills of youth leaving care in Israel. Child & Family Social Work. 2020;25(2):345-54. doi:
796 10.1111/cfs.12690
797 77.	Paul JC. Exploring support for LGBTQ youth transitioning from foster care to emerging
798 adulthood. Children and Youth Services Review. 2020;119:105481. doi:
799 10.1016/j.childyouth.2020.105481
800 78.	Gross JP, Geiger JM, Uhls E, King G. The relationship between financial aid and
801 postsecondary completion among youth formerly in foster care. Child and Adolescent Social Work
802 Journal. 2023;40(2):207-19. doi: 10.1007/s10560-022-00884-y
803 79.	Wilson JZ, Golding F. Latent scrutiny: personal archives as perpetual mementos of the
804 official gaze. Archival Science. 2016;16(1):93-109. doi: 10.1007/s10502-015-9255-3
805 80.	Cleaver K. Acknowledging the struggle: Policy changes for state care leaving provisions.
806 Aotearoa New Zealand Social Work Review. 2016;28(2):22-31. doi: 10.11157/anzswj-
807 vol28iss2id221
808 81.	Barboza GE, Valentine R. A network analysis of post-traumatic stress among youth aging
809 out of the foster care system. Children and Youth Services Review. 2022;140:106589. doi:
810 10.1016/j.childyouth.2022.106589
811 82.	Storø J. Leaving care in Norway in a historic and current perspective: As a function of the
812 Nordic welfare model. Child & Family Social Work. 2021;26(2):240-7. doi: 10.1111/cfs.12805
813 83.	Gabriel T. The child: Object or subject of child care? Children and Youth Services Review.
814 2023;150:107022. doi: 10.1016/j.childyouth.2023.107022
815 84.	Paul-Ward A, Lambdin-Pattavina CA. New roles for occupational therapy to promote
816 independence among youth aging out of foster care. The American Journal of Occupational
817 Therapy. 2016;70(3):1-5. doi: 10.5014/ajot.2016.017426
818 85.	Kearney KS, Naifeh Z, Hammer T, Cain A. "Family" ties for foster alumni in college: An
819 open systems consideration. Review of Higher Education. 2019;42(2):793-824. doi:
820 10.1353/rhe.2019.0015
821 86.	Nurcombe-Thorne A, Nadesan VS, Van Breda AD. Experiences of ‘I’ and ‘we’ among
822 former looked-after children in South Africa. Child & Family Social Work. 2018;23(4):640-8. doi:
823 10.1111/cfs.12456
824 87.	Stevenson BJ. Developing a career counseling intervention program for foster youth.
825 Journal of Employment Counseling. 2017;54(2):75-86. doi: 10.1002/joec.12055
826 88.	Rice J, Mullineux J, Killick C. Female care leavers experience of the staff-child relationship
827 while living in an intensive support children's home in Northern Ireland. Child Care in Practice.
828 2022;28(1):4-19. doi: 10.1080/13575279.2019.1693979
829 89.	Sims-Schouten W, Hayden C. Mental health and wellbeing of care leavers: Making sense of
830 their perspectives. Child & Family Social Work. 2017;22(4):1480-7. doi: 10.1111/cfs.12370
831 90.	Törrönen M, Munn-Giddings C, Gavriel C, Morris D. Emotional participation of young
832 adults starting their independent living. Nordic Social Work Research. 2018;8(sup1):65-78. doi:
833 10.1080/2156857X.2018.1489883
834 91.	Driscoll J. Strangers and estrangement: Young people’s renegotiations of birth and foster
835 family relationships as they transition out of care and the implications for the state as parent.
836 Children's Geographies. 2019;17(5):539-51. doi: 10.1080/14733285.2017.1422209
837 92.	Mertz M, Andersen SH. The hidden cost of foster-care: new evidence on the inter-
838 generational transmission of foster-care experiences. British Journal of Social Work.
839 2017;47(5):1377-93. doi: 10.1093/bjsw/bcw132
840 93.	Raithel J, Yates M, Dworsky A, Schretzman M, Welshimer W. Partnering to leverage
841 multiple data sources: Preliminary findings from a supportive housing impact study. Child Welfare.
842 2015;94(1):73-85
843 94.	Luyten I, Nuytiens A, Christiaens J, Dumortier E. Voicing young and older adult care-
844 leavers in Belgium: How the experience of being in care shapes narratives of the self. Longitudinal
845 & Life Course Studies. 2018;9(1):80-100. doi: 10.14301/llcs.v9i1.456
846 95.	Olsson TM, Blakeslee J, Bergström M, Skoog T. Exploring fit for the cultural adaptation of
847 a self-determination model for youth transitioning from out-of-home care: A comparison of a
848 sample of Swedish youth with two samples of American youth in out-of-home care. Children and
849 Youth Services Review. 2020;119:105484. doi: 10.1016/j.childyouth.2020.105484
850 96.	Forenza B, Lardier DT. Sense of community through supportive housing among foster care
851 alumni. Child Welfare. 2017;95(2):91-115
852 97.	Butler K, Benoit C. Citizenship practices among youth who have experienced government
853 care. Canadian Journal of Sociology (Online). 2015;40(1):25-49. doi:
854 10.2307/canajsocicahican.40.1.25
855 98.	Swan M, Holt S, Kirwan G. ‘Who do I turn to if something really bad happens?’ Key
856 working and relationship-based practice in residential child care. Journal of Social Work Practice.
857 2018;32(4):447-61. doi: 10.1080/02650533.2018.1503161
858 99.	Park K, Courtney ME. Mitigating risks of incarceration among transition-age foster youth:
859 considering domains of social bonds. Child & Adolescent Social Work Journal. 2023;40(4):473-86.
860 doi: 10.1007/s10560-022-00891-z
861 100.	Gwenzi GD, Ringson J. “Why would they call me an orphan when I have parents”: Care
862 leavers’ experiences of labelling and stereotyping whilst living in residential care facilities in
863 Zimbabwe. New Ideas in Psychology. 2023;68:100968. doi: 10.1016/j.newideapsych.2022.100968
864 101.	Bond S. Care leaving in South Africa: An international and social justice perspective.
865 Journal of International and Comparative Social Policy. 2018;34(1):76-90. doi:
866 10.1080/21699763.2017.1413994
867 102.	Lee JS, Gimm G, Mohindroo M, Lever L. Assessing homelessness and incarceration among
868 youth aging out of foster care, by type of disability. Child and Adolescent Social Work Journal.
869 2023;40(6):743-59. doi: 10.1007/s10560-022-00817-9
870 103.	Sundly A, Keating M, Effiong A, Saif AO. Neuroscientific evidence and care leaving: a
871 multidisciplinary critical commentary. International Journal of Child, Youth & Family Studies.
872 2022;14(1):30-46. doi: 10.18357/ijcyfs141202321283
873 104.	Gwenzi GD. Provision of transitional housing: A socially sustainable solution for care
874 leavers in Zimbabwe. Practice. 2023;35(2):103-20. doi: 10.1080/09503153.2022.2083100
875 105.	Neagu M, Sebba J. Who do they think they are: Making sense of self in residential care,
876 foster care, and adoption. Children and Youth Services Review. 2019;105:104449. doi:
877 10.1016/j.childyouth.2019.104449
878 106.	Collins ME, Augsberger A. Impacts of policy changes on Care-Leaving Workers in a time
879 of Coronavirus: Comparative analysis of discretion and constraints. Journal of Comparative Policy
880 Analysis: Research and Practice. 2021;23(1):51-62. doi: 10.1080/13876988.2020.1841560
881 107.	Macleod G, Dallas-Childs R, Brough C, Toye M. 'She just got me’: Supporting care
882 experienced young people negotiating relationships and identities at school. Journal of Research in
883 Special Educational Needs. 2021;21(S1):25-35. doi: 10.1111/1471-3802.12543
884 108.	Thompson RW, Duppong Hurley K, Trout AL, Huefner JC, Daly DL. Closing the research
885 to practice gap in therapeutic residential care: Service provider–university partnerships focused on
886 evidence-based practice. Journal of Emotional and Behavioral Disorders. 2017;25(1):46-56. doi:
887 10.1177/1063426616686757
888 109.	Leathers SJ, Holtschneider C, Ludington M, Ross EV, Barnett JL. Mentoring, employment
889 assistance, and enhanced staff outreach for older youth in care: Outcomes from a randomized
890 controlled trial. Children and Youth Services Review. 2023;153:107095. doi:
891 10.1016/j.childyouth.2023.107095
892 110.	Storø J. Which transition concept is useful for describing the process of young people
893 leaving state care? A reflection on research and language. European Journal of Social Work
894 2017;20(5):770-81. doi: 10.1080/13691457.2016.1255879
895 111.	Söderqvist Å, Bülow PH, Sjöblom Y. “In Sweden work is more important than the culture,
896 actually!” The care leaving process for unaccompanied youths from the perspective of social
897 workers. Transnational Social Review. 2015;5(3):241-57. doi: 10.1080/21931674.2015.1082778
898 112.	Fusco RA, Kulkarni SJ. “Bedtime is when bad stuff happens”: Sleep problems in foster care
899 alumni. Children and Youth Services Review. 2018;95:42-8. doi: 10.1016/j.childyouth.2018.10.024
900 113.	Mølholt AK, Bengtsson TT, Frederiksen M. Navigating educational success: Modes of
901 expectation among care‐experienced young people. British Educational Research Journal.
902 2023:Advance online publication. doi: 10.1002/berj.3908
903 114.	Butterworth S, Mendes P, Flynn C. Young people leaving out-of-home care in Victoria,
904 Australia: An exploration of factors influencing positive transitions. Social Work & Policy Studies:
905 Social Justice, Practice and Theory. 2020;3(2):1-23
906 115.	Goemans M, Van Breda AD, Kessi S. Experiences of young people preparing to transition
907 out of cluster foster care in South Africa. Child and Adolescent Social Work Journal.
908 2021;38(2):227-37. doi: 10.1007/s10560-020-00704-1
909 116.	Gwenzi GD. Care leavers’ notions of the ‘ideal’ family in Zimbabwe. Journal of Family
910 Studies. 2023;29(3):1428-46. doi: 10.1080/13229400.2022.2058411
911 117.	Bengtsson TT, Mølholt A-K. Creation of belonging and non-belonging in the temporal
912 narratives of young people transitioning out of care in Denmark. Nordic Social Work Research.
913 2018;8(sup1):54-64. doi: 10.1080/2156857X.2018.1494034
914 118.	Smith GP, Darab S, Hartman Y. Exploring a sense of belonging for some Forgotten
915 Australians as they age. Community, Work & Family. 2022;25(2):137-51. doi:
916 10.1080/13668803.2020.1749030
917 119.	Bennwik I-HB, Oterholm I, Kelly B. ‘Disability is not a word we use’: Social workers'
918 professional judgements about support for disabled young people leaving care. Child & Family
919 Social Work. 2023;28(2):443-53. doi: 10.1111/cfs.12975
920 120.	Batista T, Johnson A, Friedmann LB. The effects of youth empowerment programs on the
921 psychological empowerment of young people aging out of foster care. Journal of the Society for
922 Social Work & Research. 2018;9(4):531-49. doi: 10.1086/700275
923 121.	Benaton T, Bowers-Brown T, Dodsley T, Manning-Jones A, Murden J, Nunn A, et al.
924 Reconciling care and justice in contesting social harm through performance and arts practice with
925 looked after children and care leavers. Children & Society. 2020;34(5):337-53. doi:
926 10.1111/chso.12370
927 122.	Uptin J, Hartung C. Living in a crowd: Young people’s counter-narratives of care in a Thai
928 orphanage. Children's Geographies. 2022:1-14. doi: 10.1080/14733285.2022.2033954
929 123.	Boddy J, McCarthy JR, Gillies V, Hooper C-A. Troubling meanings of "family" for young
930 people who have been in care: From policy to lived experience. Journal of Family Issues.
931 2019;40(16):2239-63. doi: 10.1177/0192513X18808564
932 124.	Gwenzi GD. Constructing the meaning of “family” in the context of out-of-home care: An
933 exploratory study on residential care leavers in Harare, Zimbabwe. Emerging Adulthood.
934 2020;8(1):54-63. doi: 10.1177/2167696818800846
935 125.	Steenbakkers A, Ellingsen IT, Steffie van der S, Grietens H. Psychosocial needs of children
936 in foster care and the impact of sexual abuse. Journal of Child and Family Studies.
937 2018;27(4):1324-35. doi: 10.1007/s10826-017-0970-7
938 126.	Steenbakkers A, van der Steen S, Grietens H, Ellingsen IT. Do foster parents and care
939 workers recognize the needs of youth in family foster care with a history of sexual abuse? Journal
940 of Child Sexual Abuse. 2018;27(7):811-31. doi: 10.1080/10538712.2018.1520378
941 127.	Gullo F, García-Alba L, Bravo A, del Valle JF. The psychosocial adjustment of care leavers
942 in their transition to adult independent living (El ajuste psicosocial de jóvenes extutelados en su
943 transición a la vida adulta independiente). International Journal of Social Psychology.
944 2023;38(1):35–65. doi: 10.1080/02134748.2022.2132747
945 128.	Refaeli T, Benbenishty R, Zeira A. Predictors of life satisfaction among care leavers: A
946 mixed-method longitudinal study. Children and Youth Services Review. 2019;99:146-55. doi:
947 10.1016/j.childyouth.2019.01.044
948 129.	Oterholm I, Paulsen V. Young people and social workers’ experience of differences between
949 child welfare services and social services. Nordic Social Work Research. 2018;8(sup1):19-29. doi:
950 10.1080/2156857X.2018.1450283
951 130.	Mendis K, Gardner F, Lehmann J. The education of children in out-of-home care. Australian
952 Social Work. 2015;68(4):483-96. doi: 10.1080/0312407X.2014.963134
953 131.	Mendis K, Lehmann J, Gardner F. Promoting academic success of children in care. British
954 Journal of Social Work. 2018;48(1):106-23. doi: 10.1093/bjsw/bcx029
955 132.	Stead H. Rite of passage for care leavers. Journal of Family Therapy. 2021;43(3):445-57.
956 doi: 10.1111/1467-6427.12350
957 133.	Glynn N. Understanding care leavers as youth in society: A theoretical framework for
958 studying the transition out of care. Children and Youth Services Review. 2021;121:105829. doi:
959 10.1016/j.childyouth.2020.105829
960 134.	Glynn N, Mayock P. Housing after care: Understanding security and stability in the
961 transition out of care through the lenses of liminality, recognition and precarity. Journal of Youth
962 Studies. 2023;26(1):80-97. doi: 10.1080/13676261.2021.1981838
963 135.	Huegler N, Ruch G. Risk, vulnerability and complexity: Transitional safeguarding as a
964 reframing of binary perspectives. Practice. 2022;34(1):25-39. doi: 10.1080/09503153.2021.1932787
965 136.	Natalier K, Johnson G. No home away from home: A qualitative study of care leavers'
966 perceptions and experiences of ‘home’. Housing Studies. 2015;30(1):123-38. doi:
967 10.1080/02673037.2014.943698
968 137.	Lee JS, Ballew KM. Independent living services, adjudication status, and the social
969 exclusion of foster youth aging out of care in the United States. Journal of Youth Studies.
970 2018;21(7):940-57. doi: 10.1080/13676261.2018.1435854
971 138.	Kind N, Seker S, d'Huart D, Bürgin D, Jenkel N, Boonmann C, et al. High-risk substance
972 use and psychosocial functioning in young adult care leavers: Findings from a 10-year follow-up
973 study. Children and Youth Services Review. 2023;155:107290. doi:
974 10.1016/j.childyouth.2023.107290
975 139.	Blakeslee JE. Measuring the support networks of transition-age foster youth: Preliminary
976 validation of a social network assessment for research and practice. Children and Youth Services
977 Review. 2015;52:123-34. doi: 10.1016/j.childyouth.2015.03.014
978 140.	Marion É, Paulsen V, Goyette M. Relationships matter: Understanding the role and impact
979 of social networks at the edge of transition to adulthood from care. Child & Adolescent Social
980 Work Journal. 2017;34(6):573-82. doi: 10.1007/s10560-017-0494-4
981 141.	Moodley R, Raniga T, Sewpaul V. Youth transitioning out of residential care in South

| 982 | Africa: Toward ubuntu and interdependent living. Emerging Adulthood. 2020;8(1):45-53. doi: |
| --- | --- |
| 983 | 10.1177/2167696818812603 |
| 984 | 142. Barratt C, Appleton P, Pearson M. Exploring internal conversations to understand the |
| 985 | experience of young adults transitioning out of care. Journal of Youth Studies. 2020;23(7):869-85. |
| 986 | doi: 10.1080/13676261.2019.1645310 |
| 987 | 143. Göbel S, Hadjar A, Karl U, Jäger JA. Agency and the school-to-work transition of care |
| 988 | leavers: A retrospective study of Luxembourgish young people. Children and Youth Services |
| 989 | Review. 2021;122. doi: 10.1016/j.childyouth.2020.105636 |
| 990 | 144. Gundersen T. Embedded in relations: Interactions as a source of agency and life |
| 991 | opportunities for care-experienced young adults. Children & Society. 2021;35(5):680-93. doi: |
| 992 | 10.1111/chso.12434 |
| 993 | 145. Hung I, Appleton P. To plan or not to plan: The internal conversations of young people |
| 994 | leaving care. Qualitative Social Work. 2016;15(1):35-54. doi: 10.1177/1473325015577408 |
| 995 | 146. Van Breda AD. The roles of agency and structure in facilitating the successful transition out |
| 996 | of care and into independent living. Social Work Practitioner-Researcher. 2016;28(1):36-52. doi: |
| 997 | 10.25159/2415-5829/1349 |
| 998 | 147. Bogdanova E. Russian SOS Children's Villages and deinstitutionalisation reform: Balancing |
| 999 | between institutional and family care. Zhurnal Issledovanii Sotsialnoi Politiki [The Journal of |
| 1000 | Social Policy Studies]. 2017;15(3):395-406. doi: 10.17323/727-0634-2017-15-3-395-406 |
| 1001 | 148. Ferguson L. "Could an increased focus on identity development in the provision of |
| 1002 | children’s services help shape positive outcomes for care leavers?" A literature review. Child Care |
| 1003 | in Practice. 2018;24(1):76-91. doi: 10.1080/13575279.2016.1199536 |
| 1004 | 149. Goyette M, Blanchet A, Esposito T, Delaye A. The role of placement instability on |
| 1005 | employment and educational outcomes among adolescents leaving care. Children and Youth |
| 1006 | Services Review. 2021;131:106264. doi: 10.1016/j.childyouth.2021.106264 |

1007	150.	Heyman JC, White-Ryan L, Kelly P, Farmer GL, Leaman TL, Davis HJ. Voices about foster

| 1008 | care: The value of trust. Children and Youth Services Review. 2020;113:104991. doi: |
| --- | --- |
| 1009 | 10.1016/j.childyouth.2020.104991 |
| 1010 | 151. Hyde A, Fullerton D, Lohan M, Dunne L, Macdonald G. Young people’s views on the |
| 1011 | impact of care experiences on their ability to form positive intimate relationships. Adoption & |
| 1012 | Fostering. 2017;41(3):242-53. doi: 10.1177/0308575917714375 |
| 1013 | 152. Kelly P, Heyman J, Zhai F, Salazar A. Social and emotional supports during college years: |
| 1014 | Associations with post-college outcomes among alumni of foster care. Child and Adolescent Social |
| 1015 | Work Journal. 2023;40(5):607-21. doi: 10.1007/s10560-021-00806-4 |
| 1016 | 153. Malvaso C, Delfabbro P, Hackett L, Mills H. Service approaches to young people with |
| 1017 | complex needs leaving out-of-home care. Child Care in Practice. 2016;22(2):128-47. doi: |
| 1018 | 10.1080/13575279.2015.1118016 |
| 1019 | 154. Mayall H, O'Neill T, Worsley A, Devereux R, Ward S, Lynch D. The experiences of care |
| 1020 | leavers (post-care adults) in social work education. Social Work Education. 2015;34(2):151-64. doi: |
| 1021 | 10.1080/02615479.2014.962019 |
| 1022 | 155. Pasli F, Aslantürk H. Examining the family belonging of adults with institutional care |
| 1023 | experience in childhood. Asia Pacific Journal of Social Work and Development. 2023:Advance |
| 1024 | online publication. doi: 10.1080/02185385.2023.2233470 |
| 1025 | 156. Richardson V, Boylan J, Brammer A. Contact, welfare and children in care: revisiting the |
| 1026 | significance of birth family relationships after finding significant harm. Journal of Social Welfare & |
| 1027 | Family Law. 2017;39(1):67-82. doi: 10.1080/09649069.2016.1275124 |
| 1028 | 157. Sulimani-Aidan Y. Assets and pathways in achieving future goals of residential care alumni. |
| 1029 | Children and Youth Services Review. 2018;89:71-6. doi: 10.1016/j.childyouth.2018.04.023 |
| 1030 | 158. Valset K. Investigating the link between school performance, aftercare and educational |
| 1031 | outcome among youth ageing out of foster care: a Norwegian nationwide longitudinal cohort study. |
| 1032 | Nordic Social Work Research. 2018;8(sup1):79-93. doi: 10.1080/2156857X.2018.1457557 |

1033	159.	Ward S, Devereux R, Mayall H, O'Neill T, Worsley A. Care leavers on social work courses:

| 1034 | A study of identity management. Child & Family Social Work. 2017;22(1):339-48. doi: |
| --- | --- |
| 1035 | 10.1111/cfs.12244 |
| 1036 | 160. Sulimani-Aidan Y. Present, protective, and promotive: Mentors’ roles in the lives of young |
| 1037 | adults in residential care. American Journal of Orthopsychiatry. 2018;88(1):69-77. doi: |
| 1038 | 10.1037/ort0000235 |
| 1039 | 161. Sulimani-Aidan Y, Paldi Y. Youth perspectives on parents' involvement in residential care |
| 1040 | in Israel. Journal of Social Work. 2020;20(1):64-82. doi: 10.1177/1468017318793619 |
| 1041 | 162. Dickens L. One‐year outcomes of youth exiting a residential care facility in South Africa. |
| 1042 | Child & Family Social Work. 2018;23(4):558-65. doi: 10.1111/cfs.12411 |
| 1043 | 163. Van Breda AD, Dickens LF. The contribution of resilience to one-year independent living |
| 1044 | outcomes of care-leavers in South Africa. Children and Youth Services Review. 2017;83:264-73. |
| 1045 | doi: 10.1016/j.childyouth.2017.11.009 |
| 1046 | 164. Dutta S. Experiences of young Indian girls transiting out of residential care homes. Asian |
| 1047 | Social Work and Policy Review. 2017;11(1):16-29. doi: 10.1111/aswp.12107 |
| 1048 | 165. Dutta S. Preparation for social reintegration among young girls in residential care in India. |
| 1049 | International Journal of Child, Youth & Family Studies. 2018;9(2):151-70. doi: |
| 1050 | 10.18357/ijcyfs92201818217 |
| 1051 | 166. Fernandez E, Delfabbro P, Ramia I, Kovacs S. Children returning from care: The |
| 1052 | challenging circumstances of parents in poverty. Children and Youth Services Review. |
| 1053 | 2019;97:100-11. doi: 10.1016/j.childyouth.2017.06.008 |
| 1054 | 167. Harder AT, Mann-Feder V, Oterholm I, Refaeli T. Supporting transitions to adulthood for |
| 1055 | youth leaving care: Consensus based principles. Children and Youth Services Review. |
| 1056 | 2020;116:105260. doi: 10.1016/j.childyouth.2020.105260 |

1057	168.	Harwick RM, Unruh D, Lindstrom L. Transition to adulthood for youth with disabilities

| 1058 | who experienced foster care: An ecological approach. Child Abuse & Neglect. 2020;99:104310. |
| --- | --- |
| 1059 | doi: 10.1016/j.chiabu.2019.104310 |
| 1060 | 169. January S-AA, Trout AL, Jacqueline H-DA, Duppong Hurley KL, Thompson RW. |
| 1061 | Perspectives on factors impacting youth’s reentry into residential care: An exploratory study. |
| 1062 | Journal of Child and Family Studies. 2018;27(8):2584-95. doi: 10.1007/s10826-018-1093-5 |
| 1063 | 170. Modi K, Prasad A, Mishra J. Assisting youth leaving care: Understanding Udayan Care's |
| 1064 | aftercare programme through the prism of ecological systems theory. Scottish Journal of Residential |
| 1065 | Child Care. 2018;17(1):1-24. doi: 10.17868/strath.00084531 |
| 1066 | 171. Pinkerton J. Exploring history in the social ecology of care leaving: Northern Ireland as |
| 1067 | illustration. Child & Family Social Work. 2021;26(2):270-9. doi: 10.1111/cfs.12804 |
| 1068 | 172. Bond S. Care leavers’ and their care workers’ views of preparation and aftercare services in |
| 1069 | the Eastern Cape, South Africa. Emerging Adulthood. 2020;8(1):26-34. doi: |
| 1070 | 10.1177/2167696818801106 |
| 1071 | 173. Bond S, Van Breda AD. Interaction between possible selves and the resilience of care- |
| 1072 | leavers in South Africa. Children and Youth Services Review. 2018;94:88-95. doi: |
| 1073 | 10.1016/j.childyouth.2018.09.014 |
| 1074 | 174. Dickens LF, Marx P. NEET as an outcome for care leavers in South Africa: The case of |
| 1075 | Girls and Boys Town. Emerging Adulthood. 2020;8(1):64-72. doi: 10.1177/2167696818805891 |
| 1076 | 175. Dziro C, Mtetwa E, Mukamuri B, Chikwaiwa BK. Challenges faced by western-modelled |
| 1077 | residential care institutions in preparing the residents for meaningful re-integration into society: A |
| 1078 | case study of a Harare-based children's home. Journal of Social Development in Africa. |
| 1079 | 2013;28(2):113-30 |
| 1080 | 176. Gypen L, Stas L, West D, Van Holen F, Vanderfaeillie J. Longitudinal outcomes of |
| 1081 | employment, income and housing for Flemish care leavers. Developmental Child Welfare. |
| 1082 | 2023;5(1):36-58. doi: 10.1177/25161032231157094 |

1083	177.	Pryce JM, Jones SL, Wildman A, Thomas A, Okrzesik K, Kaufka-Walts K. Aging out of

| 1084 | care in Ethiopia: Challenges and implications facing orphans and vulnerable youth. Emerging |
| --- | --- |
| 1085 | Adulthood. 2016;4(2):119-30. doi: 10.1177/2167696815599095 |
| 1086 | 178. Sulimani-Aidan Y. Future expectations as a source of resilience among young people |
| 1087 | leaving care. British Journal of Social Work. 2017;47(4):1111–27. doi: 10.1093/bjsw/bcw077 |
| 1088 | 179. Sulimani-Aidan Y. To dream the impossible dream: Care leavers' challenges and barriers in |
| 1089 | pursuing their future expectations and goals. Children and Youth Services Review. 2017;81:332-9. |
| 1090 | doi: 10.1016/j.childyouth.2017.08.025 |
| 1091 | 180. Sulimani-Aidan Y. Social networks during the transition to adulthood from the perspective |
| 1092 | of Israeli care leavers and their social workers. Children and Youth Services Review. |
| 1093 | 2020;115:105075. doi: 10.1016/j.childyouth.2020.105075 |
| 1094 | 181. Sulimani-Aidan Y, Melkman E. Risk and resilience in the transition to adulthood from the |
| 1095 | point of view of care leavers and caseworkers. Children and Youth Services Review. 2018;88:135- |
| 1096 | 40. doi: 10.1016/j.childyouth.2018.03.012 |
| 1097 | 182. Takele AM, Kotecho MG. Female care-leavers’ experiences of aftercare in Ethiopia. |
| 1098 | Emerging Adulthood. 2020;8(1):73-81. doi: 10.1177/2167696819868355 |
| 1099 | 183. Tyrell FA, Yates TM. Emancipated foster youth’s experiences and perceptions of the |
| 1100 | transition to adulthood. Journal of Youth Studies. 2018;21(8):1011-28. doi: |
| 1101 | 10.1080/13676261.2018.1441983 |
| 1102 | 184. Zeira A, Refaeli T, Achdut N, Benbenishty R. Economic self-sufficiency and the |
| 1103 | employment outcomes of care leavers: A 10-year follow up. Child & Family Social Work. |
| 1104 | 2023;28(3):635–45. doi: 10.1111/cfs.12990 |
| 1105 | 185. Sulimani-Aidan Y. Qualitative exploration of supporting figures in the lives of emerging |
| 1106 | adults who left care compared with their noncare-leaving peers. Child & Family Social Work. |
| 1107 | 2019;24(2):247-55. doi: 10.1111/cfs.12609 |

1108	186.	Sulimani-Aidan Y. Challenges in the transition to adulthood of young-adult Arabs who

| 1109 | graduated from residential facilities in Israel. Children and Youth Services Review. |
| --- | --- |
| 1110 | 2020;113:104967. doi: 10.1016/j.childyouth.2020.104967 |
| 1111 | 187. Hollingworth K, Jackson S. Falling off the ladder: Using focal theory to understand and |
| 1112 | improve the educational experiences of young people in transition from public care. Journal of |
| 1113 | Adolescence. 2016;52:146-53. doi: 10.1016/j.adolescence.2016.08.004 |
| 1114 | 188. Munro ER, Simkiss D. Transitions from care to adulthood: messages to inform practice. |
| 1115 | Paediatrics and Child Health. 2020;30(5):175-9. doi: 10.1016/j.paed.2020.02.007 |
| 1116 | 189. Mupaku WM, Van Breda AD, Kelly B. Transitioning to adulthood from residential |
| 1117 | childcare during COVID-19: Experiences of young people with intellectual disabilities and/or |
| 1118 | autism spectrum disorder in South Africa. British Journal of Learning Disabilities. 2021;49(3):341- |
| 1119 | 51. doi: 10.1111/bld.12409 |
| 1120 | 190. Atwool N. Transition from care: Are we continuing to set care leavers up to fail in New |
| 1121 | Zealand? Children and Youth Services Review. 2020;113:104995. doi: |
| 1122 | 10.1016/j.childyouth.2020.104995 |
| 1123 | 191. Colbridge AK, Hassett A, Sisley E. “Who am I?” How female care leavers construct and |
| 1124 | make sense of their identity. SAGE Open. 2017;7(1). doi: 10.1177/2158244016684913 |
| 1125 | 192. Munford R, Sanders J. Negotiating and constructing identity: Social work with young |
| 1126 | people who experience adversity. British Journal of Social Work. 2015;45(5):1564-80. doi: |
| 1127 | bjsw/bcu059 |
| 1128 | 193. Storø J. To manage on one’s own after leaving care? A discussion of the concepts |
| 1129 | independence versus interdependence. Nordic Social Work Research. 2018;8(sup1):104-15. doi: |
| 1130 | 10.1080/2156857X.2018.1463282 |
| 1131 | 194. Bhargava R, Chandrashekhar R, Kansal S, Modi K. Young adults transitioning from |
| 1132 | institutional care to independent living: The role of aftercare support and services. Institutionalised |
| 1133 | Children Explorations and Beyond. 2018;5(2):168-87. doi: 10.1177/2349301120180207 |

1134	195.	McCormick M, Anthony J, Townsend ET. Fostering safer spaces: Retaining and

| 1135 | empowering SGM students with a lived history of foster care. Child & Adolescent Social Work |
| --- | --- |
| 1136 | Journal. 2023;40(2):255-70. doi: 10.1007/s10560-022-00889-7 |
| 1137 | 196. Hlungwani J, Van Breda AD. Female care leavers’ journey to young adulthood from |
| 1138 | residential care in South Africa: Gender-specific psychosocial processes of resilience. Child & |
| 1139 | Family Social Work. 2020;25(4):915-23. doi: 10.1111/cfs.12776 |
| 1140 | 197. Van Breda AD, Hlungwani J. Journey towards independent living: Resilience processes of |
| 1141 | women leaving residential care in South Africa. Journal of Youth Studies. 2019;22(5):604-22. doi: |
| 1142 | 10.1080/13676261.2018.1523541 |
| 1143 | 198. Bengtsson M, Sjöblom Y, Öberg P. Young care leavers' expectations of their future: A |
| 1144 | question of time horizon. Child & Family Social Work. 2018;23(2):188-95. doi: 10.1111/cfs.12399 |
| 1145 | 199. Boman M. Relationships with family members and transition from out-of-home care: |
| 1146 | Unfinished business. Children and Youth Services Review. 2022;143:106662. doi: |
| 1147 | 10.1016/j.childyouth.2022.106662 |
| 1148 | 200. Brady E, Gilligan R. The life course perspective: An integrative research paradigm for |
| 1149 | examining the educational experiences of adult care leavers? Children and Youth Services Review. |
| 1150 | 2018;87:69-77. doi: 10.1016/j.childyouth.2018.02.019 |
| 1151 | 201. Dumollard M, Gauthier-Davies C, Goyette M, Blanchet A. The transition to adulthood of |
| 1152 | dual-system youth in Quebec: A focus on high school graduation and housing situation. Child and |
| 1153 | Adolescent Social Work Journal. 2023:Advance online publication. doi: 10.1007/s10560-023- |
| 1154 | 00944-x |
| 1155 | 202. Gabriel T, Keller S, Bombach C. Vulnerability and well-being decades after leaving care. |
| 1156 | Frontiers in Psychology. 2021;12(34):1-11. doi: 10.3389/fpsyg.2021.577450 |
| 1157 | 203. Brady E, Gilligan R. The role of agency in shaping the educational journeys of care- |
| 1158 | experienced adults: Insights from a life course study of education and care. Children & Society. |
| 1159 | 2020;34(2):121-35. doi: 10.1111/chso.12361 |

1160	204.	Höjer I, Oterholm I. Foster carers’ views of the transition from foster care to adulthood for

| 1161 | young people with mental health problems from a life-course perspective. Nordic Social Work |
| --- | --- |
| 1162 | Research. 2023:Advance online publication. doi: 10.1080/2156857X.2023.2298680 |
| 1163 | 205. Keller S, Gabriel T, Bombach C. Narratives on leaving care in Switzerland: Biographies and |
| 1164 | discourses in the 20th century. Child & Family Social Work. 2021;26(2):248-57. doi: |
| 1165 | 10.1111/cfs.12813 |
| 1166 | 206. Mendes P. Towards the social inclusion of young people transitioning from out-of-home |
| 1167 | care: An examination of the Home Stretch campaign. Social Alternatives. 2018;37(1):59-62 |
| 1168 | 207. Mølholt A-K. ‘Sometimes I feel 60, sometimes I feel 13’: Perceptions of age among |
| 1169 | vulnerable young people in Denmark. Journal of Youth Studies. 2019:1-15. doi: |
| 1170 | 10.1080/13676261.2019.1687861 |
| 1171 | 208. Power L, Raphael D. Care leavers: A British affair. Child & Family Social Work. |
| 1172 | 2018;23(3):346-53. doi: 10.1111/cfs.12421 |
| 1173 | 209. Pryce J, Napolitano L, Samuels GM. Transition to adulthood of former foster youth: |
| 1174 | Multilevel challenges to the help-seeking pProcess. Emerging Adulthood. 2017;5(5):311-21. doi: |
| 1175 | 10.1177/2167696816685231 |
| 1176 | 210. Refaeli T, Mangold K, Zeira A, Kongeter S. Continuity and discontinuity in the transition |
| 1177 | from care to adulthood. British Journal of Social Work. 2017;47(2):325-42. doi: |
| 1178 | 10.1093/bjsw/bcw016 |
| 1179 | 211. Reimer D, Schäfer D. The use of biographical narratives to explain favourable and |
| 1180 | unfavourable outcomes for children in foster care. Adoption & Fostering. 2015;39(1):5-20. doi: |
| 1181 | 10.1177/0308575914565084 |
| 1182 | 212. Spallek M, Haynes M. Post-school transitions for children in out-of-home care: Evidence |
| 1183 | from the Australian Census Longitudinal Dataset. Journal of Youth Studies. 2021;24(7):901-24. |
| 1184 | doi: 10.1080/13676261.2020.1781803 |

1185	213.	Bengtsson M, Sjöblom Y, Öberg P. Transitional patterns when leaving care: Care leavers’

| 1186 | agency in a longitudinal perspective. Children and Youth Services Review. 2020;118:105486. doi: |
| --- | --- |
| 1187 | 10.1016/j.childyouth.2020.105486 |
| 1188 | 214. Solem M-B, Helgeland IM, Brannen J, Phoenix A. Transitions to adulthood of ‘at risk’ |
| 1189 | young men: New analysis from two Norwegian qualitative longitudinal studies. Children & Society. |
| 1190 | 2020;34(3):220-33. doi: 10.1111/chso.12369 |
| 1191 | 215. Achdut N, Benbenishty R, Zeira A. Labor market position among care leavers and their |
| 1192 | matched peers: A longitudinal comparative study. Child Abuse & Neglect. 2023;145:106406. doi: |
| 1193 | <https://doi.org/10.1016/j.chiabu.2023.106406> |
| 1194 | 216. Brady E, Gilligan R. Supporting care-experienced adults' educational journeys: “Linked |
| 1195 | lives” over the life course. Child & Family Social Work. 2020;25(2):221-9. doi: 10.1111/cfs.12677 |
| 1196 | 217. Gilligan R, Brady E. What helps adult care leavers return to education? Exploring the |
| 1197 | relevance of learner identity and the life course perspective. Journal of Youth Studies. 2022:1-14. |
| 1198 | doi: 10.1080/13676261.2022.2098706 |
| 1199 | 218. Dumont A, Lanctôt N, Paquette G. "I had a shitty past; I want a great future": Hopes and |
| 1200 | fears of vulnerable adolescent girls aging out of care. Children & Youth Services Review. |
| 1201 | 2022;134:106374. doi: 10.1016/j.childyouth.2022.106374 |
| 1202 | 219. Lanctôt N, Turcotte M. The ‘good mother’ struggles: Obstacles to the attainment of |
| 1203 | motherhood ideals among adult women formerly placed in residential care. Child & Family Social |
| 1204 | Work. 2018;23(1):80-7. doi: 10.1111/cfs.12386 |
| 1205 | 220. Paulsen V, Thomas N. The transition to adulthood from care as a struggle for recognition. |
| 1206 | Child & Family Social Work. 2018;23(2):163-70. doi: 10.1111/cfs.12395 |
| 1207 | 221. Abdullah A, Cudjoe E, Emery CR, Frederico M. Moving towards independent living in |
| 1208 | Ghana: Narratives from young adults about their kinship care experience. Journal of Adolescence. |
| 1209 | 2020;79:148-56. doi: 10.1016/j.adolescence.2020.01.005 |

1210	222.	Bengtsson M, Sjöblom Y, Öberg P. ‘Well, it’s up to me now’: Young care leavers’

| 1211 | strategies for handling adversities when leaving out-of-home care in Sweden. Nordic Social Work |
| --- | --- |
| 1212 | Research. 2018;8(sup1):8-18. doi: 10.1080/2156857X.2018.1428673 |
| 1213 | 223. Berridge D. Driving outcomes: Learning to drive, resilience and young people living in |
| 1214 | residential care. Child & Family Social Work. 2017;22(1):77-85. doi: 10.1111/cfs.12198 |
| 1215 | 224. Bodiroa A. Challenges, agency and ecology in the transition of youth from institutional care |
| 1216 | to independent living in Johannesburg [dissertation]. Johannesburg, South Africa: University of |
| 1217 | Johannesburg; 2021. |
| 1218 | 225. Crous G, Montserrat C, Gallart-Mir J, Matás M. ‘In the end you’re no longer the kid from |
| 1219 | the children’s home, you‘re just yourself’: Resilience in care leavers over 25. European Journal of |
| 1220 | Social Work. 2021;24(5):896-909. doi: 10.1080/13691457.2021.1918063 |
| 1221 | 226. Dima G, Bucuta MD. The process of transition from public care to independent living: A |
| 1222 | resilience-based approach. Revista de Cercetare si Interventie Sociala. 2015;50:53-65 |
| 1223 | 227. Frimpong-Manso KA. Building and utilising resilience: The challenges and coping |
| 1224 | mechanisms of care leavers in Ghana. Children and Youth Services Review. 2018;87:52-9. doi: |
| 1225 | 10.1016/j.childyouth.2018.02.016 |
| 1226 | 228. Frimpong-Manso KA. Stories of care-leaving: The experiences of a group of resilient young |
| 1227 | adults on their journey to interdependent living in Ghana. Emerging Adulthood. 2020;8(1):16-25. |
| 1228 | doi: 10.1177/2167696818807114 |
| 1229 | 229. Furey R, Harris-Evans J. Work and resilience: Care leavers' experiences of navigating |
| 1230 | towards employment and independence. Child & Family Social Work. 2021;26(3):404-14. doi: |
| 1231 | 10.1111/cfs.12822 |
| 1232 | 230. Greeson JKP, Gyourko JR, Jaffee SR, Wasch S. The experiences of older youth in and aged |
| 1233 | out of foster care during the COVID-19 pandemic: Material and financial well-being by foster care |
| 1234 | status, gender identity, sexual orientation, ethnicity, and race. American Journal of Orthopsychiatry. |
| 1235 | 2022;92(3):334-48. doi: 10.1037/ort0000615 |

1236	231.	Häggman-Laitila A, Salokekkilä P, Satka M, Toivonen K, Kekolahti P, Ryynänen O-P. The

| 1237 | coping of young Finnish adults after out-of-home care and aftercare services: A document-based |
| --- | --- |
| 1238 | analysis. Children and Youth Services Review. 2019;102:150-7. doi: |
| 1239 | 10.1016/j.childyouth.2019.05.009 |
| 1240 | 232. Hamilton DJ, Taylor BJ, Killick C, Bickerstaff D. Suicidal Ideation and behaviour among |
| 1241 | young people leaving care: Case-file survey. Child Care in Practice. 2015;21(2):160-76. doi: |
| 1242 | 10.1080/13575279.2014.994475 |
| 1243 | 233. Hlungwani J, van Breda AD. Affording managed opportunities for independence to build |
| 1244 | looked-after young people's resilience: Perceptions and experiences of care workers. Journal of |
| 1245 | Children's Services. 2022;17(2):137-51. doi: 10.1108/JCS-10-2021-0044 |
| 1246 | 234. Isakov AB, Hrnčić J. Preparedness for emancipation of youth leaving alternative care in |
| 1247 | Serbia. International Journal of Child, Youth & Family Studies. 2018;9(1):83-107. doi: |
| 1248 | 10.18357/ijcyfs91201818121 |
| 1249 | 235. Melkman E, Refaeli T, Bibi B, Benbenishty R. Readiness for independent living among |
| 1250 | youth on the verge of leaving juvenile correctional facilities. International Journal of Offender |
| 1251 | Therapy and Comparative Criminology. 2016;60(10):1209-25. doi: 10.1177/0306624X15575117 |
| 1252 | 236. Mendes P, Standfield R, Saunders B, McCurdy S, Walsh J, Turnbull L. Aboriginal and |
| 1253 | Torres Strait Islander (Indigenous) young people leaving out-of-home care in Australia: A national |
| 1254 | scoping study. Children and Youth Services Review. 2021;121:105848. doi: |
| 1255 | 10.1016/j.childyouth.2020.105848 |
| 1256 | 237. Moore J, Flynn M, Morgan M. Social ecological resilience and mental wellbeing of Irish |
| 1257 | emigrant survivors of clerical institutional childhood abuse. Child Abuse Review. 2019;28(1):52- |
| 1258 | 68. doi: 10.1002/car.2548 |
| 1259 | 238. Moore J, Thornton C, Hughes M. On the road to resilience: The help-seeking experiences of |
| 1260 | Irish emigrant survivors of institutional abuse. Child Abuse Review. 2017;26(5):375-87. doi: |
| 1261 | 10.1002/car.2415 |

1262	239.	Nho CR, Park EH, McCarthy ML. Case studies of successful transition from out-of-home

| 1263 | placement to young adulthood in Korea. Children and Youth Services Review. 2017;79:315-24. |
| --- | --- |
| 1264 | doi: 10.1016/j.childyouth.2017.06.035 |
| 1265 | 240. Pinkney S, Walker G. ‘It was me, but it was them that helped me’: Exploring the issues for |
| 1266 | care experienced young people within higher education. Children and Youth Services Review. |
| 1267 | 2020;108:104576. doi: <https://doi.org/10.1016/j.childyouth.2019.104576> |
| 1268 | 241. Rácz A. Alternative care of children without family in Hungary. Revista de Asistenta |
| 1269 | Sociala. 2018;17(3):23-32 |
| 1270 | 242. Radey M, Boel-Studt S, Collins CJ. “Taking it day by day”: Maternity group homes in the |
| 1271 | lives of mothers aging out of foster care. Children and Youth Services Review. 2023;155:107293. |
| 1272 | doi: 10.1016/j.childyouth.2023.107293 |
| 1273 | 243. Refaeli T. Narratives of care leavers: What promotes resilience in transitions to independent |
| 1274 | lives? Children and Youth Services Review. 2017;79:1-9. doi: 10.1016/j.childyouth.2017.05.023 |
| 1275 | 244. Samarah EMS, Schelbe L, Jackson LA. A photovoice study of college students who have |
| 1276 | experienced foster care, relative care, and/or homelessness. Children and Youth Services Review. |
| 1277 | 2023;151:107042. doi: 10.1016/j.childyouth.2023.107042 |
| 1278 | 245. Sekibo B. Experiences of young people early in the transition from residential care in Lagos |
| 1279 | State, Nigeria. Emerging Adulthood. 2020;8(1):92-100. doi: 10.1177/2167696818822232 |
| 1280 | 246. Simpson D, Murphy SF. ‘So we beat on, boats against the current, borne back ceaselessly |
| 1281 | into the past’: Legacy, care leavers and university study. Journal of Youth Studies. 2022;25(2):259- |
| 1282 | 74. doi: 10.1080/13676261.2020.1865527 |
| 1283 | 247. Stein M. Supporting young people from care to adulthood: International practice. Child & |
| 1284 | Family Social Work. 2019;24(3):400-5. doi: 10.1111/cfs.12473 |
| 1285 | 248. Stephens T, Aparicio EM. “It's just broken branches”: Child welfare-affected mothers' dual |
| 1286 | experiences of insecurity and striving for resilience in the aftermath of complex trauma and familial |

| 1287 | substance abuse. Children and Youth Services Review. 2017;73:248-56. doi: |
| --- | --- |
| 1288 | <https://doi.org/10.1016/j.childyouth.2016.11.035> |
| 1289 | 249. Sulimani-Aidan Y. ‘She was like a mother and a father to me’: Searching for the ideal |
| 1290 | mentor for youth in care. Child & Family Social Work. 2017;22(2):862-70. doi: 10.1111/cfs.12306 |
| 1291 | 250. Sulimani-Aidan Y. Promoting resilience among adolescents in care from their social |
| 1292 | workers' perspectives. Children and Youth Services Review. 2018;94:43-8. doi: |
| 1293 | 10.1016/j.childyouth.2018.09.005 |
| 1294 | 251. Syme A, Hill M. Professionals' perceptions of the rocky routes to successful outcomes for |
| 1295 | young people in a children's residential school. Child & Family Social Work. 2017;22(1):185-94. |
| 1296 | doi: 10.1111/cfs.12216 |
| 1297 | 252. Tahkola E-M, Metsäpelto R-L, Ruohotie-Lyhty M, Poikkeus A-M. Coping strategies in life |
| 1298 | stories of young adults with foster care backgrounds. European Journal of Social Work. |
| 1299 | 2021;24(5):910-21. doi: 10.1080/13691457.2021.1954885 |
| 1300 | 253. Turner L, Percy-Smith B. Care leavers and children's services: Exploring the utility of |
| 1301 | communities of practice in theorising transition. British Journal of Social Work. 2020;50(6):1758- |
| 1302 | 74. doi: 10.1093/bjsw/bcz108 |
| 1303 | 254. Turner R, Vira EG, Bergström M, Olsson TM. Cultural adaptation of interventions and the a |
| 1304 | priori assessment of intervention fit: Exploring measurement invariance for American and Swedish |
| 1305 | youth leaving care. Journal of Evidence-Based Social Work. 2023;20(3):306-22. doi: |
| 1306 | 10.1080/26408066.2022.2159602 |
| 1307 | 255. Van Breda AD. The Youth Ecological-Resilience Scale: A partial validation. Research on |
| 1308 | Social Work Practice. 2017;27(2):248-57. doi: 10.1177/1049731516651731 |
| 1309 | 256. Van Breda AD. The first two years out of residential care in South Africa: A critical period |
| 1310 | for care-leaving services. Child Welfare. 2018;95(6):63-82 |
| 1311 | 257. Webb L, Cox N, Cumbers H, Martikke S, Gedzielewski E, Duale M. Personal resilience and |
| 1312 | identity capital among young people leaving care: enhancing identity formation and life chances |

| 1313 | through involvement in volunteering and social action. Journal of Youth Studies. 2017;20(7):889- |
| --- | --- |
| 1314 | 903. doi: 10.1080/13676261.2016.1273519 |
| 1315 | 258. Wilke NG, Roberts M, Mitchell T, Howard AH. Spirituality as a protective factor: A |
| 1316 | multinational study of 267 adults separated from parental care as children. Social Work and |
| 1317 | Christianity. 2023;50(2):191-212. doi: 10.3403/swc.v50i2.309 |
| 1318 | 259. Bramsen I, Kuiper C, Willemse K, Cardol M. My path towards living on my own: Voices of |
| 1319 | youth leaving Dutch secure residential care. Child & Adolescent Social Work Journal. |
| 1320 | 2019;36(4):365-80. doi: 10.1007/s10560-018-0564-2 |
| 1321 | 260. Hyde R, Atkinson C. Care leavers' priorities and the corporate parent role: A self- |
| 1322 | determination theory perspective. Educational & Child Psychology. 2019;36(1):40-57. doi: |
| 1323 | 10.53841/bpsecp.2019.36.1.40 |
| 1324 | 261. Itzhaki-Braun Y, Sulimani-Aidan Y. Determination of life satisfaction among young women |
| 1325 | care leavers from the Ultraorthodox Jewish community. Children and Youth Services Review. |
| 1326 | 2022;136:106428. doi: 10.1016/j.childyouth.2022.106428 |
| 1327 | 262. Jackson MS, Colvin AD, Bullock AN. Strategies to address mental health challenges of |
| 1328 | foster youth transitioning to college. Best Practices in Mental Health. 2019;15(1):20-31. doi: |
| 1329 | 10.1007/s10560-022-00905-w |
| 1330 | 263. Berejena Mhongera P, Lombard A. Who is there for me? Evaluating the social support |
| 1331 | received by adolescent girls transitioning from institutional care in Zimbabwe. Practice. |
| 1332 | 2017;29(1):19-35. doi: 10.1080/09503153.2016.1185515 |
| 1333 | 264. Cox N, Martikke S, Cumbers H, Webb L. Reinventing selves and connections through |
| 1334 | community volunteering: How care leavers and their supporters create a space for agency and self- |
| 1335 | work. Voluntary Sector Review. 2023;14(2):293–313. doi: 10.1332/204080522X16454630207045 |
| 1336 | 265. Gilligan R, Arnau-Sabatés L. The role of carers in supporting the progress of care leavers in |
| 1337 | the world of work. Child & Family Social Work. 2017;22(2):792-800. doi: 10.1111/cfs.12297 |

1338	266.	Gimeno-Monterde C, Gómez-Quintero JD, C. Aguerri J. Unaccompanied young people and

| 1339 | transition to adulthood: Challenges for child care services. Children and Youth Services Review. |
| --- | --- |
| 1340 | 2021;121:105858. doi: 10.1016/j.childyouth.2020.105858 |
| 1341 | 267. Martikke S, Cumbers H, Cox N, Webb L, Gedzielewski E, Duale M. Building bridges into |
| 1342 | the community: Social capital in a volunteering project for care leavers. Children & Society. |
| 1343 | 2019;33(2):111-25. doi: 10.1111/chso.12300 |
| 1344 | 268. Mullan P. Care leaver’s outcomes in Ireland: The role of social capital. Journal of Children's |
| 1345 | Services. 2022;17(2):97-110. doi: 10.1108/JCS-05-2021-0023 |
| 1346 | 269. Radey M, Schelbe L, McWey LM, Holtrop K. Me, myself and I: perceptions of social |
| 1347 | capital for mothers ageing out of the child welfare system. Child & Family Social Work. |
| 1348 | 2017;22(2):981-91. doi: 10.1111/cfs.12318 |
| 1349 | 270. Shotton LH. From care to caring: Using Bourdieu to explore care experienced students |
| 1350 | journeys into and through nurse education. Research in Post-Compulsory Education. |
| 1351 | 2021;26(4):442-60. doi: 10.1080/13596748.2021.1980662 |
| 1352 | 271. Tobolowsky BF, Madden EE, Scannapieco M. Living on the edge: The postsecondary |
| 1353 | journeys of foster care alumni. College Student Affairs Journal. 2017;35(1):86. doi: |
| 1354 | 10.1353/csj.2017.0007 |
| 1355 | 272. Tyrell FA, Yates TM. A growth curve analysis of housing quality among transition-aged |
| 1356 | foster youth. Child & Youth Care Forum. 2017;46(1):91-117. doi: 10.1007/s10566-016-9370-1 |
| 1357 | 273. Curry SR, Abrams LS. Housing and social support for youth aging out of foster care: State |
| 1358 | of the research literature and directions for future inquiry. Child and Adolescent Social Work |
| 1359 | Journal. 2015;32(2):143-53. doi: 10.1007/s10560-014-0346-4 |
| 1360 | 274. Melkman EP. Childhood adversity, social support networks and well-being among youth |
| 1361 | aging out of care: An exploratory study of mediation. Child Abuse & Neglect. 2017;72:85-97. doi: |
| 1362 | 10.1016/j.chiabu.2017.07.020 |

1363	275.	Melkman EP, Benbenishty R. Social support networks of care leavers: Mediating between

| 1364 | childhood adversity and adult functioning. Children and Youth Services Review. 2018;86:176-87. |
| --- | --- |
| 1365 | doi: 10.1016/j.childyouth.2018.01.040 |
| 1366 | 276. Keshri AK. Understanding orphaned young people’s experience of leaving care in India: A |
| 1367 | qualitative study. Developmental Child Welfare. 2023;5(2):79–92. doi: |
| 1368 | 10.1177/25161032231171973 |
| 1369 | 277. Radey M, Schelbe L, McWey LM, Holtrop K, Canto AI. “It's really overwhelming”: Parent |
| 1370 | and service provider perspectives of parents aging out of foster care. Children and Youth Services |
| 1371 | Review. 2016;67:1-10. doi: 10.1016/j.childyouth.2016.05.013 |
| 1372 | 278. Berejena Mhongera P, Lombard A. Poverty to more poverty: An evaluation of transition |
| 1373 | services provided to adolescent girls from two institutions in Zimbabwe. Children and Youth |
| 1374 | Services Review. 2016;64:145-54. doi: 10.1016/j.childyouth.2016.03.013 |
| 1375 | 279. Berejena Mhongera P, Lombard A. Promoting successful transitions beyond institutional |
| 1376 | care: A programme-based service delivery model linked to a case management system. Social |
| 1377 | Work/Maatskaplike Werk. 2018;54(1):53-68. doi: 10.15270/54-1-614 |
| 1378 | 280. Ungar M, Theron L. Resilience and mental health: How multisystemic processes contribute |
| 1379 | to positive outcomes. Lancet Psychiatry. 2020;7(5):441-8. doi: 10.1016/S2215-0366(19)30434-1 |
| 1380 | 281. Masten AS. Emergence and evolution of developmental resilience science over half a |
| 1381 | century. Development and Psychopathology. 2024:Advance online publication. doi: |
| 1382 | 10.1017/S0954579424000154 |
| 1383 | 282. Pinkerton J, Van Breda AD. Policy as social ecological resilience scaffolding for leaving |
| 1384 | care: A case study of South Africa. In: Mann-Feder VR, Goyette M, editors. Leaving care and the |
| 1385 | transition to adulthood: International contributions to theory, research and practice. Oxford, UK: |
| 1386 | Oxford University Press; 2019. p. 87-104. |
| 1387 | 283. Elder GH, Bailey SL. The timing of military service in men's lives. In: Aldous A, Klein DM, |
| 1388 | editors. Social stress and family development. New York City, NY: Guilford; 1988. p. 157-74. |

1389	284.	Gilligan R, Brady E. What helps adult care leavers return to education? Exploring the

| 1390 | relevance of learner identity and the life course perspective. Journal of Youth Studies. |
| --- | --- |
| 1391 | 2023;26(10):1373-86. doi: 10.1080/13676261.2022.2098706 |
| 1392 | 285. Arnett JJ. Emerging adulthood: The winding road from the late teens through the twenties. |
| 1393 | 3rd ed. New York: Oxford University Press; 2024. |
| 1394 | 286. Bond S. Care leavers’ and their care workers’ views of preparation and aftercare services in |
| 1395 | the Eastern Cape, South Africa. Emerging Adulthood. 2020;8(1):26–34. doi: |
| 1396 | 10.1177/2167696818801106 |
| 1397 | 287. Gomez RJ, Ryan TN, Norton CL, Jones C, Galán-cisneros P. Perceptions of learned |
| 1398 | helplessness among emerging adults aging out of foster care. Child & Adolescent Social Work |
| 1399 | Journal. 2015;32(6):507-16. doi: 10.1007/s10560-015-0389-1 |
| 1400 | 288. Bowlby J. A secure base: Parent-child attachment and healthy human development. London: |
| 1401 | Routledge; 1988. |
| 1402 | 289. McSherry D, Fargas Malet M. The extent of stability and relational permanence achieved |
| 1403 | for young children in care in Northern Ireland. Children Australia. 2018;43(2):124-34. doi: |
| 1404 | 10.1017/cha.2018.18 |
| 1405 | 290. Field J. Social capital. 3rd ed: Routledge; 2016. |
| 1406 | 291. Bronfenbrenner U. The ecology of human development. Cambridge, MA: Harvard |
| 1407 | University Press; 1979. |
| 1408 | 292. Richmond ME. What is social case work? An introductory description. New York: Russell |
| 1409 | Sage Foundation; 1922. |
| 1410 | 293. Greene RR. Human behavior theory, person-in-environment, and social work method. In: |
| 1411 | Greene RR, editor. Human behavior theory and social work practice: Routledge; 2017. p. 1-26. |
| 1412 | 294. Bodiroa A, Ross E. Challenges, agency and ecology in the transition of youth from state |
| 1413 | residential care to independent living in Johannesburg, South Africa. Child & Youth Services. |
| 1414 | 2023;44(3):328-54. doi: 10.1080/0145935X.2022.2113871 |

1415	295.	Gahagan J, Slipp N, Chowdhury R, Kirby D, Smith S, McWilliam S, et al. Reducing

| 1416 | barriers to post-secondary education among former youth in care: A scoping review. International |
| --- | --- |
| 1417 | Journal of Educational Research Open. 2023;5:100303. doi: 10.1016/j.ijedro.2023.100303 |
| 1418 | 296. Stubbs A, Baidawi S, Mendes P. Young people transitioning from out-of-home care: Their |
| 1419 | experience of informal support: A scoping review. Children and Youth Services Review. |
| 1420 | 2023;144:106735. doi: 10.1016/j.childyouth.2022.106735 |
| 1421 | 297. Hodgson D, Cordier R, Parsons L, Walter B, Chikwava F, Watts L, et al. Developing and |
| 1422 | using matrix methods for analysis of large longitudinal qualitative datasets in out-of-home-care |
| 1423 | research. International Journal of Social Research Methodology. 2024;27(2):238-48. doi: |
| 1424 | 10.1080/13645579.2022.2159323 |
| 1425 | 298. Payne M. Modern social work theory. 5th ed: Bloomsbury Publishing; 2021. |
| 1426 | 299. Gabriel T, Keller S, Bombach C. Vulnerability and well-being decades after leaving care. |
| 1427 | Frontiers in Psychology. 2021;12. doi: 10.3389/fpsyg.2021.577450 |
| 1428 | 300. Hlungwani J, van Breda AD. Affording managed opportunities for independence to build |
| 1429 | looked-after young people’s resilience: Perceptions and experiences of care workers. Journal of |
| 1430 | Children's Services. 2022;17(2):137-51. doi: 10.1108/JCS-10-2021-0044 |
| 1431 | 301. Hlungwani J, van Breda AD. Female care leavers' journey to young adulthood from |
| 1432 | residential care in South Africa: Gender‐specific psychosocial processes of resilience. Child & |
| 1433 | Family Social Work. 2020;25(4):915-23. doi: 10.1111/cfs.12776 |
| 1434 | 302. Reuben S, Van Breda AD. Care-leavers’ efforts to build resilience through relationships of |
| 1435 | belonging. Social Work/Maatskaplike Werk. 2024;60(2):339-62. doi: 10.15270/60-2-1300 |
| 1436 |  |
| 1437 | **Supporting information** |
| 1438 | **S1 Checklist. PRISMA-ScR checklist** |
| 1439 | (DOCX) |

### 1440 S1 Table. Data extraction table

1441	(DOCX)

Figure 1. PRISMA flow diagram	[Click here to access/download;Figure;Fig 1 PRISMA flow](https://www2.cloud.editorialmanager.com/pone/download.aspx?id=38373579&guid=19939515-9cac-4557-8d52-55d75ad0f479&scheme=1)[<https://www2.cloud.editorialmanager.com/pone/download.aspx?id=38373579&guid=19939515-9cac-4557-8d52-55d75ad0f479&scheme=1> diagram v8.png](https://www2.cloud.editorialmanager.com/pone/download.aspx?id=38373579&guid=19939515-9cac-4557-8d52-55d75ad0f479&scheme=1)

[Click here to access/download](https://www2.cloud.editorialmanager.com/pone/download.aspx?id=38373576&guid=37271ed9-9a3d-4a2b-a5b0-0c55b8998ec2&scheme=1)

[Supporting Information](https://www2.cloud.editorialmanager.com/pone/download.aspx?id=38373576&guid=37271ed9-9a3d-4a2b-a5b0-0c55b8998ec2&scheme=1)

[S1 Checklist. PRISMA-ScR Checklist v8.docx](https://www2.cloud.editorialmanager.com/pone/download.aspx?id=38373576&guid=37271ed9-9a3d-4a2b-a5b0-0c55b8998ec2&scheme=1)

[Click here to access/download](https://www2.cloud.editorialmanager.com/pone/download.aspx?id=38373578&guid=efb26249-a8ed-4503-bed3-97eab10746aa&scheme=1)

[Supporting Information](https://www2.cloud.editorialmanager.com/pone/download.aspx?id=38373578&guid=efb26249-a8ed-4503-bed3-97eab10746aa&scheme=1)

[S1 Table. Data Extraction Table v8.docx](https://www2.cloud.editorialmanager.com/pone/download.aspx?id=38373578&guid=efb26249-a8ed-4503-bed3-97eab10746aa&scheme=1)
